# Homogeneous Iron Porphyrin as a Molecular CO Shuttle Enables Electrochemical Multicarbon Product Formation from CO_2_

**DOI:** 10.1021/jacs.6c10179

**Published:** 2026-09-03

**Authors:** Keon Ha Hwang, Stephen P. DiLuzio, Kaitlin M. Luedecke, Ryan G. Hadt

**Affiliations:** Division of Chemistry and Chemical Engineering, Arthur Amos Noyes Laboratory of Chemical Physics, 6469 California Institute of Technology, Pasadena, California 91125, United States

## Abstract

Homogeneous molecular catalysts for electrochemical CO_2_ reduction are generally limited to two-electron-reduced C_1_ products, primarily CO. Here we propose and demonstrate a fundamentally distinct role for molecular catalysts: as reversible CO shuttles that enhance the local CO activity at the electrical double layer. Using a water-soluble iron porphyrin as a CO shuttle, we observe the formation of isotopically verified C_1_–C_4_ hydrocarbons from CO_2_ in aqueous electrolyte. These products are not observed even in CO-saturated electrolyte in the absence of the iron porphyrin, indicating that the shuttle enhances local CO activity at the electrode surface beyond what bulk saturation can provide. Electrochemical and spectroscopic data support the formation of a stable Fe^II^–CO species outside the compact layer, which releases CO upon further reduction at the electrode surface, consistent with an electrochemically gated shuttle mechanism. These findings suggest a potential design principle in which homogeneous catalysts function as molecular intermediate shuttles at the electrode surface and provide an alternative framework for interpreting beyond-CO products observed in heterogenized molecular catalyst systems.

## Graphical abstract

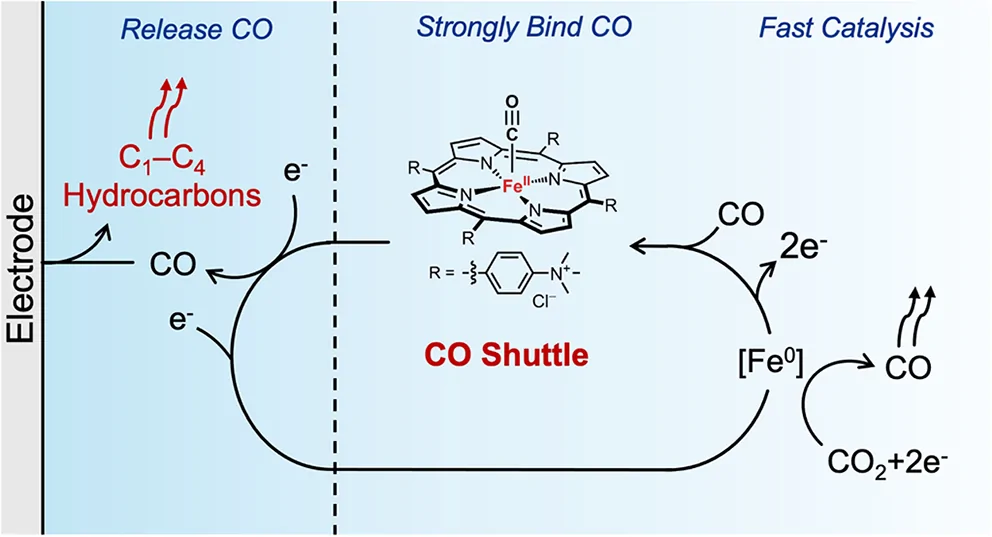

## Introduction

Electrochemical reduction of CO_2_ to value-added chemicals and fuels using renewable electricity is an attractive strategy for sustainable carbon utilization.^1^ Efficient conversion of CO_2_ to two-electron products such as CO and formate has been demonstrated across a wide range of catalysts.^2–6^ Despite these advances, extending this chemistry to products that require further reduction or C–C bond formation remains a central challenge. While metallic copper electrodes constitute the principal systems capable of C–C coupling from CO_2_, extensive surface restructuring under reaction conditions complicates mechanistic understanding.^7^

Molecular electrocatalysts offer advantages complementary to heterogeneous systems: well-defined active sites, synthetically tunable electronic structures, and spectroscopic accessibility that enables detailed mechanistic study.^5^ A wide range of molecular catalysts have been developed that selectively reduce CO_2_ to CO or formate, with reaction mechanisms elucidated through electrochemical and spectroscopic methods.^5,8^ However, homogeneous molecular electrocatalysts capable of driving reduction beyond two-electron products remain rare and have only been demonstrated in organic solvents.^9–11^

Recently, heterogenized molecular catalysts on electrode surfaces have been reported to generate further reduction products beyond CO in aqueous solvents.^10,12–20^ In many cases, these systems employ carbon-based electrodes, which are not independently active for CO_2_ reduction; therefore, the molecular site has been proposed as the catalytically active center for further reduction. Notably, heterogenized iron tetraphenylporphyrinato chloride (FeTPP) and iron phthalocyanine (FePc) systems have been reported to generate products beyond CO, including methane, ethanol, and even hydrocarbons up to butane in aqueous solvents.^10,19,20^ These reports expand the known reactivity of these iron porphyrin and phthalocyanine catalysts beyond their typically observed CO or formate products.^6,21–25^

These observations raise key mechanistic questions about reduction beyond CO in heterogenized molecular catalyst systems. It remains unclear whether heterogenization alters the intrinsic reactivity of the molecular catalyst itself or whether the electrode surface plays a more direct role in the catalytic cycle beyond electron transfer. It is also unclear why analogous reactivity is generally not achieved under homogeneous conditions, where ligand design should in principle allow more direct modulation of the molecular environment and reactivity. Addressing these possibilities is challenging because many of the spectroscopic and electrochemical techniques commonly used to study homogeneous catalysts become less accessible once the catalysts are immobilized on electrode surfaces. Thus, the development of homogeneous model systems that exhibit reduction beyond CO would enable previously inaccessible experimental mechanistic interrogation.

*Meso*-tetra­(4′-*N,N,N*-trimethylanilinium) iron­(III) porphyrin tetrachloride (Fe-*p*-TMA) is a water-soluble derivative of FeTPP (Scheme 1). It has been studied as a highly selective homogeneous catalyst for CO_2_-to-CO conversion, achieving near-quantitative faradaic efficiency in both aqueous and organic solvents.^2,6,26^ Recent reports have suggested the formation of methane under more reducing conditions, both photochemical and electrochemical, with conflicting interpretations regarding its origin.^6,26–29^ These observations present an opportunity to elucidate whether the homogeneous molecular catalyst, Fe-*p*-TMA, is responsible for reduction beyond CO, and if so, to clarify the underlying mechanism responsible for this behavior.

**Scheme 1:**
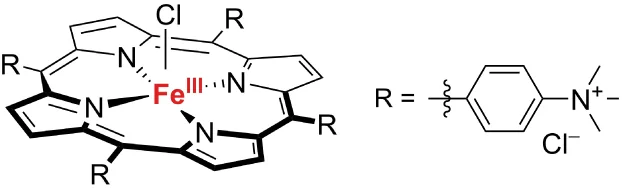
Structure of Fe-*p*-TMA

Using this homogeneous molecular system, we observe isotopically verified saturated and unsaturated light hydrocarbons (C_1_–C_4_) formed from CO_2_ reduction in aqueous electrolyte, with product yields comparable to those reported for heterogenized FePc systems.^19^ Rather than attributing these products to molecular reduction beyond CO at the iron center, we propose that Fe-*p*-TMA functions as a reversible CO shuttle that transports CO through the compact layer of the electrical double layer to the electrode surface, promoting surface-mediated reduction and chain-growth reactions by increasing local CO concentration and surface CO adsorption. This concept is expected to be general to any molecular catalyst capable of reversible CO binding. These findings offer a new framework for understanding how molecular catalysts influence surface chemistry at the electrode interface, and raise the possibility that products previously attributed to intrinsic molecular catalytic activity in heterogenized systems may arise, at least in part, from locally enhanced surface reactions.

## Results

### Formation of Multicarbon Products in Aqueous Electrolyte with a Homogeneous Catalyst

Electrochemical CO_2_ reduction in aqueous electrolyte (0.5 M KHCO_3_) in the presence of Fe-*p*-TMA at −0.9 V vs RHE produces CH_4_, C_2_H_4_, and C_2_H_6_ as gaseous products (Figure 1). The faradaic efficiencies for CH_4_, C_2_H_4_, and C_2_H_6_ are 1.7%, 0.3%, and 0.5%, respectively, along with 23.5% for CO and 56.9% for H_2_ under continuous CO_2_ flow conditions. These hydrocarbon products are not detected in the absence of Fe-*p*-TMA or when the free-base porphyrin (H_2_-*p*-TMA) is employed. A consistent product trend in efficiencies is also observed under continuous CO flow. Though the CH_4_ peak is not quantifiable due to overlap with the CO background in the gas chromatography (GC) spectra, 0.1% of C_2_H_4_ and 0.3% of C_2_H_6_ are formed only in the presence of Fe-*p*-TMA. Full faradaic efficiency information with current density is detailed in Figure S3 and Table S1. At a more negative potential of −1.0 V vs RHE under CO_2_ flow, a similar product trend is observed, but with lower faradaic efficiencies and higher current density (Figure S4 and Table S2). All controlled potential electrolysis experiments were performed using a carbon cloth working electrode, unless otherwise noted. No notable liquid products were detected. Trace formate was observed at comparable levels with and without Fe-*p*-TMA and is attributed to background electrode activity (Figure S5).

**Figure 1:**
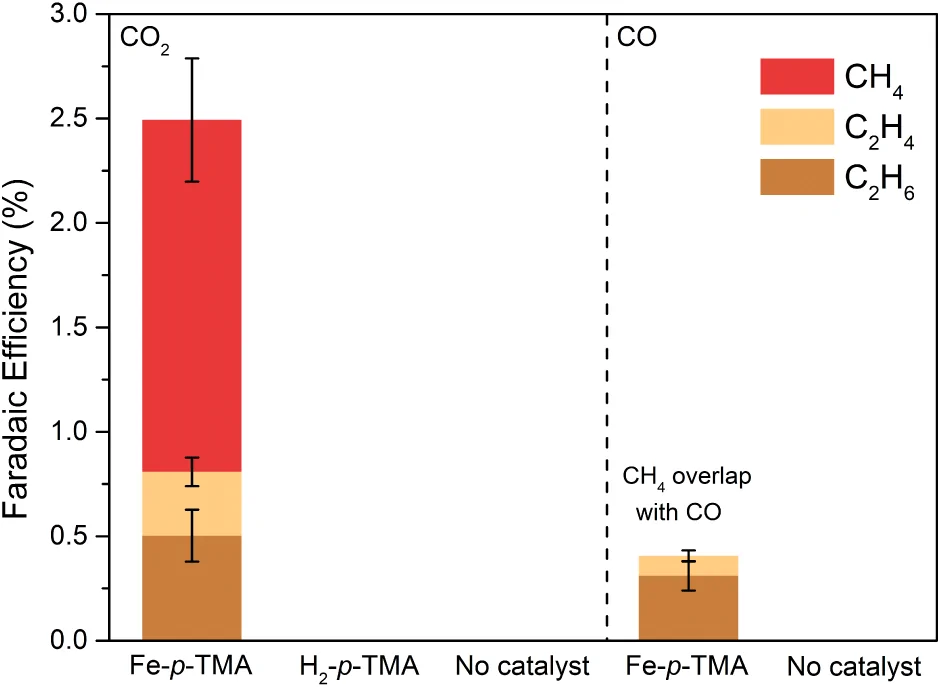
Faradaic efficiencies for CH_4_, C_2_H_4_, and C_2_H_6_ production at −0.9 V vs RHE using 0.5 mM Fe-*p*-TMA in 0.5 M KHCO_3_ electrolyte under 5 sccm CO_2_ (left) and CO (right) flow. Controls include 0.5 mM H_2_-*p*-TMA condition and catalyst-free conditions. CO and H_2_ account for the majority of the current in all cases. Error bars represent the standard deviation of three independent experiments.

To assess the sensitivity of the observed reactivity to the electrode material, two additional carbon electrodes, glassy carbon and carbon paper, were tested under otherwise identical conditions at −0.9 V vs RHE. Both showed a similar product trend, with beyond-CO products detected only in the presence of Fe-*p*-TMA (Figure S6). The glassy carbon electrode gave faradaic efficiencies comparable to those on carbon cloth (1.9% for CH_4_, 0.3% for C_2_H_4_, 0.4% for C_2_H_6_, 31.7% for CO, and 58.4% for H_2_), whereas the carbon paper electrode showed lower faradaic efficiencies for the beyond-CO products, with the current strongly dominated by hydrogen evolution (1.0% for CH_4_, 0.2% for C_2_H_4_, 0.2% for C_2_H_6_, 3.5% for CO, and 90.1% for H_2_). To examine whether beyond-CO product formation requires the positively charged ligand of Fe-*p*-TMA, the negatively charged sulfonated analogue *meso*-tetra­(4′-sulfonatophenyl)­iron­(III) porphyrin (Fe-*p*-PSULF) was tested under otherwise identical conditions (Figure S7). Fe-*p*-PSULF also produced CH_4_, C_2_H_4_, and C_2_H_6_, although at lower faradaic efficiencies than Fe-*p*-TMA (0.9% for CH_4_, 0.2% for C_2_H_4_, 0.3% for C_2_H_6_, 0.5% for CO, and 87.6% for H_2_). Formaldehyde (HCHO, 33 mM) was added to the electrolyte under CO_2_ and CO flow (Figure S8). Although the current density increased substantially, the faradaic efficiencies for beyond-CO products decreased under both conditions, making it difficult to determine whether HCHO contributes to beyond-CO product formation.

The necessity of the homogeneous porphyrin species for the reaction was corroborated by the rinse test method (Figure 2). After electrochemical reduction was performed at −0.9 V vs RHE using 0.5 mM Fe-*p*-TMA in 0.5 M KHCO_3_ electrolyte under 5 sccm CO_2_, the electrode was gently rinsed with DI water without disassembling the cell, so that the exposed surface of the electrode was maintained. After rinsing, the second electrolysis was performed under the same conditions with a blank electrolyte solution.

**Figure 2:**
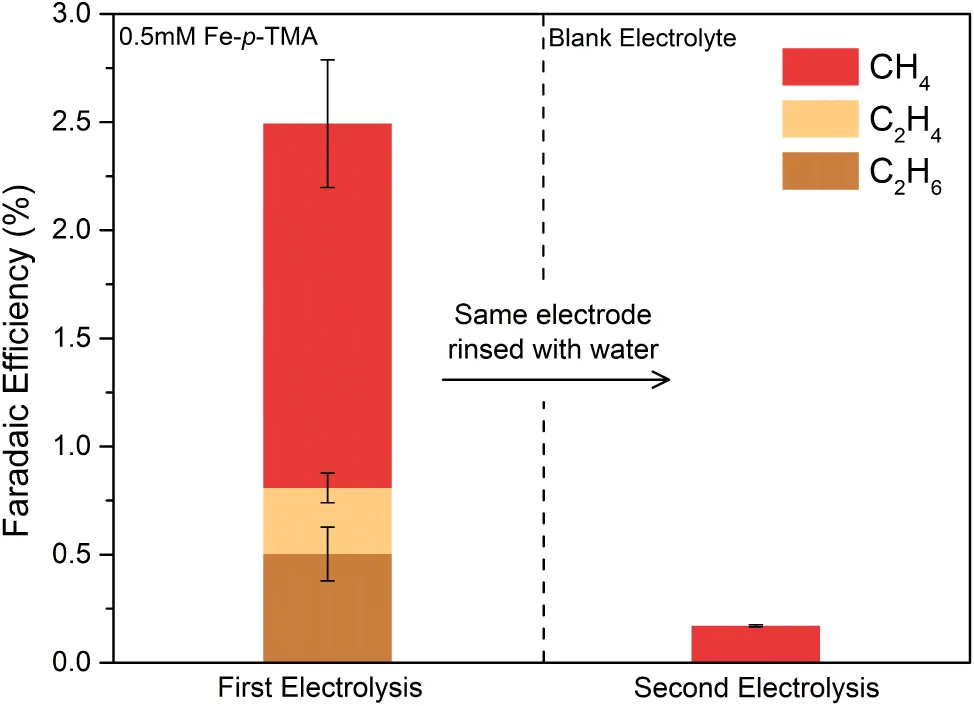
Faradaic efficiencies for CH_4_, C_2_H_4_, and C_2_H_6_ at −0.9 V vs RHE under 5 sccm CO_2_ flow. First electrolysis (left): 0.5 mM Fe-*p*-TMA in 0.5 M KHCO_3_ (aq). Second electrolysis (right): cathode chamber rinsed and refilled with 0.5 M KHCO_3_ without Fe-*p*-TMA while maintaining the assembled cell. CO and H_2_ account for the majority of the current in both cases. Error bars represent the standard deviation of three independent experiments.

If the reaction were solely catalyzed by heterogeneous catalyst-derived species adsorbed or deposited on the working electrode, the second electrolysis would show increased or maintained faradaic efficiencies.^30^ Instead, the formation of CH_4_ decreased by an order of magnitude, and C_2_ products were not detected in the second electrolysis, which indicates that the homogeneous catalyst species are required for the observed reactivity. Full faradaic efficiency information with current density is detailed in Figure S9a. No notable liquid products outside trace formate were detected after the second electrolysis (Figure S9b).

Batch electrolysis in a CO_2_-saturated environment using 0.5 mM Fe-*p*-TMA in 0.5 M KHCO_3_ electrolyte at −1.0 V vs RHE was performed for gas chromatography–mass spectrometry (GC-MS) analysis. Direct gas-injection GC analysis of the headspace further reveals the formation of higher-order hydrocarbons, including C_3_–C_4_ species such as propylene, propane, butylene and butane (Figure 3a). The faradaic efficiencies are 1.4% for CH_4_, 0.2% for C_2_H_4_, 0.4% for C_2_H_6_, 0.3% for C_3_H_6_, 0.4% for C_3_H_8_, 0.3% for C_4_H_8_, and 0.2% for C_4_H_10_. The distribution of C_2_–C_4_ hydrocarbon products follows Anderson–Schulz–Flory-type behavior (Figure 3b).^31^ The logarithm of the molar fraction of products with a given carbon number was plotted against carbon number. The observed linear relationship is expected when successive chain-growth steps occur with similar probabilities and is therefore consistent with a chain-propagation process rather than the independent formation of each hydrocarbon. The homogeneous Fe-*p*-TMA system shows lower C_1_ and higher C_2_–C_4_ yields compared to the C_1_–C_4_ products reported for heterogenized FePc systems in 0.1 M KHCO_3_ at −1.0 V vs RHE (3.9% for CH_4_, 0.2% for C_2_H_6_, 0.1% for C_3_H_6_, 0.2% for C_3_H_8_, and 0.1% for C_4_H_10_).^19^ To confirm the origin of the carbon atoms, isotopic labeling experiments using ^13^CO_2_ and NaH^13^CO_3_ were performed under the same conditions, and the headspace was analyzed with GC-MS. Bicarbonate was also isotopically labeled because there is a fast equilibrium between CO_2_ and bicarbonate in water. The shifts in the characteristic MS data confirm that all detected C_1_–C_4_ hydrocarbons originate from CO_2_. The results for CH_4_ and C_4_H_10_ are shown in Figure 3c–f, and the results for C_2_H_4_, C_2_H_6_, C_3_H_6_, C_3_H_8_, and C_4_H_8_ are detailed in Figures S10 and S11.

**Figure 3:**
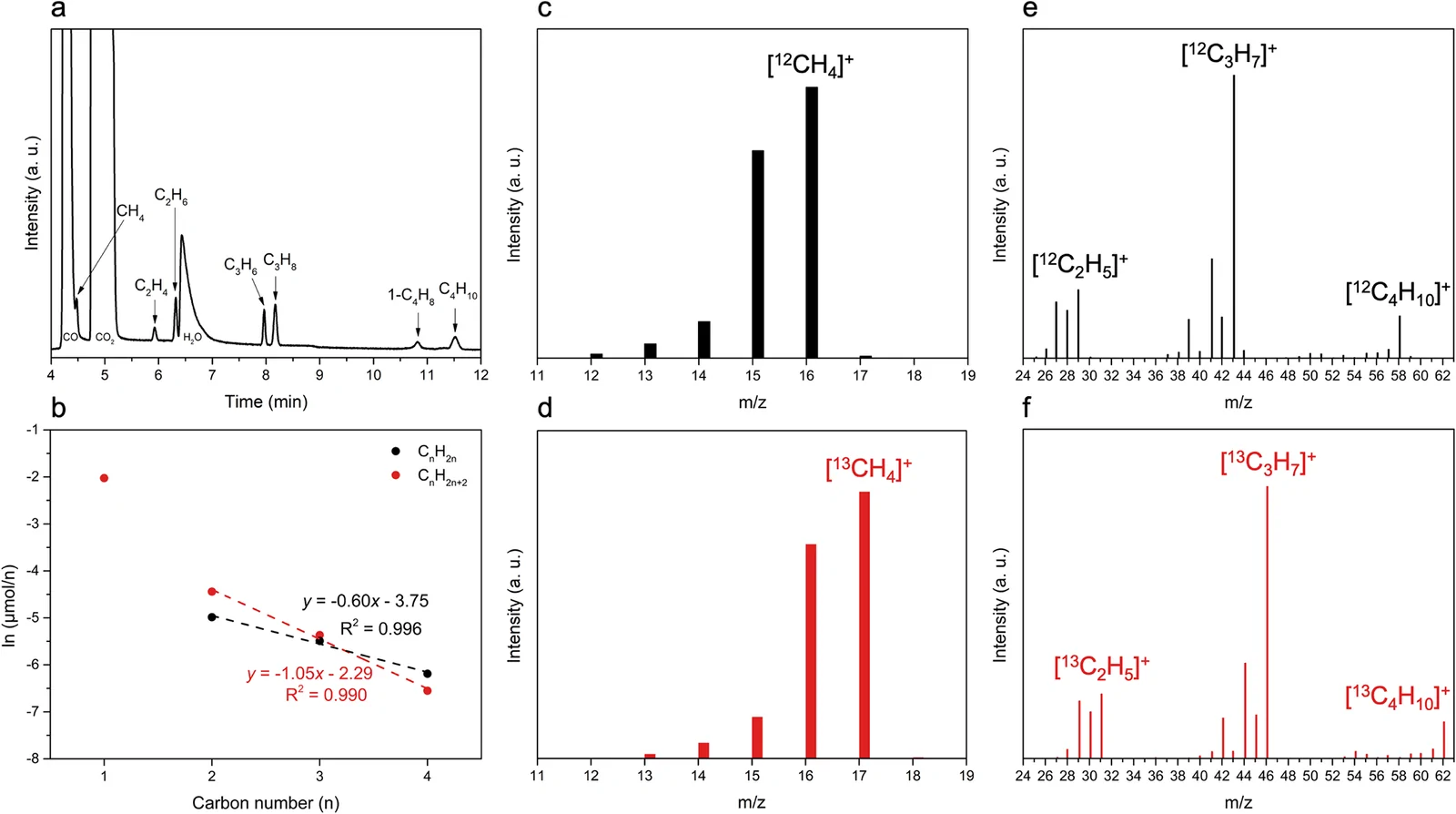
Gas chromatography and isotopic labeling experiments. (a) Gas chromatogram of the headspace and (b) Anderson–Schulz–Flory plot for the C_1_–C_4_ products from 0.5 mM Fe-*p*-TMA in 0.5 M KHCO_3_ in a CO_2_-saturated batch cell after electrolysis at −1.0 V vs RHE. Mass spectra of (c, d) methane and (e, f) butane under (c, e) ^12^CO_2_ with 0.5 M KHCO_3_ and (d, f) ^13^CO_2_ with 0.5 M NaH^13^CO_3_ under otherwise identical conditions.

Concentration dependence was studied by varying the concentration of Fe-*p*-TMA from 0 to 1 mM in 0.5 M KHCO_3_ electrolyte at −0.9 V vs RHE under 5 sccm CO_2_ flow (Figure 4). The formation of CH_4_, C_2_H_4_, and C_2_H_6_ exhibits a maximum at 0.25 mM Fe-*p*-TMA concentration and decreases at higher concentrations. The yield of CO increases linearly with concentration. These data indicate that reduction beyond CO is not linearly correlated to catalyst concentration or CO production. No liquid products apart from trace formate were detected at any concentration (Figure S12).

**Figure 4:**
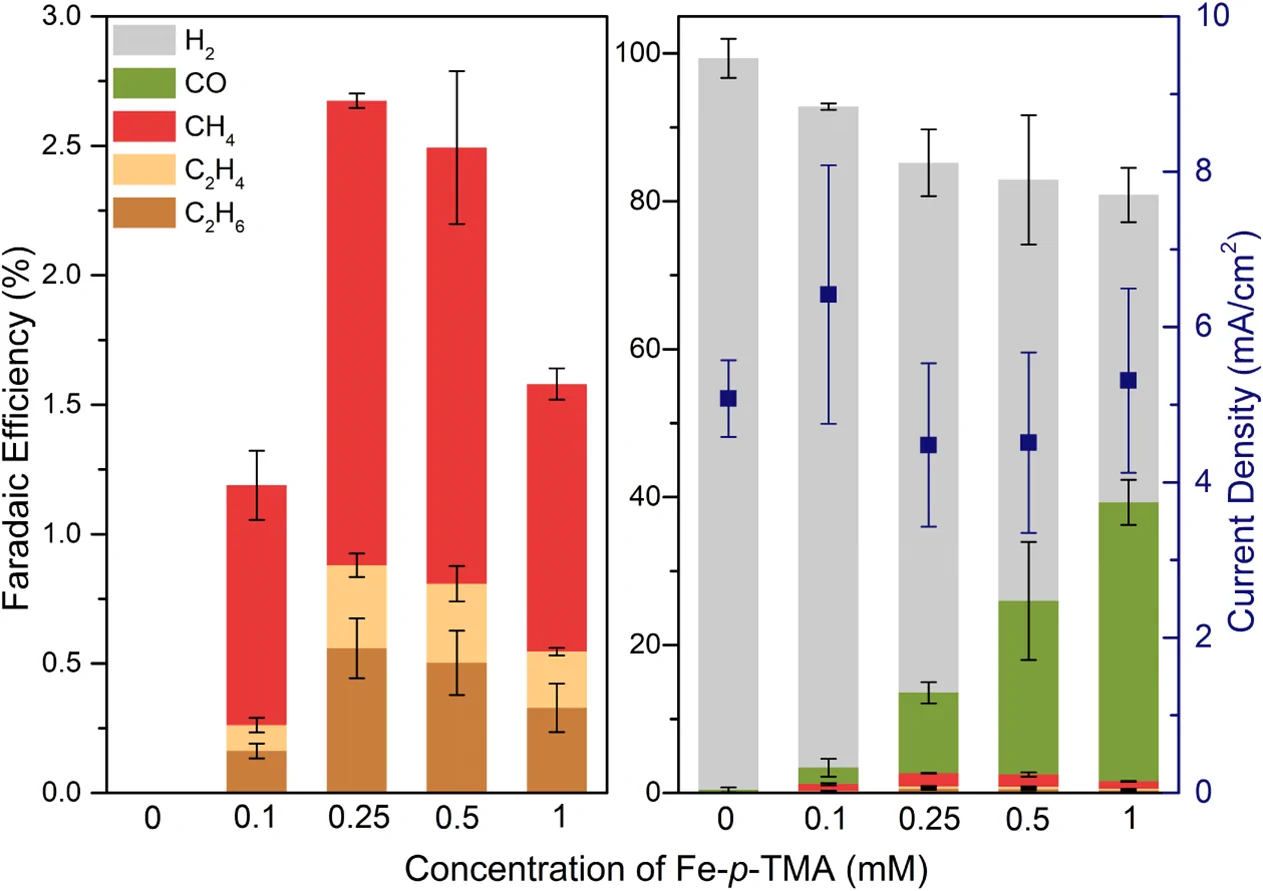
Faradaic efficiencies as a function of Fe-*p*-TMA concentration (0–1 mM) in 0.5 M KHCO_3_ (aq) under 5 sccm CO_2_ flow at −0.9 V vs RHE on a carbon cloth electrode for CH_4_, C_2_H_4_, and C_2_H_6_ (left) and for all gaseous products with current density (right). Error bars represent the standard deviation of three independent experiments.

Catalyst stability was evaluated under extended electrolysis with 0.5 mM Fe-*p*-TMA in 0.5 M KHCO_3_ electrolyte at −0.9 V vs RHE under 5 sccm CO_2_ flow (Figure 5). Full faradaic efficiency data with current density are reported in Figure S13. The initial low faradaic efficiency at 20 min arises from the GC inlet gas not yet reaching equilibrium with the product gas. A gradual decrease in the formation of C–C coupled products was observed over the course of electrolysis. Degradation of the homogeneous porphyrin was evidenced by the decreased UV–vis absorption intensity after 3 h and changes in the ^1^H NMR spectra after electrolysis across all conditions (Figures S5, S12, and S14). Such behavior is consistent with previously reported instability of homogeneous metalloporphyrins under strongly reducing conditions.^26,32,33^ The carbon cloth electrode was characterized before and after electrolysis by scanning electron microscopy paired with energy-dispersive X-ray spectroscopy (SEM-EDS) and X-ray photoelectron spectroscopy (XPS) (Figures S15 and S16), which showed iron deposition on the surface. The iron content was 0.04 at.% after 30 min and 0.19 at.% after 3 h of electrolysis by SEM-EDS and was 0.15 at.% and 0.83 at.% relative to carbon over the same intervals by XPS. Other trace elements detected were K, Ni, and Si, which originate from the electrolyte, trace metals in the carbon electrode, and dust, respectively.

**Figure 5:**
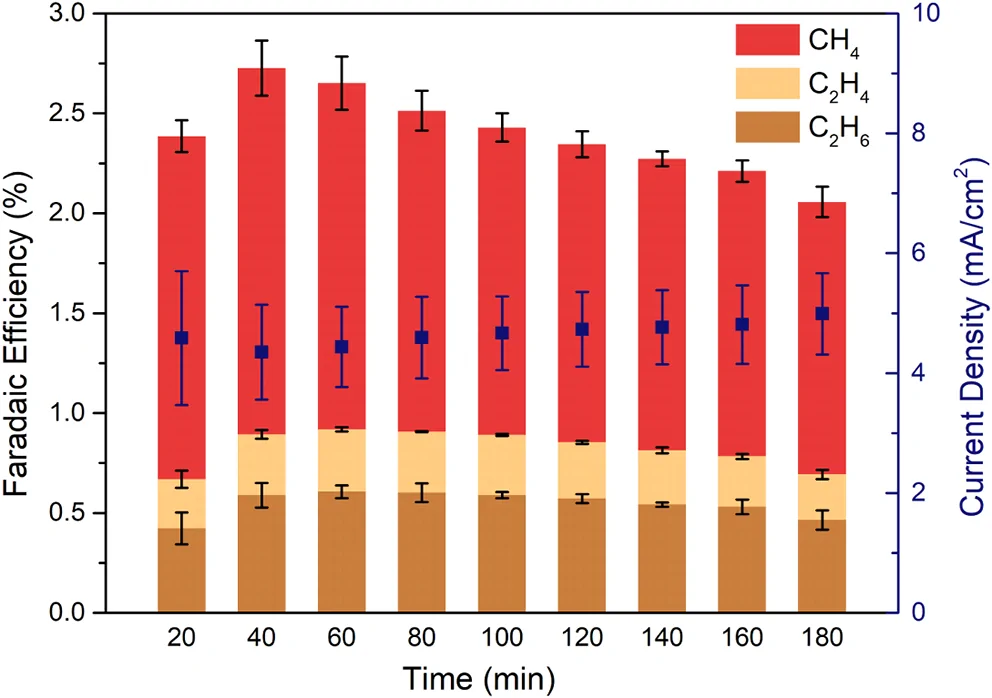
Faradaic efficiencies for CH_4_, C_2_H_4_, and C_2_H_6_ production and current density as a function of electrolysis time at −0.9 V vs RHE using 0.5 mM Fe-*p*-TMA in 0.5 M KHCO_3_ electrolyte under 5 sccm CO_2_ flow on a carbon cloth electrode. Data collected in 20 min intervals over 3 h. Error bars represent the standard deviation of three independent experiments.

However, the observed behavior argues against species generated by catalyst decomposition—either in solution or deposited on the electrode—as the sole origin of reduction beyond CO. If decomposition products themselves were solely responsible, beyond-CO products formation would be expected to increase proportionally with Fe-*p*-TMA concentration, which is not observed. Likewise, if decomposition-derived material deposited on the electrode were solely responsible, the rinse test would be expected to show higher or maintained activity (Figure 2), and beyond-CO product formation would be expected to persist or increase during the long-term electrolysis. Together, these results support the conclusion that the dominant origin of C_1_–C_4_ product formation remains associated with the presence of the homogeneous porphyrin, even as gradual decomposition occurs over time.

The effect of the hydrodynamic regime was examined. In the original setup, gas bubbling through the catholyte (1.7 mL) served as the sole source of convection. To characterize the convection in this cell, the diffusion layer thickness (δ) was estimated from the limiting current for hydrogen evolution reaction with planar Pt electrode under mildly acidic conditions, following a previously reported method.^34,35^ Without stirring, δ was 320 μm; adding a stir bar rotated at 1000 rpm increased convection and reduced δ to 93 μm (Figure S17). Because δ is not well-defined at the porous carbon cloth electrode, a planar glassy carbon electrode was used in place of the carbon cloth electrode so that these values apply to the working electrode. Under these conditions, concentration dependence was reexamined by varying the concentration of Fe-*p*-TMA from 0 to 0.5 mM in 0.5 M KHCO_3_ electrolyte at −0.9 V vs RHE under 5 sccm CO_2_ flow (Figure 6). The selectivity for CO increased markedly relative to the unstirred configuration: at 0.5 mM, the faradaic efficiency for CO reached 88.2% and no multicarbon products were detected. The formation of CH_4_, C_2_H_4_, and C_2_H_6_ instead exhibited a maximum at 0.1 mM Fe-*p*-TMA (2.6% for CH_4_, 0.3% for C_2_H_4_, and 0.5% for C_2_H_6_). These products were detected at concentrations as low as 0.05 mM. This selectivity likely reflects the balance between the hydrogen evolution reaction at the electrode and the CO_2_-to-CO conversion at the molecular catalyst.

**Figure 6:**
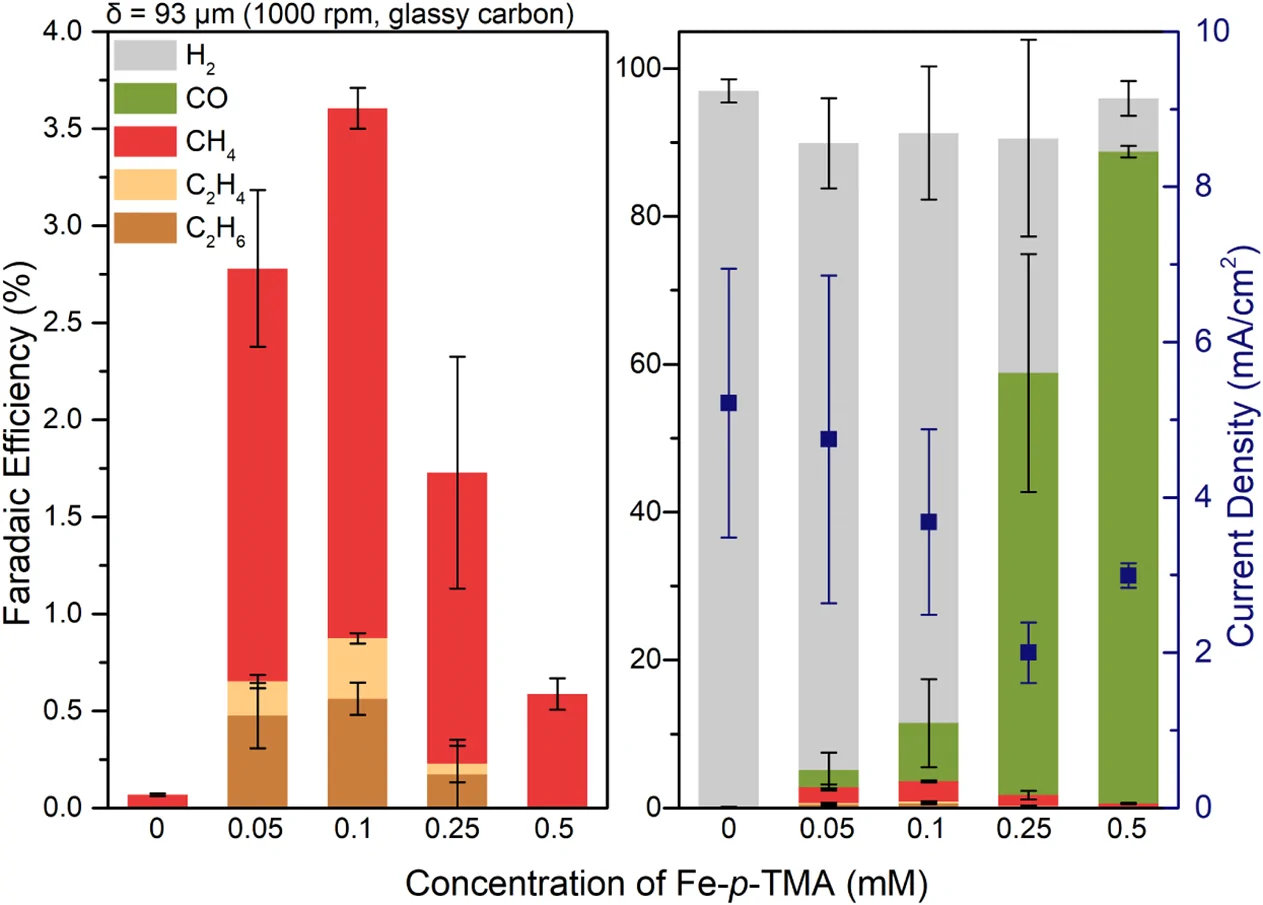
Faradaic efficiencies on a glassy carbon electrode as a function of Fe-*p*-TMA concentration (0–0.5 mM) in 0.5 M KHCO_3_ (aq) under 5 sccm CO_2_ flow at −0.9 V vs RHE for CH_4_, C_2_H_4_, and C_2_H_6_ (left) and for all gaseous products with current density (right). The stirring rate was 1000 rpm, at which the estimated diffusion layer thickness (δ) was 93 μm. Error bars represent the standard deviation of three independent experiments.

### Reversible CO Binding Ability of Fe-*p*-TMA and Fe^II^–CO Stability

Cyclic voltammograms (Figure 7) and UV–vis spectroelectrochemistry spectra (Figure S18) of Fe-*p*-TMA in 0.1 M TBAPF_6_/DMF were compared under Ar and CO atmospheres to ascertain the presence and reversibility of CO binding. These measurements were performed in DMF because the relevant redox features are obscured in aqueous electrolyte. Throughout this work, Fe^0^, Fe^I^, Fe^II^, and Fe^III^ refer to formal oxidation-state assignments for Fe-*p*-TMA species. These labels are used for convenience and do not necessarily imply that the associated redox changes are exclusively metal-centered. Under Ar atmosphere, Fe-*p*-TMA exhibits three reversible redox peaks corresponding to the sequential reductions of the complex, consistent with previous reports.^2,6,26^ The peak intensity and position are maintained during three cycles. Under CO atmosphere, Fe-*p*-TMA exhibits strong CO binding at the Fe^II^ state, as shown by the 260 mV shift in the Fe^II/I^ redox peak under CO relative to Ar atmosphere, also consistent with a previous report.^26^ UV–vis spectroelectrochemical measurements of 0.1 mM Fe-*p*-TMA in 0.1 M TBAPF_6_/DMF show a characteristic Fe^II^–CO absorption band at 419 nm, distinct from the corresponding Fe^II^ band at 428 nm under Ar atmosphere (Figure S18). Upon further reduction to the Fe^I^ state, CO dissociates from the metal center; this process has been well-studied in previous literature with FT-IR spectroelectrochemistry and is general for most Fe porphyrin systems.^24,26^ This assignment is also supported by the identical Fe^I/0^ redox peak position observed in cyclic voltammetry under Ar and CO atmospheres, together with the disappearance of the Fe^II^–CO spectroscopic signature upon reduction (Figure 7). The maintained peak intensities and positions of the Fe^II/I^ and Fe^I/0^ redox features under CO atmosphere during repeated cyclic voltammetry cycling indicate that CO binding and release are reversible and do not lead to catalyst poisoning.

**Figure 7:**
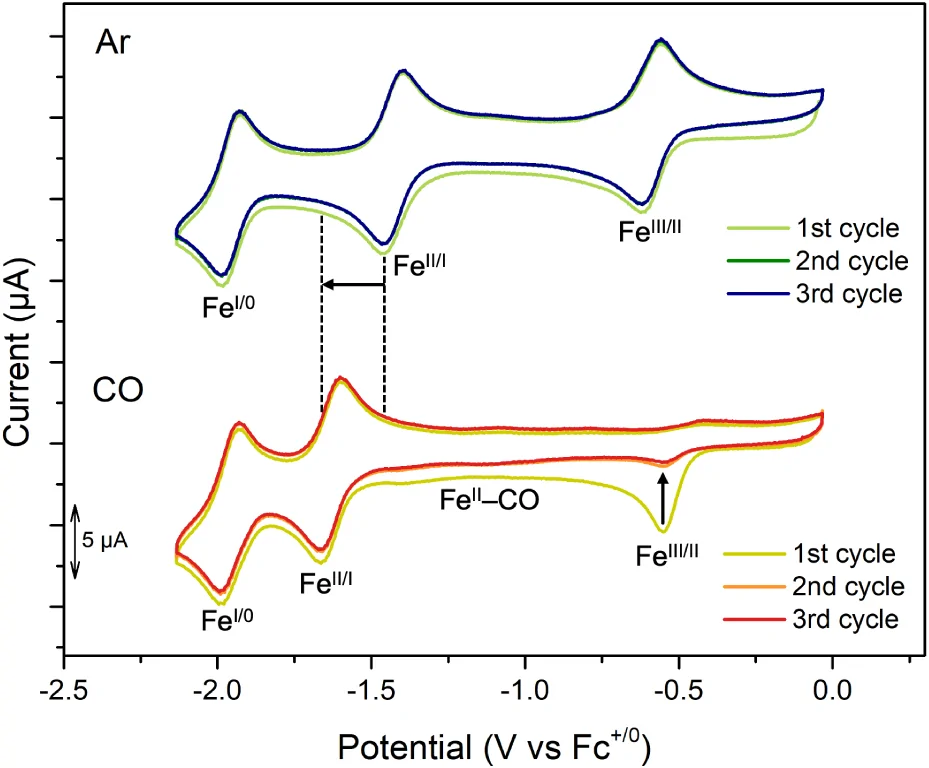
Cyclic voltammograms of 0.5 mM Fe-*p*-TMA in 0.1 M TBAPF_6_/DMF under Ar (top) and CO (bottom) atmospheres at a scan rate of 100 mV/s. Arrow indicates the negative shift of the Fe^II/I^ couple under CO.

Persistence of Fe^II^–CO under open-circuit conditions is indicated by attenuation of the Fe^III/II^ redox feature under CO during repeated electrochemical cycles, demonstrating that returning to open-circuit potential does not reoxidize the iron center or induce CO dissociation of the Fe^II^–CO. Consistent with this assignment, an oxidation feature attributed to Fe^II^–CO oxidation was observed at 0.27 V vs Fc^+/0^. When the potential window was extended beyond this feature, the Fe^III/II^ redox feature was recovered under repeated electrochemical cycles (Figure S19).

Although background hydrogen evolution partially obscured the redox features in 0.5 M KHCO_3_ aqueous electrolyte, the Fe^I/0^ and Fe^II/I^ peaks were identifiable under both Ar and CO atmospheres (Figure S20). A comparable 250 mV shift in the Fe^II/I^ redox peak under CO relative to Ar was observed in the aqueous electrolyte, indicating that the strong CO binding at the Fe^II^ state is retained in aqueous electrolyte. The electrolysis potential of −0.9 V vs RHE lies well negative of the CO-shifted Fe^II/I^ couple, so that Fe^II^–CO reaching the electrode interface is expected to undergo further reduction and release CO.

The UV–vis kinetics of the photogenerated Fe^II^–CO species provide additional support for the stability of this species in aqueous electrolyte in the absence of an applied potential (Figure 8). Photochemical generation of Fe^II^–CO via ligand-to-metal charge-transfer (LMCT) photolysis has precedent with FeTPP.^36,37^ Here Fe^II^–CO forms directly upon irradiation with 390 nm light in a 2 cm path-length cuvette containing 20 μM Fe-*p*-TMA in CO-saturated 0.5 M KHCO_3_ (aq), confirmed by the appearance of an absorption feature at 412 nm, which matches the UV–vis spectroelectrochemical signature of Fe^II^–CO generated electrochemically in aqueous solvent and is clearly distinct from that of Fe^II^ state under Ar and Fe^III^–Cl. The photogenerated Fe^II^–CO species in 0.5 M KHCO_3_ (aq) was still present after one hour. An analogous trend was observed for 0.5 mM Fe-*p*-TMA in 0.1 M TBAPF_6_ in DMF, where the characteristic Fe^II^–CO absorption band at 419 nm remained unchanged after one hour, further demonstrating the intrinsic stability of the Fe^II^–CO species once formed (Figure S21). The negative feature at 655 nm is an artifact arising from a sharp deuterium-lamp emission line in this region (Figure S22).

**Figure 8:**
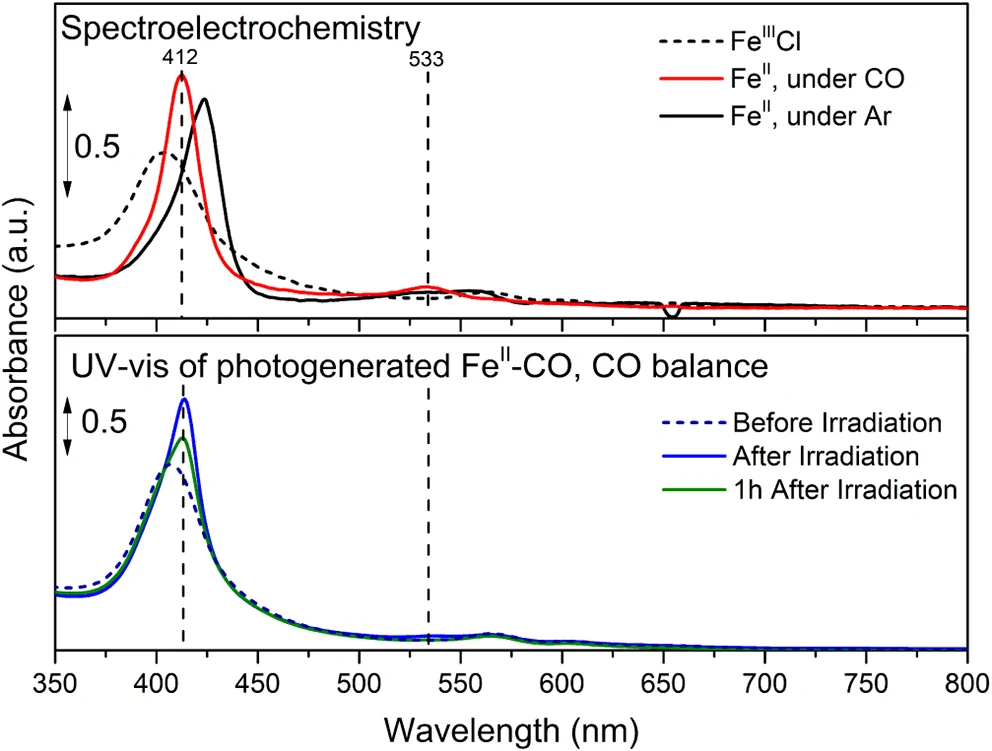
(Top) UV–vis spectroelectrochemistry of 0.1 mM Fe-*p*-TMA in 0.5 M KHCO_3_ (aq) at open-circuit potential (dashed), and at −0.64 V vs SHE under Ar (black) and CO (red). (Bottom) UV–vis of the 20 μM Fe-*p*-TMA in 0.5 M KHCO_3_ before (dashed) and after 390 nm irradiation (blue), and 1 h postirradiation (green), showing persistence of the photogenerated Fe^II^–CO species.

Collectively, these observations demonstrate that Fe-*p*-TMA can reversibly bind and release CO without deactivation. At potentials more positive than the Fe^II/I^ couple, Fe-*p*-TMA strongly binds CO in its vicinity and releases it upon encountering more negative potentials. The aqueous measurements show that this strong CO binding is retained in 0.5 M KHCO_3_ electrolyte and that Fe^II^–CO persists once formed on time scales relevant to electrolysis.

### Fe^II^–CO Formation from CO_2_-Derived CO under Catalytic Conditions

The formation and stability of Fe^II^–CO were established above under CO-saturated conditions in both DMF and aqueous electrolyte. Under the conditions where C_1_–C_4_ products are observed, however, the electrolyte is CO_2_-saturated and the current at −0.9 V vs RHE is dominated by hydrogen evolution. To determine whether Fe^II^–CO can still be formed and retained in this environment, UV–vis spectroelectrochemistry was performed in CO_2_-saturated 0.5 M KHCO_3_ (Figure 9). The Fe species were monitored at −0.2 V vs RHE (−0.63 V vs SHE), positive of the Fe^II/I^ reduction potential measured under Ar (−0.81 V vs SHE), before and after 10 min of electrolysis at −0.9 V vs RHE. Because the Fe^II/I^ couple is not proton-coupled, its potential is expected to remain approximately invariant with pH when referenced to SHE, despite the pH difference between the Ar- and CO_2_-saturated electrolytes.

**Figure 9:**
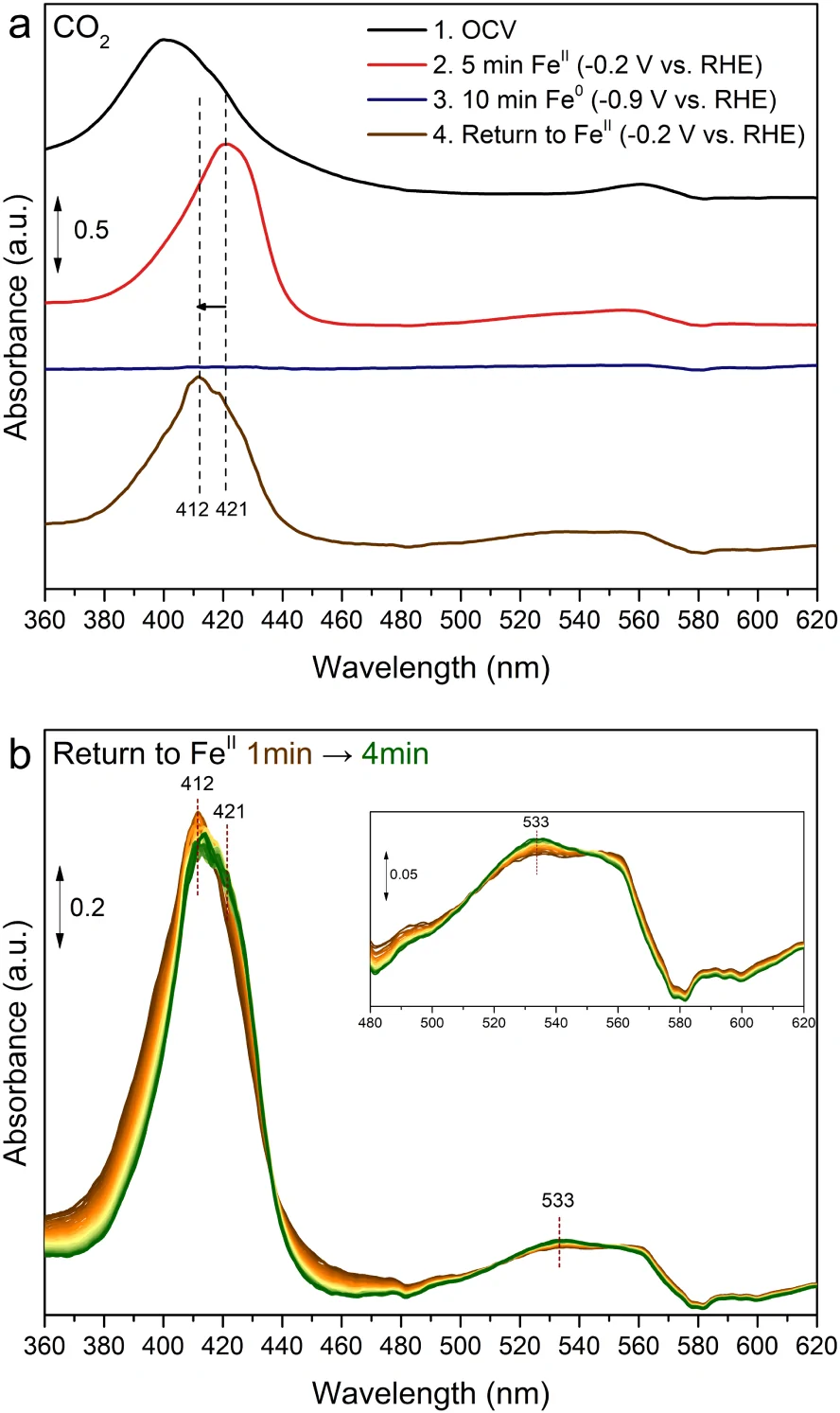
(a) UV–vis spectroelectrochemistry of 0.5 mM Fe-*p*-TMA in CO_2_-saturated 0.5 M KHCO_3_ (aq) at open-circuit potential (black), after 5 min at −0.2 V vs RHE (red), after 10 min at −0.9 V vs RHE (blue), and upon returning to −0.2 V vs RHE (brown). (b) Time-resolved UV–vis spectra recorded from 1 to 4 min after returning to −0.2 V vs RHE. Inset shows expansion of the 480–620 nm region.

At −0.2 V vs RHE, Fe-*p*-TMA is reduced to Fe^II^ and exhibits an absorption maximum at 421 nm. Upon stepping to −0.9 V vs RHE for 10 min—conditions under which CO_2_ reduction and hydrogen evolution reaction (HER) proceed simultaneously—gas evolution obscures the absorption spectrum due to light scattering. When the potential is returned to −0.2 V vs RHE, characteristic absorption features at 412 and 533 nm are observed, consistent with Fe^II^–CO species. This species forms from CO_2_-derived CO generated during the preceding reductive step. Time-resolved spectra recorded over 4 min following the potential step show that the Fe^II^–CO species is stable at −0.2 V vs RHE, with minimal spectral change over this period. Spectra extended to 1000 nm are shown in Figure S23, but no features were resolved above the noise level in the longer-wavelength region.

These results demonstrate that Fe^II^–CO forms spontaneously from CO_2_-derived CO at milder potentials where Fe-*p*-TMA is not further reduced, and that this species persists once formed, consistent with its stability under less reducing conditions relevant to catalysis.

## Discussion

### Surface-Mediated Electrochemical Fischer–Tropsch Reaction

The product distribution observed in the presence of Fe-*p*-TMA is consistent with surface-mediated chain-growth chemistry rather than molecular C–C coupling at the iron center. The C_2_–C_4_ hydrocarbon selectivity follows an Anderson–Schulz–Flory distribution, and methane formation exceeds the ideal prediction by this distribution—a characteristic of electrochemical Fischer–Tropsch systems.^31,38^ Electrochemical Fischer–Tropsch reactions proceed through hydrogenation of surface-adsorbed CO to C_1_ intermediates (commonly described as CH_2_-type species), followed by C–C coupling and chain propagation to form hydrocarbons, and have been reported only for transition metal electrodes.^31,38^ In these systems, hydrogen is delivered to surface intermediates by proton-coupled electron transfer rather than by dissociation of H_2_, and the hydrogen evolution proceeds in parallel as a competing reaction that dominates the current.^31,38^

A molecular Fischer–Tropsch pathway at the iron porphyrin is unlikely. Spectroelectrochemical measurements under catalytic conditions show no evidence for CHO-type or other highly reduced intermediates bound to Fe-*p*-TMA. Importantly, Fe-*p*-TMA is known—and further demonstrated here—to release CO upon reduction to the Fe^I^ state.^26^ Moreover, the observed product distribution, spanning C_1_–C_4_ saturated and unsaturated hydrocarbons, would require a single molecular site to mediate at least eight sequential proton–electron transfers and multiple C–C bond-forming steps, a process that would be very challenging.

Notably, methane and multicarbon yields comparable to those reported here have been observed on unmodified glassy carbon electrodes and on iron electrodes, but only under 30 atm CO_2_ pressure in aqueous KHCO_3_ electrolyte.^39,40^ At ambient pressure, most transition-metal electrodes including iron exhibit only trace or no multicarbon yields, even at potentials 0.4 V more negative than those employed in this work.^31,38,41–44^ Electrochemical Fischer–Tropsch chemistry has not been reported on conventional carbon electrodes under ambient pressure. It has been proposed that the high pressure enhances CO adsorption at the electrode, thereby enabling the CO reduction.^39,40,44^ Table S3 summarizes reported C_1_–C_4_ yields on carbon and iron electrodes.

CO reduction is not generally expected for ideal graphitic basal-plane carbon sites. However, by analogy to hydrogen evolution on carbon electrodes, where edge/defect sites and adventitious trace-metal sites can dominate apparent electrode activity,^45,46^ participation of minority electrode-associated sites cannot be excluded. Supporting this possibility, cyclic voltammetry of the catalyst-free glassy carbon electrode in 0.5 M KHCO_3_ shows substantial suppression of the hydrogen-evolution current under CO relative to Ar, consistent with CO adsorption or site blocking at HER-active electrode-associated sites (Figure S24).

These comparisons suggest that the presence of Fe-*p*-TMA enables CO-derived surface chain-growth chemistry at yields that are not otherwise accessible under ambient conditions, and that this behavior is generalizable across different carbon electrodes (Figure S6). The faradaic efficiencies for multicarbon products decrease over the course of electrolysis while the iron deposition increases (Figures 5, S15, and S16), indicating that the carbon cloth electrode itself is the principal site of this chemistry, although both iron sites and carbon electrode sites can participate. In either case, neither iron nor carbon electrodes alone produce these multicarbon products under ambient conditions; the presence of Fe-*p*-TMA creates a local environment at the electrode surface that reproduces the effect of elevated CO_2_ pressure.

### Fast Catalysis: Buildup of Local CO Concentration

The reported mechanism for electrochemical CO_2_-to-CO conversion by Fe-*p*-TMA is shown in Scheme 2.^2,6,8,26^ Three sequential single-electron reductions generate the Fe^0^ state, which rapidly binds CO_2_ and reduces it to CO with participation of a proton source, forming Fe^II^–CO. Upon further reduction to Fe^I^, CO dissociates, completing the catalytic cycle. The resting state during the catalytic cycle has been identified as Fe^I^.^26^ A recent spectroelectrochemical study of Fe-*p*-TMA in aqueous electrolyte reported an additional pathway in which CO_2_ activation occurs at a milder potential assigned to the Fe^I^ state, leading to formation of Fe^II^–CO without first accessing the formal Fe^0^ state.^47^ The Fe^II^–CO formed under these conditions remained bound at this potential, and a more cathodic potential was proposed to be required for CO dissociation and catalytic turnover. Scheme 2 therefore represents the previously established Fe^0^-mediated pathway, while an additional Fe^I^-mediated route to Fe^II^–CO may operate under aqueous conditions.

**Scheme 2:**
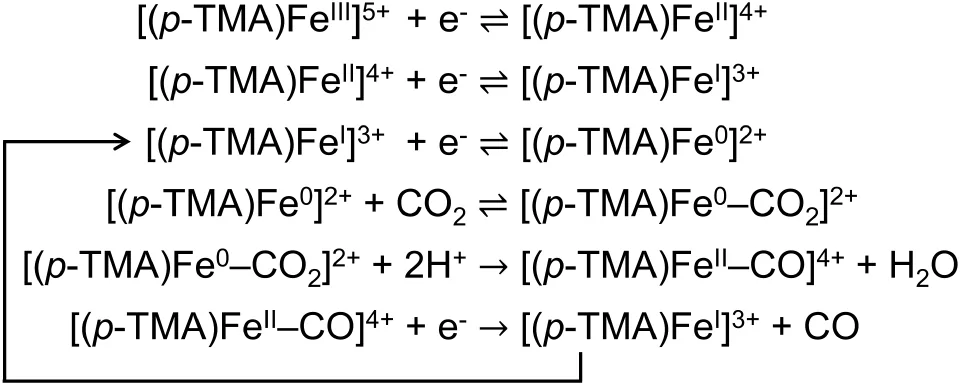
Reported Mechanism of Electrochemical CO_2_ Reduction to CO by Fe-*p*-TMA

To understand the effect of the homogeneous porphyrin on heterogeneous catalysis, we considered the spatial structure of the electrode interface. The compact layer adjacent to the electrode surface is densely packed with hydrated ions on nanometer length scales, screening the large surface charge density and confining the majority of the drop in potential to within ∼10 Å of the electrode (Scheme 3a).^48^ In the diffusion layer beyond the compact layer, the electric field is substantially screened and ions diffuse freely. In homogeneous electrocatalysis, the catalyst is reduced within the compact layer via outer-sphere electron transfer and mediates its chemical step in the diffusion layer. The narrow region in the diffusion layer where the chemical step happens is also called the reaction-diffusion layer.^8,49^ In heterogeneous multielectron catalysis such as CO_2_ reduction, the process proceeds through inner-sphere electron transfer, which requires reactant adsorption directly at the electrode surface.^4^ The local environment within the compact layer, including the availability of reactants such as H^+^, CO_2_, and CO at the electrode surface, is therefore a major factor governing heterogeneous catalytic activity.^50–55^

**Scheme 3:**
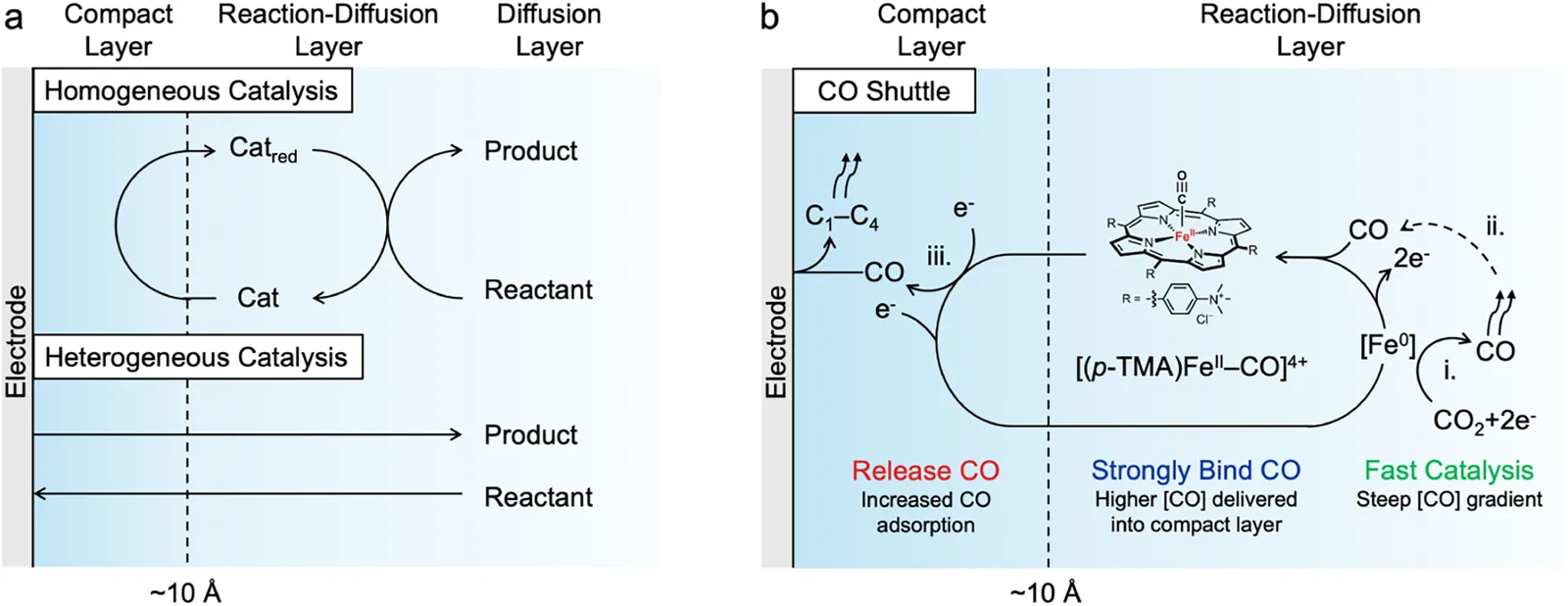
Electrocatalysis Scheme and CO Shuttling Mechanism; (a) Comparison of Reaction Zones for Homogeneous (Top) and Heterogeneous Electrocatalysis (Bottom) at the Electrical Double Layer; (b) Proposed Mechanism for Fe-*p*-TMA Functioning as an Electrochemically Gated CO Shuttle Enabling Surface-Mediated C_1_–C_4_ Product Formation

For the surface-mediated Fischer–Tropsch chemistry described above, CO must adsorb at the electrode surface. Under operating conditions, the local CO activity at the electrode is expected to be even lower than the already limited bulk saturation value of ∼1 mM, owing to both H_2_ gas evolution and the dense ionic structure of the compact layer. This limitation is especially relevant because recent studies on Cu electrodes have shown that efficient C–C coupling is associated with the buildup of a near-surface CO reservoir, with an effective local concentration estimated to far exceed the bulk solubility limit of CO in water.^51–53^

Homogeneous catalysis can generate high local CO concentration. Diffusion-ordered NMR spectroscopy (DOSY) yields a diffusion coefficient of (1.88 ± 0.03) × 10^–6^ cm^2^ s^–1^ for 0.5 mM Fe-*p*-TMA in D_2_O (Figure S25). In the absence of a chemical reaction, the reduced catalyst would diffuse across the entire diffusion layer. However, because CO_2_ reacts with Fe^0^ before the porphyrin diffuses into the bulk, the Fe^0^ is consumed within a thin reaction-diffusion layer, confining CO formation to the immediate vicinity of the electrode and raising the local CO concentration there.^56^ The rate of the chemical step determines the thickness of this layer.^56^ Fe-*p*-TMA is known for its selective and efficient CO_2_-to-CO reactivity.^2^ Scan-rate-dependent cyclic voltammetry of 0.5 mM Fe-*p*-TMA in 0.5 M KHCO_3_ under CO_2_ shows a substantially larger current than under Ar and exhibits a linear dependence of Fe^I/0^ peak current on the square root of scan rate, consistent with a diffusion-limited reaction (Figure S26). Although the background hydrogen evolution reaction precludes extraction of a quantitative rate constant, the catalysis regime can be determined by comparing the measured CO partial current density with that expected if the catalytic process were limited by diffusion of the catalyst across the diffusion layer. With stirring at 1000 rpm (Figure 6), the measured CO partial current density exceeds that expected for the diffusion-limited case by 2 orders of magnitude (Table S4). The system is therefore expected to operate in the fast catalysis regime, in which catalysis occurs only within a thin reaction-diffusion layer and the bulk is essentially unaffected,^56^ producing an effective buildup of the local CO concentration at the electrode. The estimated reaction-diffusion layer thickness is μ = 0.34–0.82 μm (Table S4).

Several observations indicate, however, that this cannot be the complete explanation. The central experimental observation is that dissolved CO at bulk saturation (∼1 mM) produces no detectable hydrocarbons on the electrode under flow conditions, whereas the same electrode produces C_1_–C_4_ products in the presence of Fe-*p*-TMA (Figure 1). Moreover, higher faradaic efficiencies for C_1_–C_4_ products are obtained under conditions in which the estimated local CO concentration is below 1 mM. At 0.05 mM Fe-*p*-TMA with stirring at 1000 rpm, the partial current density for CO is 0.11 mA cm^–2^ (Figure 6). Even assuming the reaction-diffusion layer to be negligibly thin and all CO formation to occur at the electrode, the local CO concentration estimated from planar linear diffusion is ∼0.26 mM (Table S4), yet the total hydrocarbon faradaic efficiency reaches 2.7%. The reported literature for electrochemical Fischer–Tropsch under 30 atm likewise shows no direct correlation between the amount of CO formed and the C_1_–C_4_ yield (Table S3).^39,40,44^ Therefore, although fast catalysis by the homogeneous porphyrin is essential for building up the local CO concentration, a complementary explanation beyond CO_2_-to-CO conversion is required.

### CO Shuttle Mechanism: Electrochemically Gated Delivery

We propose that Fe-*p*-TMA functions as a reversible CO shuttle that delivers CO directly to the electrode surface through electrochemically gated release (Scheme 3b). In its established catalytic role, the reduced Fe^0^ state converts CO_2_ to CO in the reaction-diffusion layer (**step i**). Outside the compact layer, Fe^II^–CO is formed either directly during the CO_2_-to-CO catalytic cycle or by Fe^II^ species binding dissolved CO from bulk solution (**step ii**). The Fe^II^–CO adduct then approaches the compact layer and undergoes further reduction at the electrode. Upon reduction to Fe^I^ or Fe^0^, CO is released in direct proximity to the electrode surface, where it can adsorb and participate in surface-mediated reactions (**step iii**). The reduced iron species is then regenerated as Fe^II^–CO outside the compact layer, completing the CO-shuttling cycle. In this way, Fe-*p*-TMA facilitates the transport of CO through the ion-dense, hydrogen-evolution dominated region directly to the electrode surface. The strong binding affinity and stability of the Fe^II^–CO thus increase the local CO activity within the compact layer and the extent of direct CO adsorption at the surface, producing an effect analogous to that obtained at 30 atm CO_2_.

This mechanism requires that CO is released at the electrode surface rather than in bulk solution. Although Fe^II^–CO could in principle be reduced through comproportionation with Fe^0^ in the reaction-diffusion layer, direct electrochemical reduction at the electrode surface is likely favored under these conditions. Comproportionation requires bimolecular encounter between Fe^II^–CO and Fe^0^, but any Fe^0^ formed is expected to react preferentially with the large excess of CO_2_ (bulk concentration is ∼34 mM,^57^ compared with 0.5 mM for the porphyrin), particularly given the rapid kinetics of CO_2_ binding to Fe^0^ porphyrins. Moreover, Fe^I^ is identified as the resting state under catalytic conditions,^26^ indicating that the steady-state concentration of Fe^0^ remains low and thereby further reducing the probability of encounter with Fe^II^–CO before Fe^0^ is consumed. These considerations indicate that direct reduction of Fe^II^–CO at the electrode surface is the favored pathway under the conditions employed here, favoring CO release within the compact layer.

The key mechanistic feature enabling localized CO delivery is the stability of Fe^II^–CO at potentials positive of the Fe^II/I^ couple. Cyclic voltammetry and spectroelectrochemical measurements under Ar and CO show that CO remains bound to the iron center across the entire potential range between open circuit and the Fe^II/I^ couple (Figures 7, S18, S20). Photochemical generation of Fe^II^–CO further indicates that this species persists for at least 1 h at open-circuit potential in aqueous electrolyte under CO atmosphere (Figure 8). Under catalytic conditions in CO_2_-saturated electrolyte, stepping from −0.9 to −0.2 V vs RHE results in spontaneous formation of stable Fe^II^–CO from CO_2_-derived CO (Figure 9), supporting the formation and persistence of this species under the less reducing conditions expected further from the electrode surface under catalytic conditions. CO dissociation is predominantly triggered by reduction of Fe^II^–CO to Fe^I^ or Fe^0^, a process that occurs only in close proximity to the electrode surface within the compact layer. This localization allows each catalytic turnover to function as a discrete CO delivery event near the electrode. The free-base porphyrin H_2_-*p*-TMA, which has no capacity for CO binding, does not produce products beyond CO, ruling out nonspecific transport or steric effects (Figure 1). The anionic analogue Fe-*p*-PSULF also produces beyond-CO products under otherwise identical conditions (Figure S7), indicating that positive ligand charge is not a prerequisite for beyond-CO product formation. Cyclic voltammetry shows that Fe-*p*-PSULF exhibits a smaller catalytic response under CO_2_ than Fe-*p*-TMA, consistent with its lower faradaic efficiency for CO. Therefore, the lower faradaic efficiencies of beyond-CO products with Fe-*p*-PSULF cannot be attributed solely to ligand charge, as differences in CO_2_-to-CO activity may also contribute. A systematic comparison across porphyrin derivatives would be required to isolate the effect of ligand charge.

The shuttle mechanism proposed here does not require Fe^II^–CO to be generated exclusively through the Fe^0^-mediated pathway. Rather, it requires that Fe^II^–CO form under conditions less reducing than those at the electrode, remain CO-bound under these conditions, and release CO upon further reduction. The recently reported Fe^I^-mediated pathway is consistent with these requirements: Fe^II^–CO generated from CO_2_ at a potential assigned to the Fe^I^ state remained bound, while further cathodic reduction was required for CO dissociation and catalytic turnover.^47^ Thus, if this pathway also operates under our conditions, it would provide an additional route to formation of the same Fe^II^–CO shuttle intermediate without altering the proposed mechanism of electrochemically gated CO release.

The buildup of local CO concentration through fast catalysis and the CO-shuttling effect are complementary. Under CO flow in the absence of Fe-*p*-TMA, the electrolyte is saturated with CO (∼1 mM), yet no beyond-CO products are formed. Adding Fe-*p*-TMA under the same CO flow leads to the formation of beyond-CO products, even though CO_2_-to-CO catalysis does not contribute. Under CO_2_ flow, both processes operate together: fast catalysis raises the CO concentration in the reaction-diffusion layer, while shuttling delivers CO into the compact layer with a higher flux. This accounts for the higher beyond-CO faradaic efficiencies obtained under CO_2_ flow than under CO flow. The higher faradaic efficiencies under CO_2_ flow than under CO flow were also reported for immobilized FePc systems and were attributed to the CO_2_-to-CO catalysis.^19^

Increasing the Fe-*p*-TMA concentration led to a steady increase in CO formation, whereas beyond-CO products exhibited a maximum, at 0.25 mM in unstirred conditions and 0.1 mM with stirring, before decreasing at higher concentrations (Figures 4 and 6). This behavior indicates that although increasing Fe-*p*-TMA concentration enhances CO_2_-to-CO conversion and CO shuttling, excessive local CO concentrations may become detrimental to downstream surface reactions by limiting the proton availability at the electrode surface. Prior studies on Cu electrode systems have shown that elevated local CO concentration increases the interfacial CO coverage and suppresses the competing hydrogen evolution reaction.^51,53,58^ In the present system, there is likely an optimal balance between local CO activity and proton supply for C–C coupling and chain growth, consistent with this balance being reached at lower catalyst concentration when mass transport is improved. These results therefore suggest the existence of an optimal CO-shuttle concentration that maximizes multicarbon product formation, likely reflecting the interplay between CO delivery, proton availability, and surface reaction kinetics.

### Implications for Molecular Catalyst Systems

The present findings may help rationalize prior reports regarding product formation in Fe-*p*-TMA-mediated CO_2_ reduction. In one report, trace CH_4_ formation was observed in aqueous electrolyte,^29^ whereas another study observed selective CO formation in organic solvent and attributed trace methane formation in DMF with ethanol as the proton source to catalyst decomposition.^26^ One possible origin of these differences is proton availability. In aprotic media such as DMF, proton donors including water and ethanol are substantially weaker than in aqueous electrolyte,^59,60^ leading to much lower effective proton availability under catalytic conditions. Under such conditions, surface-mediated hydrocarbon formation is likely suppressed even if local CO delivery occurs. In aqueous electrolyte, the greater availability of protons can permit hydrocarbon formation once sufficient local CO activity is established. From this perspective, prior reports of CH_4_ in aqueous electrolyte are consistent with a surface-mediated process promoted by CO shuttling rather than direct molecular reduction beyond CO. These results remain consistent with the conclusion that Fe-*p*-TMA itself does not reduce CO_2_ beyond CO.

This mechanistic framework may also help contextualize recent reports on heterogenized FePc systems, in which multicarbon products including hydrocarbons up to C_5_ have been observed.^19^ The present results demonstrate that comparable product yields can be achieved by using a homogeneous catalyst as a CO shuttle to an unmodified carbon electrode, where further reduction beyond CO occurs at the electrode surface. This observation suggests that surface-mediated pathways may also contribute to the product distributions reported for heterogenized FePc systems. In that context, the molecular catalyst may function primarily as a local CO generator in the compact layer, increasing local CO activity, rather than serving as the sole site of further reduction. A similar concept, in which heterogenized FeTPP functions as a local CO generator on a Cu metal electrode to enhance ethanol formation, has been reported.^58^ Whether analogous electrode-mediated contributions similarly affect other heterogenized molecular catalyst systems requires further investigation.

The use of triethanolamine as a CO_2_ shuttle for homogeneous electrocatalysis has recently been reported.^61^ Triethanolamine binds CO_2_ as a zwitterionic alkylcarbonate and delivers it to a molecular FeTPP catalyst, enhancing homogeneous CO_2_-to-CO conversion in organic solvent. Although our approach differs in using homogeneous porphyrin as an electrochemically gated shuttle that delivers CO to a heterogeneous electrode for CO reduction, both illustrate that molecular shuttling is a general strategy for enabling catalytic reactivity that is otherwise inaccessible.

The present system is intended as a mechanistic model. Gradual degradation of Fe-*p*-TMA under strongly reducing conditions limits the operational lifetime. The identification of alternative CO shuttle molecules with enhanced stability at the potentials required for surface catalysis is therefore critical for future development. The microscopic identity of the electrode-surface sites that engage shuttle-released CO remains unresolved, and *in situ* surface-sensitive spectroscopic methods together with computational modeling will be valuable for characterizing adsorption and dynamics at the electrode interface. Nonetheless, the present results support a model in which electrochemically gated CO delivery by a molecular shuttle promotes Fischer–Tropsch-type chemistry on an electrode that does not otherwise produce multicarbon products. This concept of molecular catalysts functioning as electrochemically gated intermediate shuttles may extend beyond CO to other reaction intermediates with reversible binding chemistry.

## Conclusion

We have demonstrated that a homogeneous, water-soluble iron porphyrin (Fe-*p*-TMA) enables the formation of isotopically verified C_1_–C_4_ hydrocarbons from CO_2_ on a carbon electrode in aqueous electrolyte. These products are not observed in the absence of the catalyst, even in CO-saturated bulk solvent. We propose that these products arise from electrochemical Fischer–Tropsch chemistry at the electrode surface, enabled by Fe-*p*-TMA acting as an electrochemically gated CO shuttle. Fe^II^–CO forms and remains stable under the less reducing conditions of the diffusion layer, while further reduction near the electrode surface favors CO release within the compact layer, enhancing local CO activity and promoting further surface reactivity. More broadly, this mechanism suggests a distinct role for molecular catalysts in electrochemical CO_2_ reduction: they can function not only as sites of catalytic turnover but also as shuttles that deliver key reaction intermediates directly to the electrode surface through localized, electrochemically triggered release. These findings offer an alternative framework for interpreting beyond-CO products observed in heterogenized molecular catalyst systems and suggest molecular intermediate shuttling as a design principle that may extend beyond CO to other reversibly bound reaction intermediates.

## Supplementary Material



## Notes

Experimental details, additional controlled-potential electrolysis data, ^1^H NMR spectra, isotopic labeling mass spectra, UV–vis spectroelectrochemistry, DOSY analysis, SEM-EDS, XPS, and scan-rate-dependent cyclic voltammetry (PDF)

## References

[1] Shin H., Hansen K. U., Jiao F. (2021). Techno-Economic Assessment of Low-Temperature
Carbon Dioxide Electrolysis. Nat. Sustainable.

[2] Costentin C., Robert M., Savéant J.-M., Tatin A. (2015). Efficient and Selective
Molecular Catalyst for the CO_2_-to-CO Electrochemical Conversion
in Water. Proc. Natl. Acad. Sci. U. S. A.

[3] Costentin C., Robert M., Savéant J.-M. (2013). Catalysis of the Electrochemical
Reduction of Carbon Dioxide. Chem. Soc. Rev.

[4] Zhang S., Fan Q., Xia R., Meyer T. J. (2020). CO_2_ Reduction: From Homogeneous
to Heterogeneous Electrocatalysis. Acc. Chem.
Res.

[5] Boutin E., Merakeb L., Ma B., Boudy B., Wang M., Bonin J., Anxolabéhère-Mallart E., Robert M. (2020). Molecular Catalysis of CO_2_ Reduction: Recent
Advances and Perspectives in Electrochemical and Light-Driven Processes
with Selected Fe, Ni and Co Aza Macrocyclic and Polypyridine Complexes. Chem. Soc. Rev.

[6] Azcarate I., Costentin C., Robert M., Savéant J.-M. (2016). Through-Space
Charge Interaction Substituent Effects in Molecular Catalysis Leading
to the Design of the Most Efficient Catalyst of CO_2_-to-CO
Electrochemical Conversion. J. Am. Chem. Soc.

[7] Nitopi S., Bertheussen E., Scott S. B., Liu X., Engstfeld A. K., Horch S., Seger B., Stephens I. E. L., Chan K., Hahn C., Nørskov J. K., Jaramillo T. F., Chorkendorff I. (2019). Progress and Perspectives of Electrochemical CO_2_ Reduction on Copper in Aqueous Electrolyte. Chem. Rev.

[8] Costentin C., Savéant J.-M. (2014). Multistep
Molecular Catalysis of Electrochemical Reactions:
Benchmarking of Homogeneous Catalysts. ChemElectrochem.

[9] Saha P., Amanullah S., Barman S., Dey A. (2025). Electrochemical Reduction
of CO_2_ to CH_3_OH Catalyzed by an Iron Porphyrinoid. J. Am. Chem. Soc.

[10] Patra S., Bhunia S., Ghosh S., Dey A. (2024). Outer-Coordination-Sphere
Interaction in a Molecular Iron Catalyst Allows Selective Methane
Production from Carbon Monoxide. ACS Catal.

[11] Patra S., Dinda S., Ghosh S., Roy T., Dey A. (2025). Synthesis
of Ethane from CO_2_ by a Methyl Transferase–Inspired
Molecular Catalyst. Proc. Natl. Acad. Sci. U.
S. A.

[12] Liang H.-Q., Beweries T., Francke R., Beller M. (2022). Molecular Catalysts
for the Reductive Homocoupling of CO_2_ towards C_2+_ Compounds. Angew. Chem., Int. Ed.

[13] Wu Y., Jiang Z., Lu X., Liang Y., Wang H. (2019). Domino Electroreduction
of CO_2_ to Methanol on a Molecular Catalyst. Nature.

[14] Boutin E., Wang M., Lin J. C., Mesnage M., Mendoza D., Lassalle-Kaiser B., Hahn C., Jaramillo T. F., Robert M. (2019). Aqueous Electrochemical
Reduction of Carbon Dioxide
and Carbon Monoxide into Methanol with Cobalt Phthalocyanine. Angew. Chem., Int. Ed.

[15] Weng Z., Jiang J., Wu Y., Wu Z., Guo X., Materna K. L., Liu W., Batista V. S., Brudvig G. W., Wang H. (2016). Electrochemical CO_2_ Reduction
to Hydrocarbons on a Heterogeneous
Molecular Cu Catalyst in Aqueous Solution. J.
Am. Chem. Soc.

[16] Weng Z., Wu Y., Wang M., Jiang J., Yang K., Huo S., Wang X.-F., Ma Q., Brudvig G. W., Batista V. S. (2018). Active Sites of Copper-Complex
Catalytic Materials for Electrochemical
Carbon Dioxide Reduction. Nat. Commun.

[17] Gonglach S., Paul S., Haas M., Pillwein F., Sreejith S. S., Barman S., De R., Müllegger S., Gerschel P., Apfel U.-P. (2019). Molecular
Cobalt Corrole
Complex for the Heterogeneous Electrocatalytic Reduction of Carbon
Dioxide. Nat. Commun.

[18] De R., Gonglach S., Paul S., Haas M., Sreejith S. S., Gerschel P., Apfel U.-P., Vuong T. H., Rabeah J., Roy S., Schöfberger W. (2020). Electrocatalytic Reduction of CO_2_ to Acetic Acid by a Molecular Manganese Corrole Complex. Angew. Chem., Int. Ed.

[19] Dong S.-T., Xu C., Lassalle-Kaiser B. (2023). Multiple C–C
Bond Formation
upon Electrocatalytic Reduction of CO_2_ by an Iron-Based
Molecular Macrocycle. Chem. Sci.

[20] Abdinejad M., Farzi A., Möller-Gulland R., Mulder F., Liu C., Shao J., Biemolt J., Robert M., Seifitokaldani A., Burdyny T. (2024). Eliminating redox-mediated
electron transfer mechanisms
on a supported molecular catalyst enables CO_2_ conversion
to ethanol. Nat. Catal.

[21] Margarit C. G., Asimow N. G., Costentin C., Nocera D. G. (2020). Tertiary Amine-Assisted
Electroreduction of Carbon Dioxide to Formate Catalyzed by Iron Tetraphenylporphyrin. ACS Energy Lett.

[22] Mondal B., Rana A., Sen P., Dey A. (2015). Intermediates Involved
in the 2e^–^/2H^+^ Reduction of CO_2_ to CO by Iron(0) Porphyrin. J. Am. Chem. Soc.

[23] Li X., Chai G., Xu X., Liu J., Zhong Z., Cao A., Tao Z., You W., Kang L. (2020). Electrocatalytic Reduction
of CO_2_ to CO over Iron Phthalocyanine-Modified Graphene
Nanocomposites. Carbon.

[24] Peel-Smith D., Durfy C. S., Nichols E. M. (2026). CO Binding
at Iron Porphyrins: Reflecting
on Heme Model Complexes to Guide Future Electrocatalyst Design. J. Inorg. Biochem.

[25] Teindl K., Nichols E. M. (2026). The Effect of Exogenous Acid Identity on Iron Tetraphenylporphyrin-Catalyzed
CO_2_ Reduction. Inorg. Chem.

[26] Salamé A., Cheah M. H., Bonin J., Robert M., Anxolabéhère-Mallart E. (2024). Operando Spectroelectrochemistry
Unravels the Mechanism of CO_2_ Electrocatalytic Reduction
by an Fe Porphyrin. Angew. Chem., Int. Ed.

[27] Rao H., Schmidt L. C., Bonin J., Robert M. (2017). Visible-Light-Driven
Methane Formation from CO_2_ with a Molecular Iron Catalyst. Nature.

[28] Rao H., Lim C., Bonin J., Miyake G. M., Robert M. (2018). Visible-Light-Driven
Conversion of CO_2_ to CH_4_ with an Organic Sensitizer
and an Iron Porphyrin Catalyst. J. Am. Chem.
Soc.

[29] Li Y., Xie S., Huang X., Song W., Chen C., Sheng H., Zhao J. (2023). Spectating the Proton Migration on Catalyst with Noninnocent Ligand
in Aqueous Electrochemical CO_2_ Reduction. Appl. Catal., B.

[30] Wu Y., Hu G., Rooney C. L., Brudvig G. W., Wang H. (2020). Heterogeneous Nature
of Electrocatalytic CO/CO_2_ Reduction by Cobalt Phthalocyanines. ChemSuschem.

[31] Hwang S. Y., Maeng J. Y., Yoon I., Myung C. W., Rhee C. K., Sohn Y. (2024). Electrochemical Fischer–Tropsch
Chemistry across Transition
Metals: A Paradigm Shift in Sustainable Liquid Fuel Production. Nano Energy.

[32] Dhanasekaran T., Grodkowski J., Neta P., Hambright P., Fujita E. (1999). P-Terphenyl-Sensitized Photoreduction of CO_2_ with Cobalt and Iron Porphyrins. Interaction between CO and Reduced
Metalloporphyrins. J. Phys. Chem. A.

[33] Marianov A.
N., Kochubei A. S., Roman T., Conquest O. J., Stampfl C., Jiang Y. (2021). Resolving
Deactivation Pathways of Co Porphyrin-Based Electrocatalysts
for CO_2_ Reduction in Aqueous Medium. ACS Catal.

[34] Zhang B. A., Ozel T., Elias J. S., Costentin C., Nocera D. G. (2019). Interplay of Homogeneous Reactions, Mass Transport,
and Kinetics in Determining Selectivity of the Reduction of CO_2_ on Gold Electrodes. ACS Cent. Sci.

[35] de
Jesús Gálvez-Vázquez M., Grozovski V., Kovács N., Broekmann P., Vesztergom S. (2020). Full Model
for the Two-Step Polarization Curves of Hydrogen Evolution, Measured
on RDEs in Dilute Acid Solutions. J. Phys. Chem.
C.

[36] Grodkowski J., Behar D., Neta P., Hambright P. (1997). Iron Porphyrin-Catalyzed
Reduction of CO_2_. Photochemical and Radiation Chemical
Studies. J. Phys. Chem. A.

[37] Hendrickson D. N., Kinnaird M. G., Suslick K. S. (1987). Photochemistry
of (5,10,15,20-Tetraphenylporphyrinato)­Iron­(III)
Halide Complexes, Fe­(TPP)­(X). J. Am. Chem. Soc.

[38] Hwang S. Y., Yun G., Yeo B. S., Sohn Y. (2025). Electrochemical Fischer–Tropsch
Chemistry. Chem. Eng. J.

[39] Hara K., Kudo A., Sakata T. (1995). Electrochemical Reduction
of High
Pressure Carbon Dioxide on Fe Electrodes at Large Current Density. J. Electroanal. Chem.

[40] Hara K., Kudo A., Sakata T. (1997). Electrochemical
CO_2_ Reduction
on a Glassy Carbon Electrode under High Pressure. J. Electroanal. Chem.

[41] Vafaie M., Dorakhan R., Morteza
Najarian A., Teimouri Z., Pofelski A., Barati N., Chou C.-H., Chang Y.-C., Hung S.-F., Sun Q., Azimi Dijvejin Z., Ngunjiri R., Li Y., Shayesteh
Zeraati A., Salem K. E., Miao R. K., Hoogland S., Higgins D., Sargent E. H., Sinton D. (2025). Direct Electrosynthesis
of C_3+_ Hydrocarbons from CO_2_ via Size-Controlled
Nickel Nanoislands on a Carbon Support. J. Am.
Chem. Soc.

[42] Preikschas P., Zhang J., Seemakurthi R. R., Lian Z., Martín A. J., Xi S., Krumeich F., Ma H., Zhou Y., López N. (2024). CO_2_ Electroreduction to Long-Chain Hydrocarbons on Cobalt
Catalysts. Adv. Energy Mater.

[43] Ma X., Yeo B. S. (2024). Electrocatalytic Reduction of Carbon Dioxide to C_4+_ Products. Curr. Opin. Electrochem.

[44] Nakagawa S., Kudo A., Azuma M., Sakata T. (1991). Effect of Pressure
on the Electrochemical Reduction of CO2 on Group VIII Metal Electrodes. J. Electroanal. Chem.

[45] Kluge R. M., Haid R. W., Stephens I. E. L., Calle-Vallejo F., Bandarenka A. S. (2021). Monitoring
the Active Sites for the Hydrogen Evolution
Reaction at Model Carbon Surfaces. Phys. Chem.
Chem. Phys.

[46] Wang L., Sofer Z., Pumera M. (2019). Catalytic Hydrogen Evolution Reaction
on “Metal-Free” Graphene: Key Role of Metallic Impurities. Nanoscale.

[47] Howe A., Salamé A., Garnier U., Robert M., Anxolabéhère-Mallart E., Cheah M. H. (2026). CO_2_-to-CO Electrochemical Conversion with
an Fe­(I) Porphyrin Complex in Water. Angew.
Chem., Int. Ed.

[48] Zhu Q., Rooney C. L., Shema H., Zeng C., Panetier J. A., Gross E., Wang H., Baker L. R. (2024). The Solvation Environment
of Molecularly Dispersed Cobalt Phthalocyanine Determines Methanol
Selectivity during Electrocatalytic CO2 Reduction. Nat. Catal.

[49] Savéant J.-M. (2008). Molecular
Catalysis of Electrochemical Reactions. Mechanistic Aspects. Chem. Rev.

[50] Hicks M. H., Nie W., Boehme A. E., Atwater H. A., Agapie T., Peters J. C. (2024). Electrochemical
CO_2_ Reduction in Acidic Electrolytes: Spectroscopic Evidence
for Local pH Gradients. J. Am. Chem. Soc.

[51] Park S., Grigioni I., Alkayyali T., Lee B.-H., Kim J., Shirzadi E., Dorakhan R., Lee G., Abed J., Bossola F., Jung E. D., Liang Y., Lee M. G., Shayesteh Zeraati A., Kim D., Sinton D., Sargent E. (2023). High Carbon
Efficiency in CO-to-Alcohol Electroreduction Using a CO Reservoir. Joule.

[52] Louisia S., Kim D., Li Y., Gao M., Yu S., Roh I., Yang P. (2022). The Presence and Role of the Intermediary
CO Reservoir in Heterogeneous
Electroreduction of CO2. Proc. Natl. Acad. Sci.
U. S. A.

[53] He A., Yang Y., Zhang Q., Yang M., Zou Q., Du J., Tao C., Liu Z. (2022). The Enhanced Local CO Concentration
for Efficient CO_2_ Electrolysis towards C2 Products on Tandem
Active Sites. Chem. Eng. J.

[54] Zhang G., Schreier M. (2024). Leveraging electrochemical double
layer structure to
rationally control electrolysis. Natl. Sci.
Rev.

[55] Fuller L., Zhang G., Noh S., Van Lehn R. C., Schreier M. (2025). Electrolyte
Anions Suppress Hydrogen Generation in Electrochemical CO Reduction
on Cu. Angew. Chem., Int. Ed.

[56] Deeba R., Collard A., Rollin C., Molton F., Chardon-Noblat S., Costentin C. (2023). Controlled Potential Electrolysis:
Transition from
Fast to Slow Regimes in Homogeneous Molecular Catalysis. Application
to the Electroreduction of CO2 Catalyzed by Iron Porphyrin. ChemElectrochem.

[57] Dodds W. S., Stutzman L. F., Sollami B. J. (1956). Carbon Dioxide Solubility in Water. Ind. Eng. Chem. Chem. Eng. Data Ser.

[58] Li F., Li Y. C., Wang Z., Li J., Nam D.-H., Lum Y., Luo M., Wang X., Ozden A., Hung S.-F., Chen B., Wang Y., Wicks J., Xu Y., Li Y., Gabardo C. M., Dinh C.-T., Wang Y., Zhuang T.-T., Sinton D., Sargent E. H. (2020). Cooperative CO_2_-to-Ethanol
Conversion via Enriched Intermediates at Molecule–Metal Catalyst
Interfaces. Nat. Catal.

[59] Olmstead W. N., Margolin Z., Bordwell F. G. (1980). Acidities
of Water and Simple Alcohols
in Dimethyl Sulfoxide Solution. J. Org. Chem.

[60] Heller S. T., Silverstein T. P. (2020). pKa Values in the Undergraduate Curriculum: Introducing
pKa Values Measured in DMSO to Illustrate Solvent Effects. ChemTexts.

[61] Yin Z., Zhang M., Long Y., Lei H., Li X., Zhang X.-P., Zhang W., Apfel U.-P., Cao R. (2025). Improving
Electrocatalytic CO_2_ Reduction over Iron Tetraphenylporphyrin
with Triethanolamine as a CO_2_ Shuttle. Angew. Chem., Int. Ed.

