# Supplementary material for: Homogeneous Iron Porphyrin as a Molecular CO Shuttle Enables Electrochemical Multicarbon Product Formation from CO2
Source: J Am Chem Soc. 2026 Sep 3;148(36):38891–903. doi: 10.1021/jacs.6c10179 (PMC13587666; doi:10.1021/jacs.6c10179)
Supplement: Supplementary file 1 [file ja6c10179_si_001.pdf]

Supporting Information for

Homogeneous Iron Porphyrin as a Molecular CO Shuttle Enables Electrochemical Multicarbon Product Formation from CO_2

Keon Ha Hwang, Stephen P. DiLuzio, Kaitlin M. Luedecke, Ryan G. Hadt*

Division of Chemistry and Chemical Engineering, Arthur Amos Noyes Laboratory of Chemical Physics, California Institute of Technology, Pasadena, California 91125, United States

*Corresponding Author: rghadt@caltech.edu

Table of Contents

I. Experimental..1

1. Materials
2. Synthesis of Fe-*p*-TMA
3. Controlled Potential Electrolysis
4. Product Analysis
5. Electrochemical and Spectroscopic Characterization

II. Supplementary Figures & Tables 7

1. Controlled Potential Electrolysis Results
2. Rinse Test Method Result
3. Isotopic Labeling GC-MS Results
4. Concentration Dependence Results
5. Catalyst Stability Test Result
6. Electrode Characterization before and after Electrolysis
7. Estimation of Diffusion Layer Thickness
8. Spectroelectrochemistry and Cyclic Voltammetry under Ar and CO
9. Stability of Fe^{II}–CO in Organic Solvent
10. Literature Summary of Electrochemical Fischer–Tropsch on Carbon and Iron Electrodes
11. CO-Induced Suppression of HER on Glassy Carbon Electrode
12. DOSY Measurements
13. Scan-Rate-Dependent Cyclic Voltammetry
14. Estimation of Reaction-Diffusion Layer Thickness and the Local CO Concentration at the Electrode

I. Experimental

1. Materials

All solvents and reagents were obtained from commercial sources and used as received, unless otherwise noted. 5,10,15,20-Tetra(4'-N,N,N-trimethylanilinium)porphyrin tetrachloride (H_2-*p*-TMA) and *meso*-tetra(4′-sulfonatophenyl) iron(III) porphyrin (Fe-*p*-PSULF) (acid form) were purchased from Frontier Specialty Chemicals. Mohr's salt ($Fe(NH_4)_2(SO_4)_2 \cdot 6H_2O$) was purchased from Fisher Scientific. Tetrabutylammonium hexafluorophosphate ($TBAPF_6$ >99%, for electrochemical analysis), K_2CO_3 (99.995%; trace metal basis), $^{13}CO_2$ (99%), and $NaH^{13}CO_3$ (99%) were purchased from Sigma-Aldrich. D_2O (D 99.96%) was purchased from Cambridge Isotope Laboratories. Platinum foil (99.99% Pt, 25 mm × 25 mm × 0.05 mm) was purchased from Alfa Aesar. Selemion AMV anion-exchange membranes were purchased from AGC Engineering Co. Plain carbon cloth (1071 HCB) was purchased from AvCarb Material Solutions. Leakless Ag/AgCl reference electrodes were purchased from eDaq. Aqueous Ag/AgCl (3 M KCl) reference electrodes were purchased from Sigma-Aldrich. Titanium electrode holders were purchased from StonyLab. Water was purified by a Nanopure Analytical Ultrapure Water System (Thermo Scientific) or a Milli-Q Advantage A10 Water Purification System (Millipore) with specific resistance of 18.2 MΩ·cm at 25 °C.

2. Synthesis of Fe-*p*-TMA

The following procedure was adapted from a previous report.[1] H_2-*p*-TMA (102 mg) and Mohr's salt (324 mg) were dissolved in 40 mL of ultrapure water. The mixture was stirred at 85 °C under N_2 for 3 h. The metalated porphyrin was precipitated by addition of NH_4PF_6 (155 mg), and the suspension was centrifuged at 3,000 rpm for 20 min. The solid was collected and washed with 25 mL ultrapure water containing NH_4PF_6 (155 mg) followed by sequential washing with 15 mL of acetone/CH_2Cl_2 (1:1), 5 mL of MeOH, and 5 × 5 mL of acetone/CH_2Cl_2 (1:1). The residue was dissolved in 98.5 mL of acetone and 2.5 mL of concentrated HCl (37% (aq)) was added dropwise to exchange the counterions. The mixture was sonicated for 10 min, centrifuged at 3,000 rpm for 20 min, and the solid was collected and washed with acetone (25 mL). The product was dissolved in minimal MeOH and precipitated with EtOAc, then redissolved in minimal MeOH and crystallized by vapor diffusion with Et_2O. After drying, Fe-*p*-TMA was obtained as a dark red-brown powder.

3. Controlled Potential Electrolysis

All electrochemical experiments were performed using a Biologic VMP3 multichannel potentiostat. The 0.5 M $KHCO_3$ electrolyte used in all experiments was prepared by sparging 0.25 M K_2CO_3 (aq) solution with CO_2 gas overnight to obtain high purity. Before each experiment, the electrolyte was sparged with CO_2 gas for at least 30 min. Ohmic resistances were measured by carrying out potentiostatic electrochemical impedance spectroscopy (PEIS) measurements from 100 Hz to 200 kHz prior to each electrolysis. The Ohmic resistance was determined from the real axis intercept of the linear fit of the high-frequency region of the Nyquist plot. Typical values of the resistance range from 5 to 15 Ω, and 85% Ohmic drop compensation was applied.

Controlled potential experiments under gas flow conditions were performed in a custom-made PEEK gas flow cell similar to that reported by Ager et al.[2] Carbon cloth was used as the working electrode and platinum foil was used as the counter electrode. The exposed carbon cloth in the catholyte was 1 cm^2. The cathode and anode compartments were separated by a Selemion AMV anion-exchange membrane. Potentials were measured against a leakless Ag/AgCl reference electrode inserted into the cathode

compartment and calibrated against a saturated calomel reference electrode. All experiments were performed for 30 min at room temperature using CO_2- or CO-saturated 0.5 M $KHCO_3$ electrolyte under 5 sccm CO_2 or CO flow. Because light cannot pass through the cell, any photochemical contribution can be excluded.

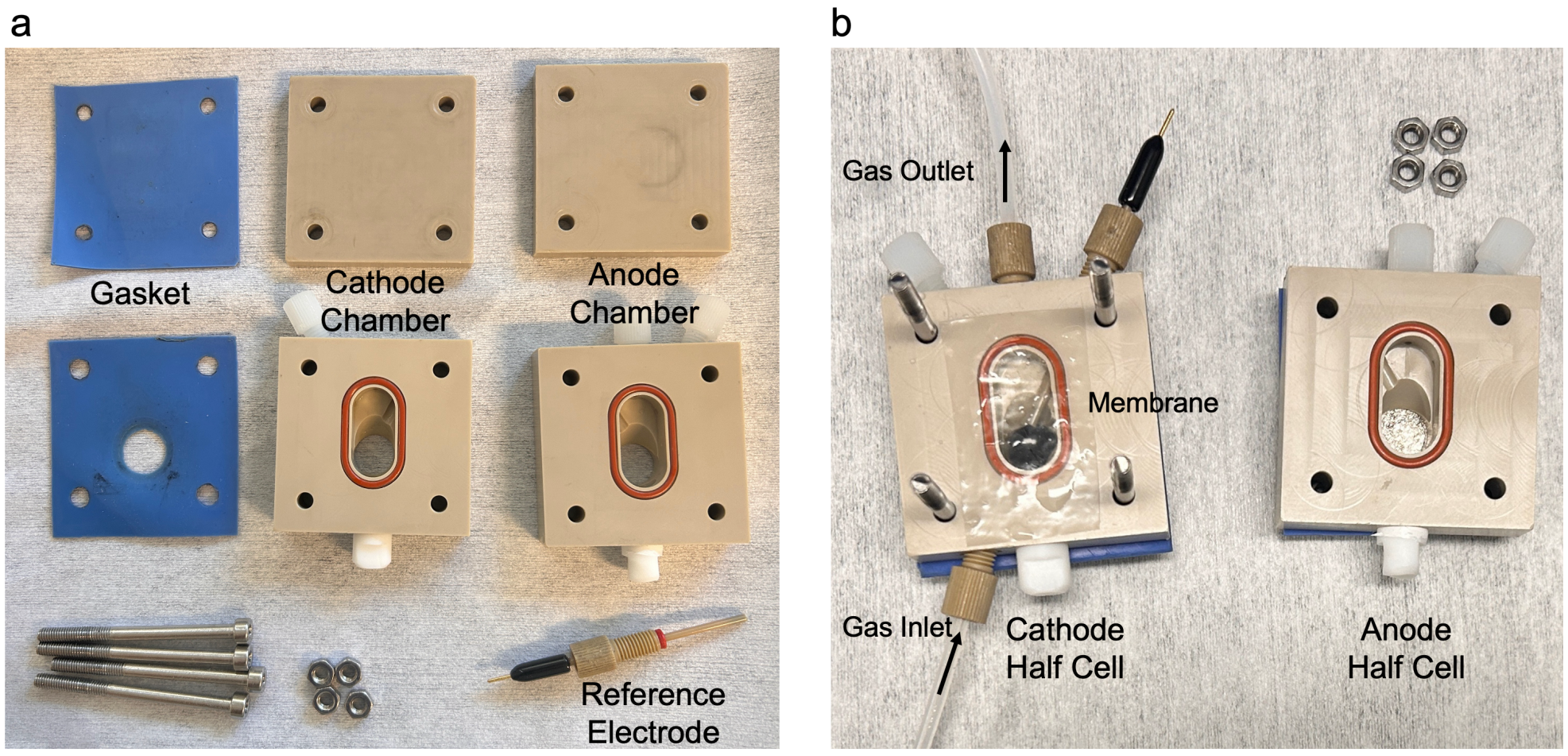

Figure S1. Photographs of the two-compartment PEEK electrochemical cell used for controlled potential electrolysis under gas flow conditions: (a) individual components before assembly and (b) the assembled cathode and anode half-cells.

Controlled potential experiments under batch conditions were performed in a custom-made glass H-cell. The working compartment (20 mL) and counter compartment (20 mL) were separated by a Selemion AMV anion-exchange membrane. 0.25 cm^2 carbon cloth was used as the working electrode and platinum mesh was used as a counter electrode, both held by titanium electrode holders. Potentials were measured against an aqueous Ag/AgCl (3 M KCl) reference electrode calibrated against a saturated calomel reference electrode. Both compartments were sealed using GL14 and GL18 closed caps or open-top caps with silicone/PTFE septa. Each side was filled with 6 mL of electrolyte and purged with CO_2 for 30 min before sealing. Electrolysis was performed for 30 min except for isotope-labeled experiments, which were performed for 90 min to improve quantification of carbon-labeled products. The fume-hood light was switched off to avoid any photochemical contribution.

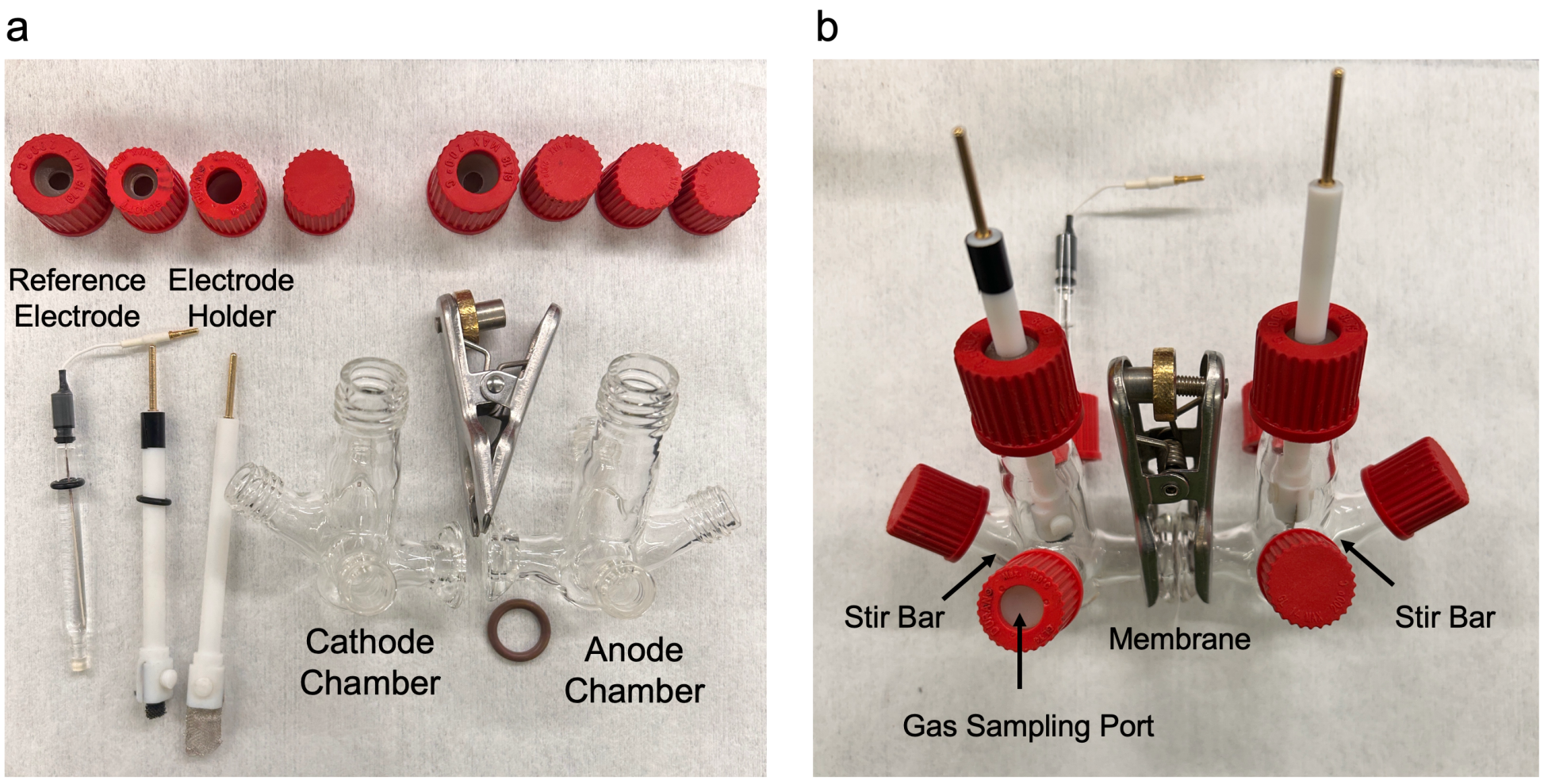

Figure S2. Photographs of the two-compartment glass electrochemical cell used for controlled potential electrolysis under batch conditions: (a) individual components before assembly and (b) the assembled cell.

4. Product Analysis

For gas flow conditions, gaseous products were quantified by gas chromatography (GC) using an SRI 8610C gas chromatograph equipped with a thermal conductivity detector (TCD) and a flame ionization detector with a methanizer (FID). Argon was used as the carrier gas at a pressure of 14 psi at a rate of 40 mL min^{-1}. The temperature protocol was: 50 °C for 1.75 min, ramp to 150 °C at 80 °C min^{-1}, then hold for 2.7 min. A 6 ft Haysep-D column was used for separation, and gas was sampled every 10 min. CO, CH_4, C_2H_4, and C_2H_6 were quantified from FID chromatograms, and H_2 was quantified from TCD chromatograms. Calibration curves were constructed using standard gas mixtures containing CO, CH_4, C_2H_4, C_2H_6, and H_2 at concentrations of 100 ppm, 500 ppm, 1000 ppm, and 5000 ppm in a CO_2 balance (Airgas). For CO flow experiments, calibration gases were prepared by diluting the standard gas mixtures with high-purity CO (Airgas) using Alicat Scientific MC-Series mass flow controllers. For gas flow conditions, faradaic efficiency was calculated as follows:

$$FE(\%) = \frac{n \times x \times F \times flow\ rate \times P}{R \times T \times I} \times 100$$

where n is the number of electrons required to form one molecule of product, x is the mol fraction of the gaseous product formed, F is the Faraday constant (96485 C mol^{-1}), flow rate is 5 sccm, P is ambient pressure (101325 Pa), R is the gas constant (8.314 J K^{-1} mol^{-1}), T is ambient temperature (298 K), and I is the average current during the sampling time.

For the batch conditions, GC-MS measurements were performed on an Agilent 7820A GC coupled to an Agilent 5977E quadrupole MS. 500 µL of headspace was injected via a gas-tight (Hamilton) syringe on a GS-CarbonPLOT in split mode (10:1 ratio, helium split flow of 8 mL min^{-1}) with a carrier gas flow of 0.8 mL min^{-1}. The inlet temperature was 150 °C. The temperature protocol was: 50 °C for 1.75 min, ramp to 200 °C at 80 °C min^{-1}, then hold for 15 min. The electron ionization source was operated at 70 eV. The transfer line, MS source, and quadrupole temperatures were 250, 230, and 150 °C, respectively. C_3–C_4 products were quantified using calibration gas mixtures of known C_1–C_4 composition, with C_2H_4 as an internal standard. C_1–C_2 products were quantified by direct injection of 200 µL of headspace gas into the SRI 8610C GC described above, calibrated with the same standard gas mixtures. For batch conditions, faradaic efficiency was calculated as follows:

$$FE(\%) = \frac{n \times x \times F \times V \times P}{R \times T \times Q} \times 100$$

where V is the headspace volume (14 mL), Q is the total charge passed, and all other variables are as defined above.

For liquid product analysis, ^{1}H NMR experiments were acquired on a Bruker 400 MHz NMR spectrometer equipped with a 5 mm broadband Smart Probe using water-suppression method. The relaxation delay was set to 30 s to ensure complete relaxation between measurements. 450 µL of the catholyte was mixed with 50 µL of 10 mM DMSO in D_2O, yielding a 1 mM DMSO internal standard referenced at 2.60 ppm. Formate was identified at 8.33 ppm and methanol at 3.23 ppm.

$$FE(\%) = \frac{N \times n \times F}{Q} \times 100$$

$$N(mol) = C_{DMSO} \times \frac{6}{n_H} \times V_{catholyte}$$

where N is the number of moles of liquid product formed, C_{DMSO} is 1 mM, n_H is the number of protons corresponding to the characteristic peak, $V_{catholyte}$ is the catholyte volume (1.7 mL under gas flow conditions), and all other variables are as defined above.

5. Electrochemical and Spectroscopic Characterization

Cyclic Voltammetry Cyclic voltammograms were recorded in a three-electrode configuration. The working electrode was a 3 mm diameter glassy carbon electrode polished with alumina slurry (0.05 μm) and rinsed with water and acetone before each measurement. The counter electrode was a platinum wire. In aqueous solvent, the reference electrode was Ag/AgCl (3 M KCl), calibrated against saturated calomel reference electrode. In organic solvent, the reference electrode was the Ag/$AgNO_3$ (0.1 M $TBAPF_6$/MeCN), and ferrocene was used as an internal standard; all potentials are reported versus $Fc^{+/0}$. Solutions were purged with the appropriate gas (Ar, CO_2, or CO) for at least 20 minutes before measurement. Ohmic drop was compensated at 85%.

UV–vis Absorption Spectroscopy UV–vis spectra were recorded at room temperature on a Cary 5000 spectrophotometer using 1 cm path length quartz cuvettes (Starna Cells, 6-Q) fitted with air-tight seals. All spectra were baseline-corrected against the solvent. Each measurement used 2 mL of solution at a Fe-*p*-TMA concentration of 20 μM. For photochemical generation of Fe^{II}–CO, samples were positioned 5 cm from a Kessil PR160L LED (λ = 390 nm) operated at maximum intensity. A cooling fan maintained room temperature during irradiation.

UV–vis Spectroelectrochemistry Spectra at controlled potentials were recorded at room temperature using a honeycomb spectroelectrochemistry cell (Pine Research) with a StellarNet Inc. Black Comet UV–vis spectrophotometer. The working electrode was a gold screen-printed honeycomb electrode with a 1.7 mm path length. In aqueous solvent, the reference electrode was a miniature Ag/AgCl reference calibrated against a saturated calomel reference electrode. In nonaqueous solvent, the reference electrode was a silver wire, with ferrocene as an internal standard; potentials are reported versus $Fc^{+/0}$. The counter electrode was a platinum wire. The Fe-*p*-TMA concentration was 0.1 mM.

DOSY Measurement All DOSY experiments were performed on a Bruker 400 MHz NMR spectrometer equipped with a 5 mm broadband iProbe using the dstebpgp3s pulse sequence (double stimulated echo for convection compensation and LED using bipolar gradient pulses) with a gradient pulse duration (P30) of 1000 μs. All data were processed using MestreNova software. The reported diffusion coefficient represents the average of three independent measurements.

X-ray Photoelectron Spectroscopy X-ray photoelectron spectroscopy (XPS) was performed using a Kratos Axis Ultra spectrometer (Kratos Analytical, Manchester, UK) with a base pressure of $<3 \times 10^{-9}$ Torr. The instrument was equipped with a hybrid magnetic and electrostatic electron lens system, a delay-line detector (DLD), and a monochromatic Al Kα X-ray source (1486.6 eV). The hemispherical analyzer was oriented for detection along the sample surface normal to maximize the depth sensitivity of the measurement. The instrument was operated using Vision Manager software v2.2.10 revision 5, and XPS data were analyzed using CasaXPS software v2.3.25PR1.0 (CASA Software Ltd.). The argon ion gun settings were 4 kV accelerating voltage. A 3 × 3 mm square area of each sample was etched with an argon ion gun for 5 min before measurement to improve signal. After electrolysis, the electrodes were gently washed with deionized water to remove residual electrolyte and dried before analysis.

Scanning Electron Microscopy Paired with Energy-Dispersive X-ray Spectroscopy (SEM-EDS)
SEM-EDS was performed using a JEOL JXA-iHP200F field-emission electron probe microanalyzer (EPMA) at an accelerating voltage of 15 kV. Elemental compositions were averaged over three different sites. After electrolysis, the electrodes were gently washed with deionized water to remove residual electrolyte and dried before analysis.

II. Supplementary Figures & Tables

1. Controlled Potential Electrolysis Results

Table S1. Faradaic efficiencies and current densities at −0.9 V vs. RHE using 0.5 mM Fe-*p*-TMA in 0.5 M $KHCO_3$ electrolyte under 5 sccm CO_2 and CO flow. Controls include 0.5 mM H_2-*p*-TMA condition and catalyst-free conditions.

Flow Gas	Catalyst	CO (FE %)	CH_4 (FE %)	C_2H_4 (FE %)	C_2H_6 (FE %)	H_2 (FE %)	Sum (FE %)	CD (mA/cm^2)
CO_2	Fe-*p*-TMA	23.45±7.98	1.68±0.30	0.31±0.07	0.50±0.12	56.94±8.74	82.90±1.08	4.51±1.16
	H_2-*p*-TMA	0.56±0.32	n.d.	n.d.	n.d.	96.53±3.90	97.11±3.82	3.27±0.47
	No-catalyst	0.42±0.33	n.d.	n.d.	n.d.	98.91±2.64	99.34±2.74	5.08±0.49
CO	Fe-*p*-TMA	balance	shoulder*	0.10±0.03	0.31±0.07	93.90±0.80	94.31±0.74	6.30±1.65
	No-catalyst	balance	n.d.	n.d.	n.d.	97.15±5.08	97.15±5.08	0.76±0.07

*CH_4 peak overlaps with the CO background in the GC chromatogram and could not be quantified independently.

*CD = current density; n.d. = not detected.

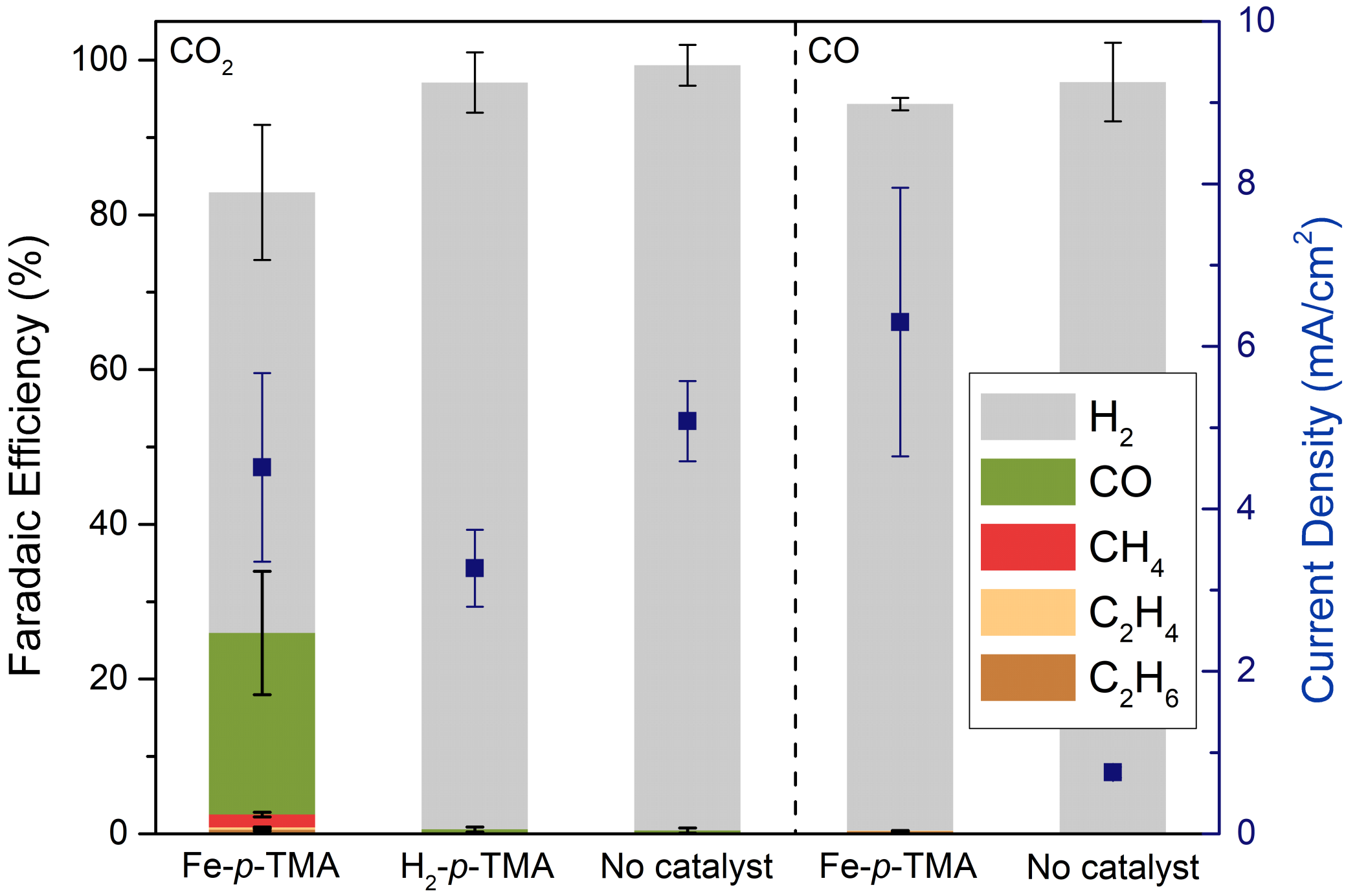

Figure S3. Faradaic efficiencies for all detected gas-phase products and current densities at −0.9 V vs. RHE using 0.5 mM Fe-*p*-TMA in 0.5 M $KHCO_3$ electrolyte under 5 sccm CO_2 (left) and CO (right) flow. Controls include 0.5 mM H_2-*p*-TMA condition and catalyst-free conditions. Error bars represent the standard deviation of three independent experiments.

Table S2. Faradaic efficiencies and current densities at −1.0 V vs. RHE using 0.5 mM Fe-*p*-TMA in 0.5 M $KHCO_3$ electrolyte under 5 sccm CO_2 and CO flow. Controls include 0.5 mM H_2-*p*-TMA condition and catalyst-free conditions.

Flow Gas	Catalyst	CO (FE %)	CH_4 (FE %)	C_2H_4 (FE %)	C_2H_6 (FE %)	H_2 (FE %)	Sum (FE %)	CD (mA/cm^2)
CO_2	Fe-*p*-TMA	3.30±0.80	1.08±0.30	0.20±0.05	0.46±0.18	86.11±2.83	91.13±2.38	14.23±2.69
	H_2-*p*-TMA	0.02±0.01	n.d.	n.d.	n.d.	98.35±0.70	98.41±0.69	8.44±0.54
	No-catalyst	0.05±0.04	n.d.	n.d.	n.d.	97.12±2.02	97.19±1.99	8.19±0.35

*CD = current density; n.d. = not detected.

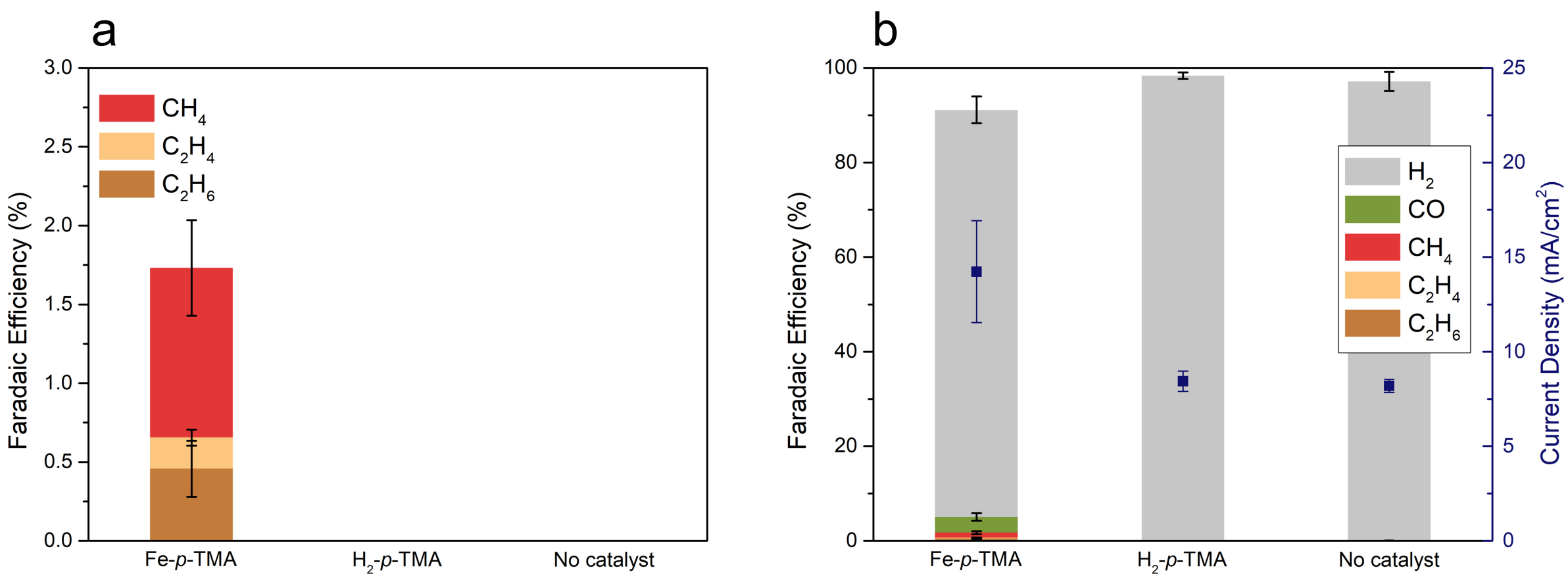

Figure S4. Faradaic efficiencies at −1.0 V vs. RHE using 0.5 mM Fe-*p*-TMA in 0.5 M $KHCO_3$ electrolyte under 5 sccm CO_2 flow for CH_4, C_2H_4, and C_2H_6 (a) and for all gaseous products with current density (b). Controls include 0.5 mM H_2-*p*-TMA condition and catalyst-free conditions. The faradaic efficiency for CO decreases substantially at −1.0 V vs. RHE compared to −0.9 V vs. RHE, consistent with dominant hydrogen evolution at more negative potentials, though the faradaic efficiency for beyond-CO products remains comparable. Error bars represent the standard deviation of three independent experiments.

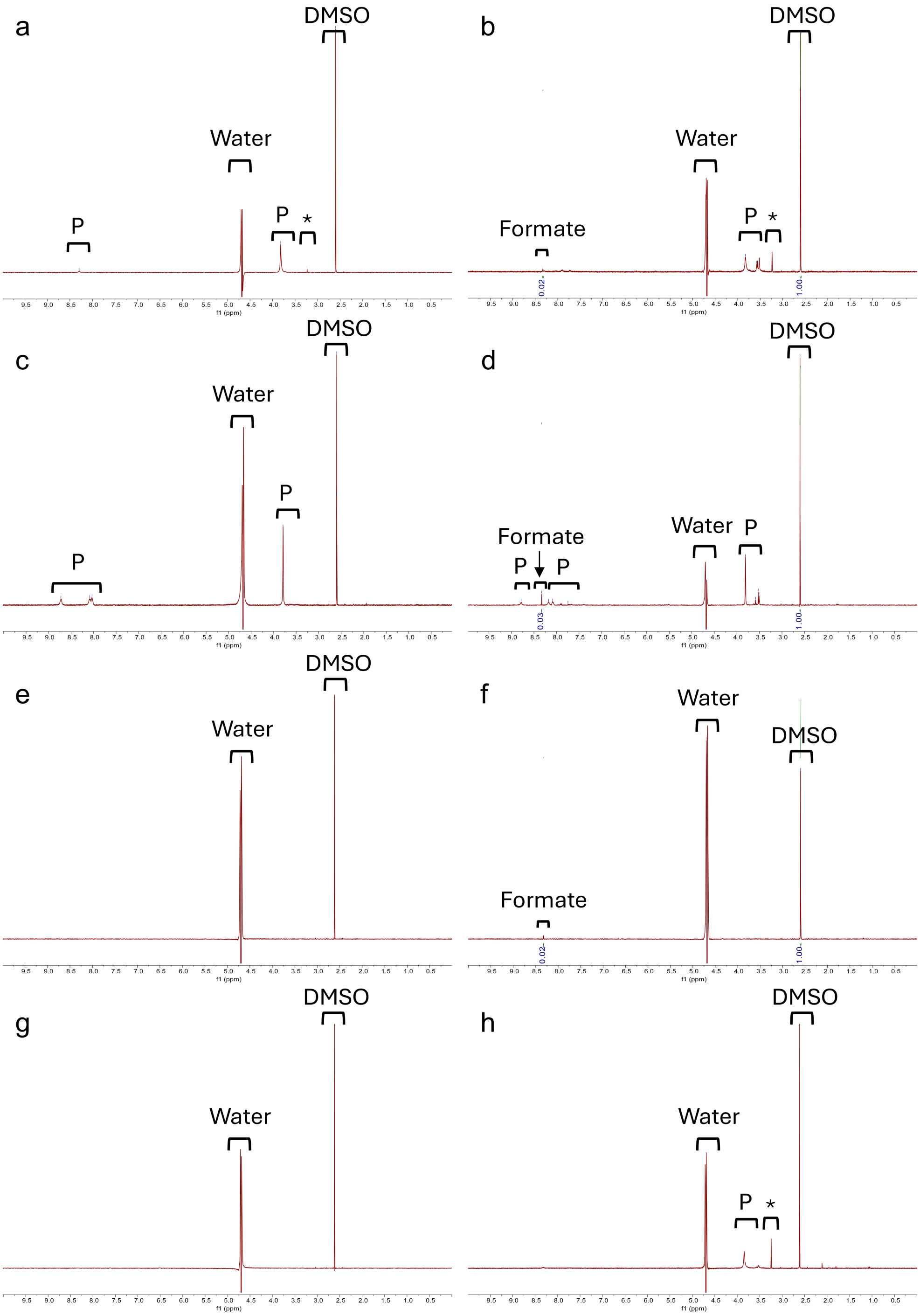

Figure S5. ^{1}H NMR spectra of catholyte before and after 30 min electrolysis at −0.9 V vs. RHE using 0.5 M $KHCO_3$ electrolyte. Under 5 sccm CO_2 flow: before (a) and after (b) electrolysis with 0.5 mM Fe-*p*-TMA; before (c) and after (d) with 0.5 mM H_2-*p*-TMA; before (e) and after (f) without catalyst. Under CO flow: after electrolysis without catalyst (g) and with 0.5 mM Fe-*p*-TMA (h). Peaks labeled P correspond to porphyrin-related resonances. Attenuation of porphyrin-related peaks near 3–4 ppm and emergence of new peaks are observed after electrolysis for both Fe-*p*-TMA and H_2-*p*-TMA. Trace methanol (*) originates from the catalyst recrystallization procedure. Trace formate is detected at comparable levels (~0.5% faradaic efficiency) across all three conditions under CO_2 flow. 1 mM DMSO was used as an internal standard.

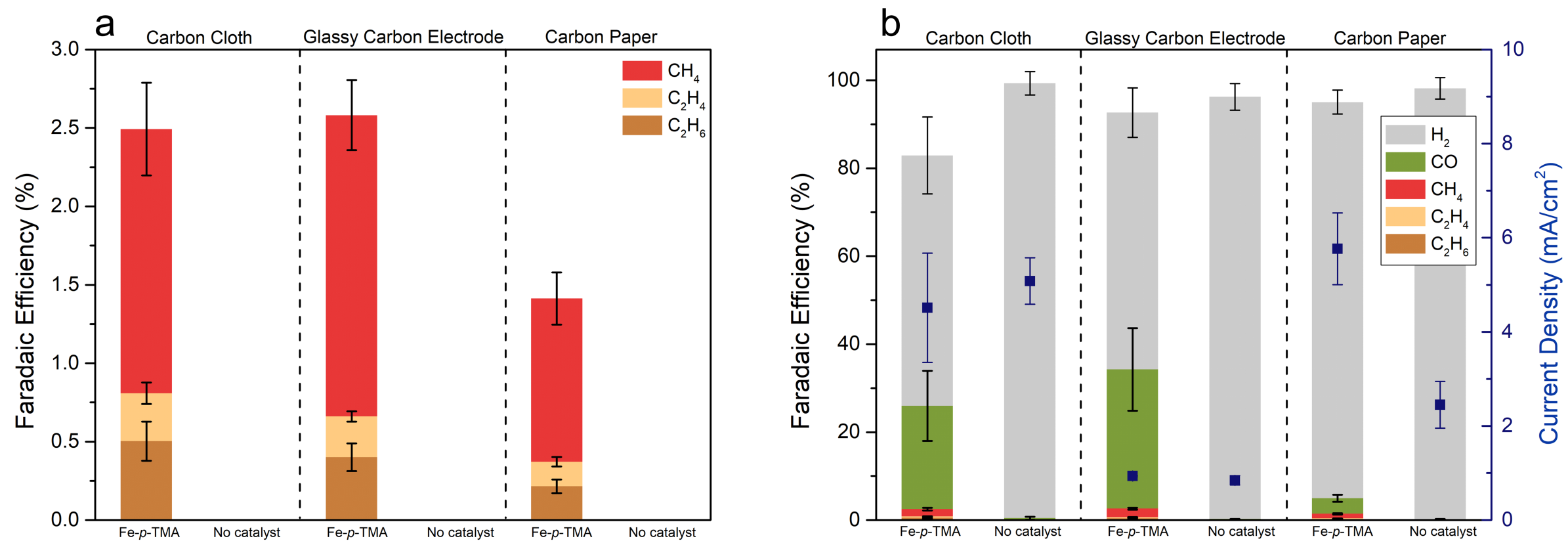

Figure S6. Faradaic efficiencies with different carbon electrodes at 0.5 mM Fe-*p*-TMA in 0.5 M $KHCO_3$ (aq) under 5 sccm CO_2 flow at −0.9 V vs. RHE for (a) CH_4, C_2H_4, and C_2H_6 and for (b) all gaseous products with current density (right). Error bars represent the standard deviation of three independent experiments.

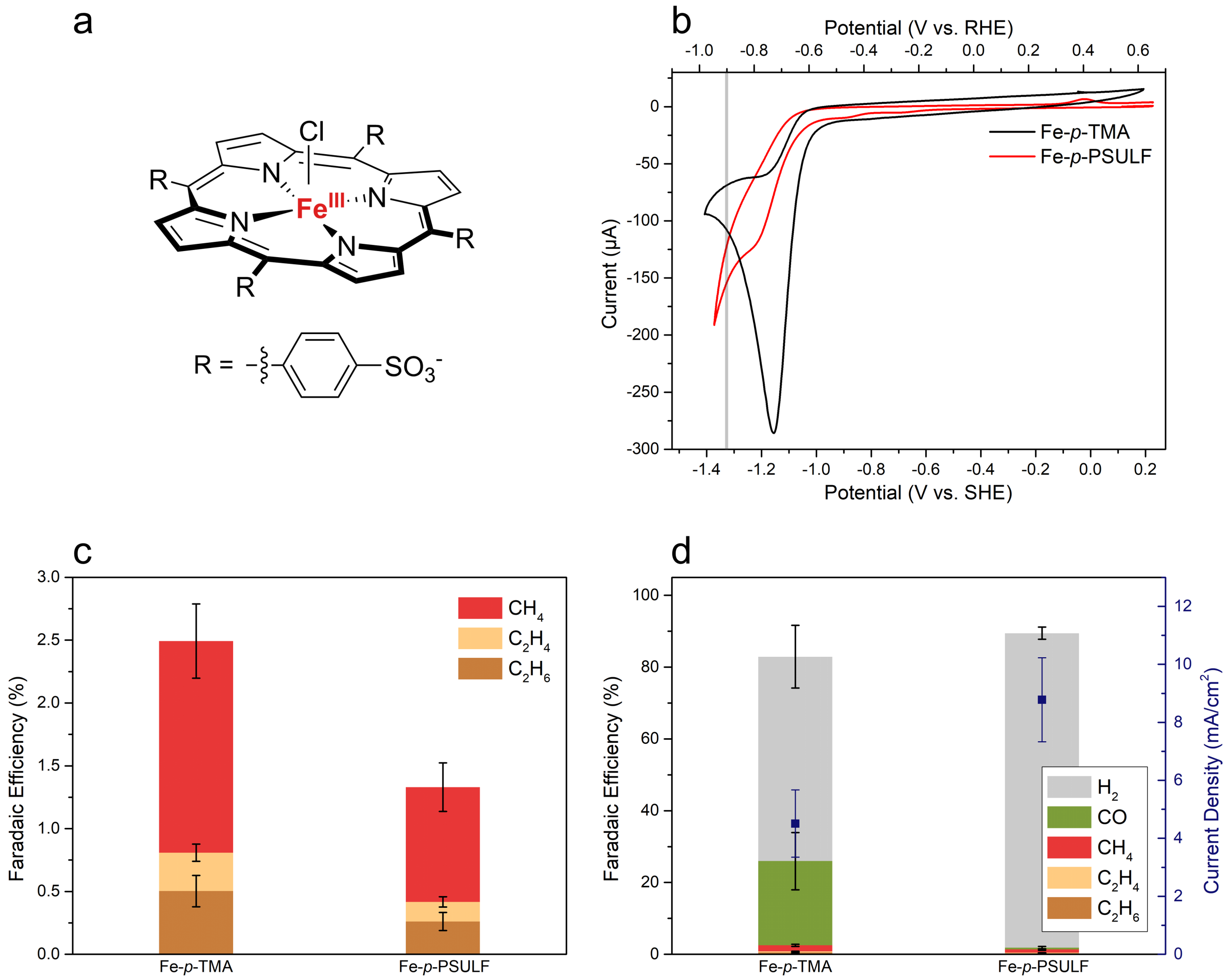

Figure S7. (a) Structure of Fe-*p*-PSULF. (b) Cyclic voltammograms of 0.5 mM Fe-*p*-TMA and Fe-*p*-PSULF in 0.5 M $KHCO_3$ under CO_2 at a scan rate of 100 mV/s. Both reference scales are shown: potential versus RHE on the upper axis and potential versus SHE on the lower axis. The gray vertical line indicates the electrolysis potential of −0.9 V vs. RHE. Faradaic efficiencies at −0.9 V vs. RHE with 0.5 mM Fe-*p*-TMA and Fe-*p*-PSULF on a carbon cloth electrode in 0.5 M $KHCO_3$ (aq) under 5 sccm CO_2 flow for (c) CH_4, C_2H_4, and C_2H_6 and for (d) all gaseous products with current density (right). Error bars represent the standard deviation of three independent experiments.

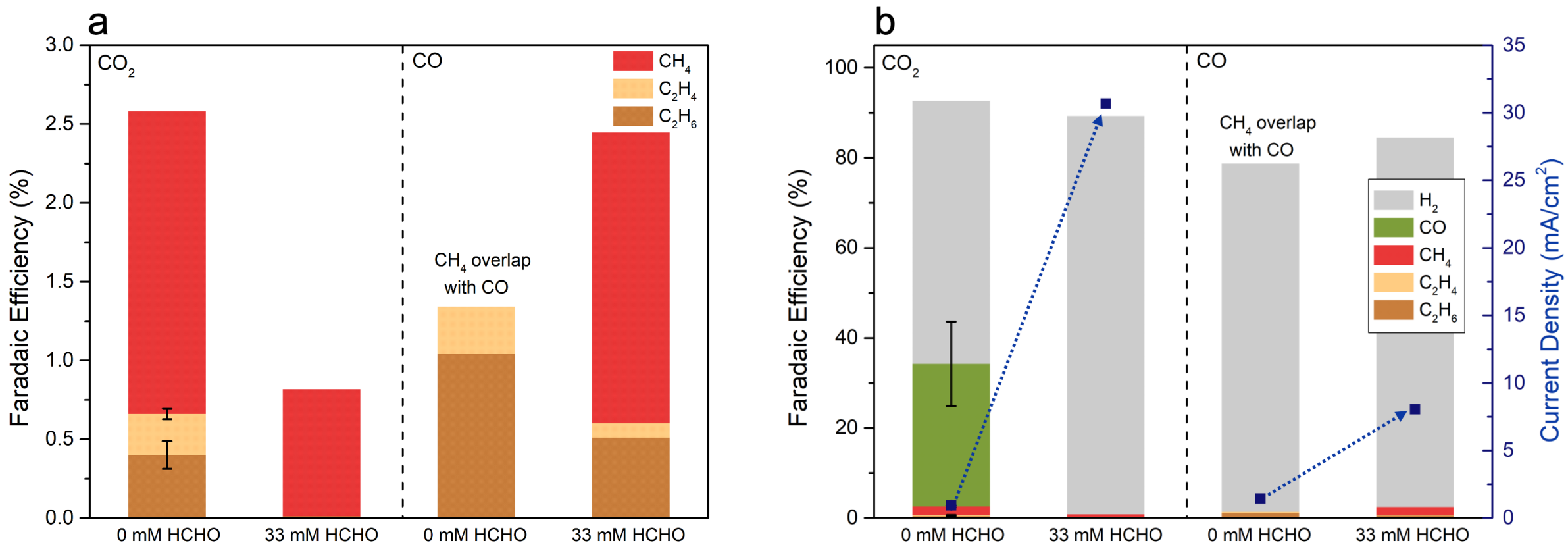

Figure S8. Faradaic efficiencies at −0.9 V vs. RHE on a glassy carbon electrode using 0.5 mM Fe-*p*-TMA in 0.5 M $KHCO_3$ electrolyte under 5 sccm flow of either CO_2 or CO, with and without 33 mM formaldehyde (HCHO), for CH_4, C_2H_4, and C_2H_6 (a) and for all gaseous products with current density (b). Under CO feed, the CH_4 signal overlapped with CO in the gas chromatogram and could not be quantified separately when the current density was low.

2. Rinse Test Method Result

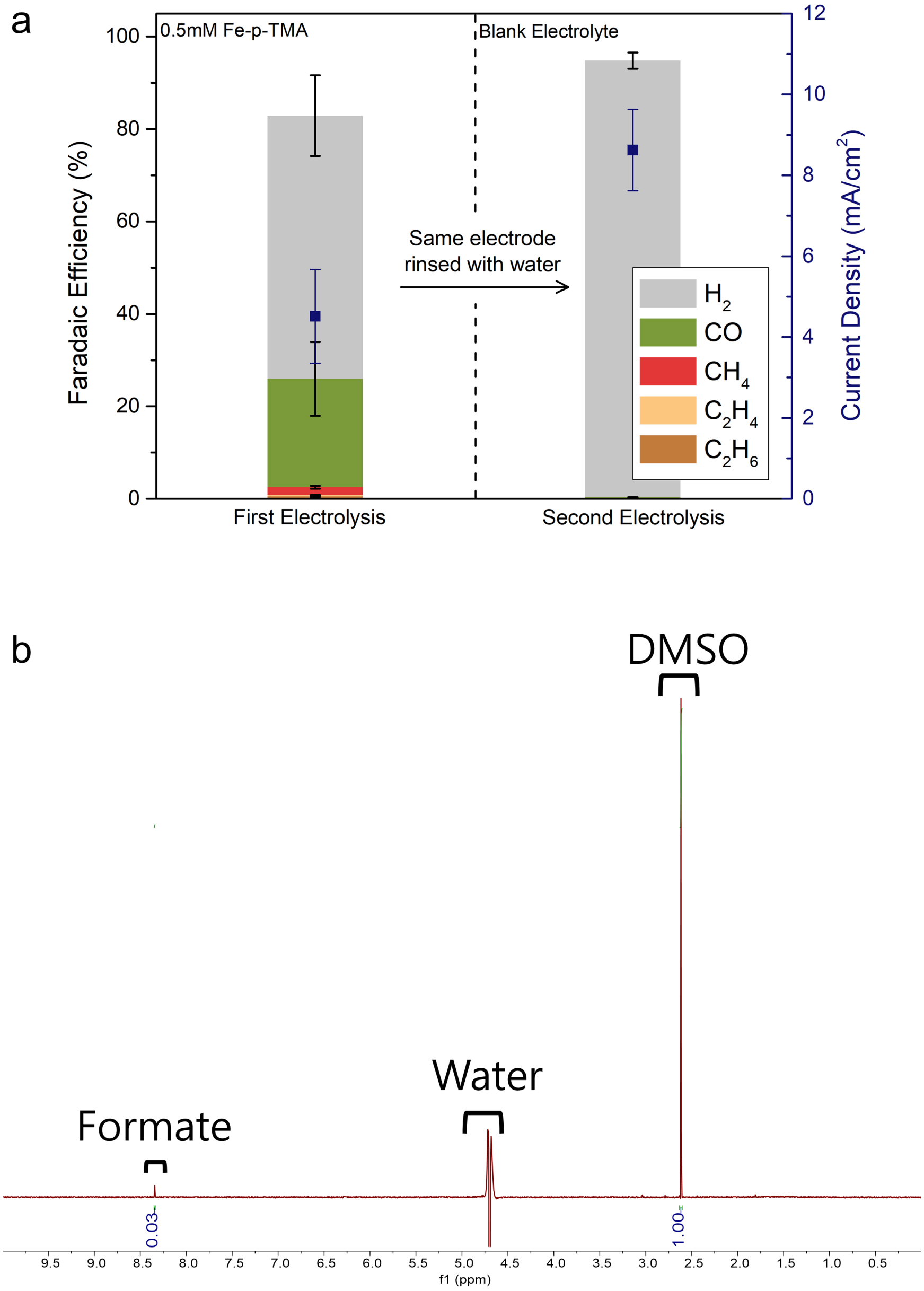

Figure S9. (a) Faradaic efficiencies and current densities for all gaseous products at −0.9 V vs. RHE under 5 sccm CO_2 flow. First electrolysis (left): 0.5 mM Fe-*p*-TMA in 0.5 M $KHCO_3$ (aq). Second electrolysis (right): cathode chamber rinsed and refilled with 0.5 M $KHCO_3$ without Fe-*p*-TMA while maintaining the assembled cell. (b) ^1H NMR spectra of catholyte after the second electrolysis. Error bars represent the standard deviation of three independent experiments.

3. Isotopic Labeling GC-MS Results

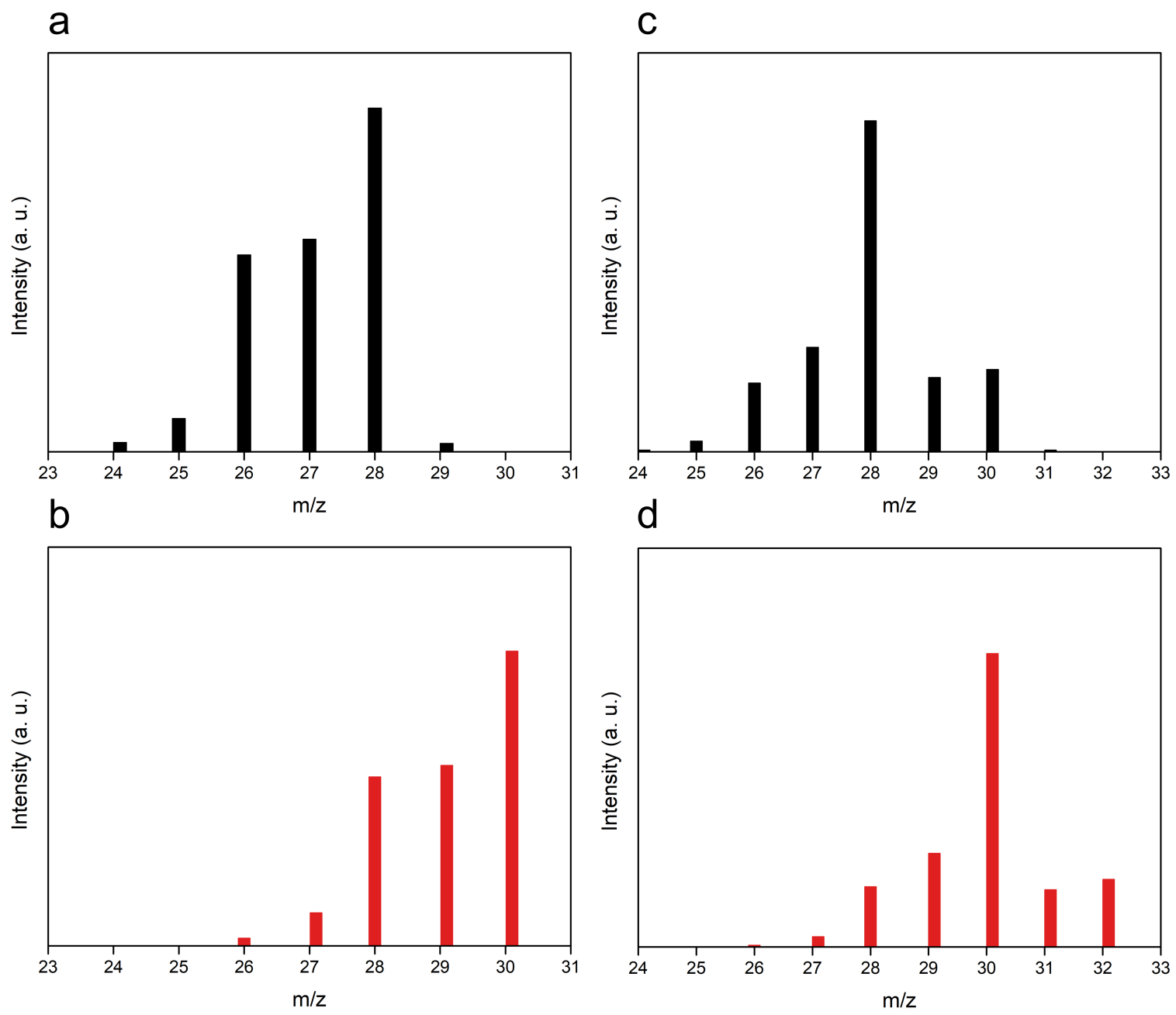

Figure S10. Mass spectra of (a,b) ethylene and (c,d) ethane from the headspace of 0.5 mM Fe-*p*-TMA in 0.5 M $KHCO_3$ in a CO_2-saturated batch cell after electrolysis at −1.0 V vs. RHE, under (a, c) $^{12}CO_2$ with 0.5 M $KHCO_3$ and (b, d) $^{13}CO_2$ with 0.5 M $NaH^{13}CO_3$.

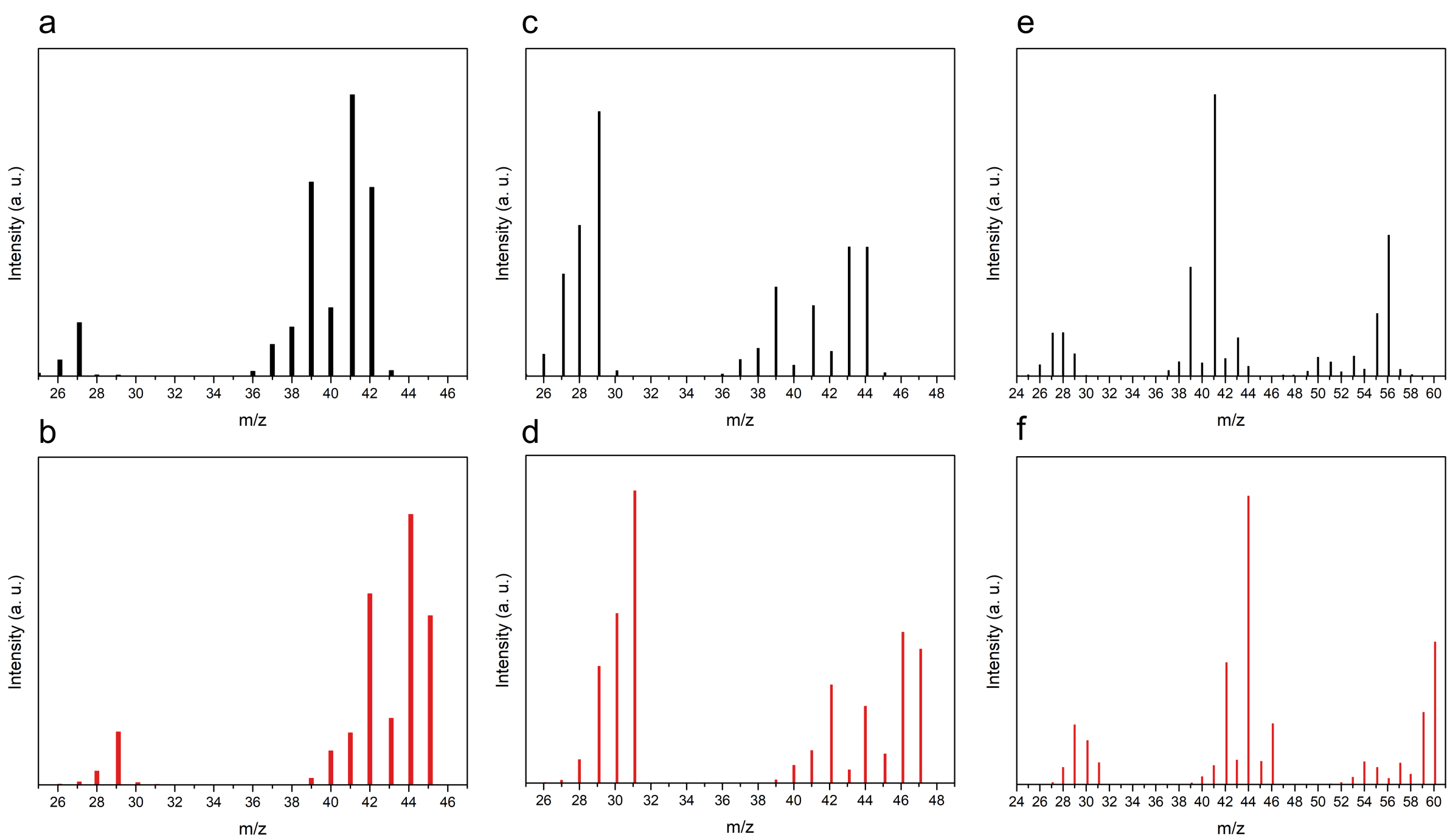

Figure S11. Mass spectra of (a,b) propylene, (c,d) propane, and (e,f) butylene from the headspace of 0.5 mM Fe-*p*-TMA in 0.5 M $KHCO_3$ in a CO_2-saturated batch cell after electrolysis at −1.0 V vs. RHE, under (a, c, e) $^{12}CO_2$ with 0.5 M $KHCO_3$ and (b, d, f) $^{13}CO_2$ with 0.5 M $NaH^{13}CO_3$.

4. Concentration Dependence Results

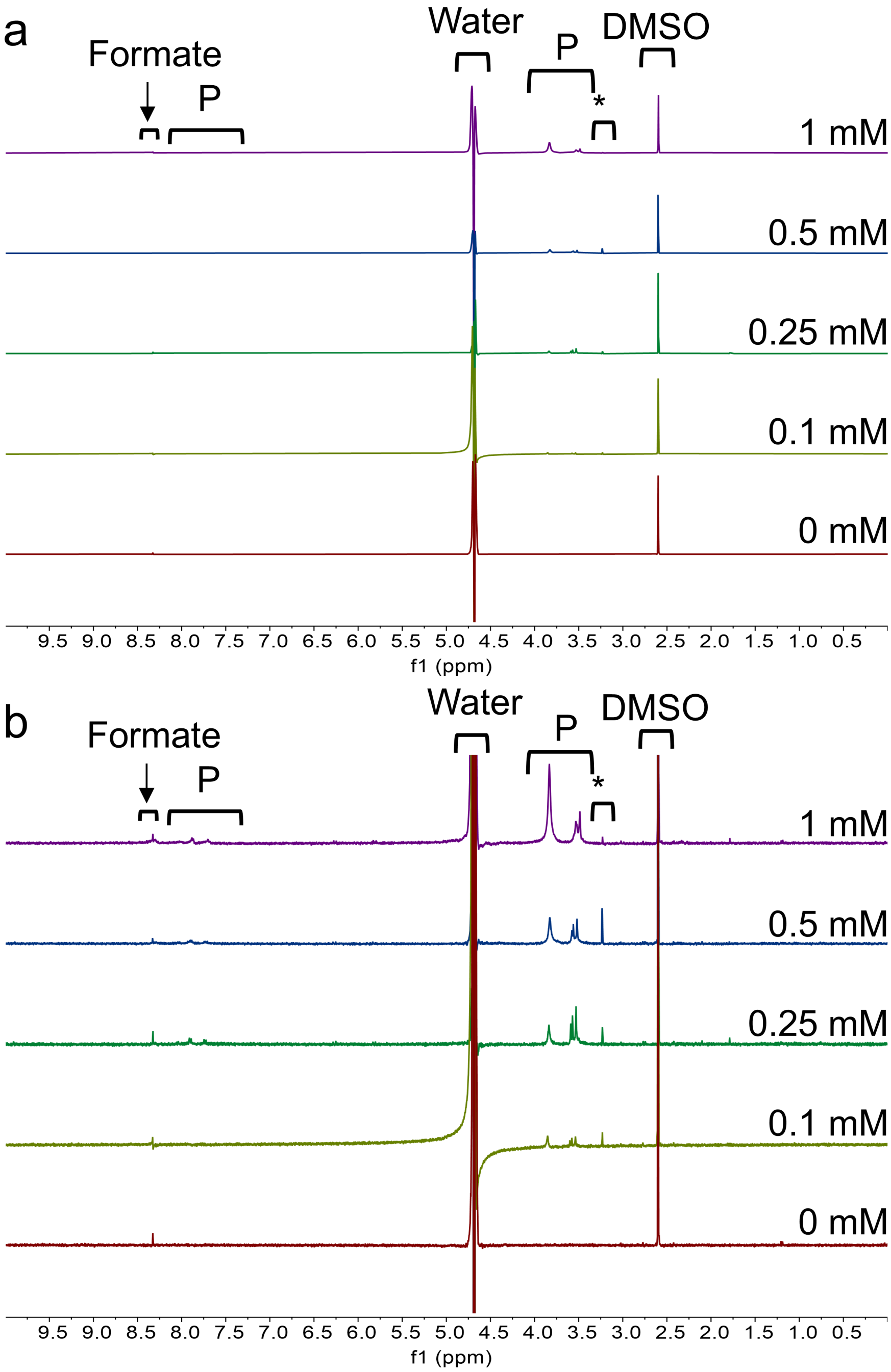

Figure S12. ^{1}H NMR spectra of catholyte after 30 min electrolysis as a function of Fe-*p*-TMA concentration (0–1 mM) at −0.9 V vs. RHE using 0.5 M $KHCO_3$ electrolyte under 5 sccm CO_2 flow: (a) full spectra and (b) expanded view. Peaks labeled P correspond to porphyrin-related resonances. Attenuation of porphyrin-related peaks near 3–4 ppm and emergence of new peaks are observed after electrolysis. Trace methanol (*) originates from the catalyst recrystallization procedure. Trace formate is detected at all concentrations, including the catalyst-free control. 1 mM DMSO was used as an internal standard.

5. Catalyst Stability Test Result

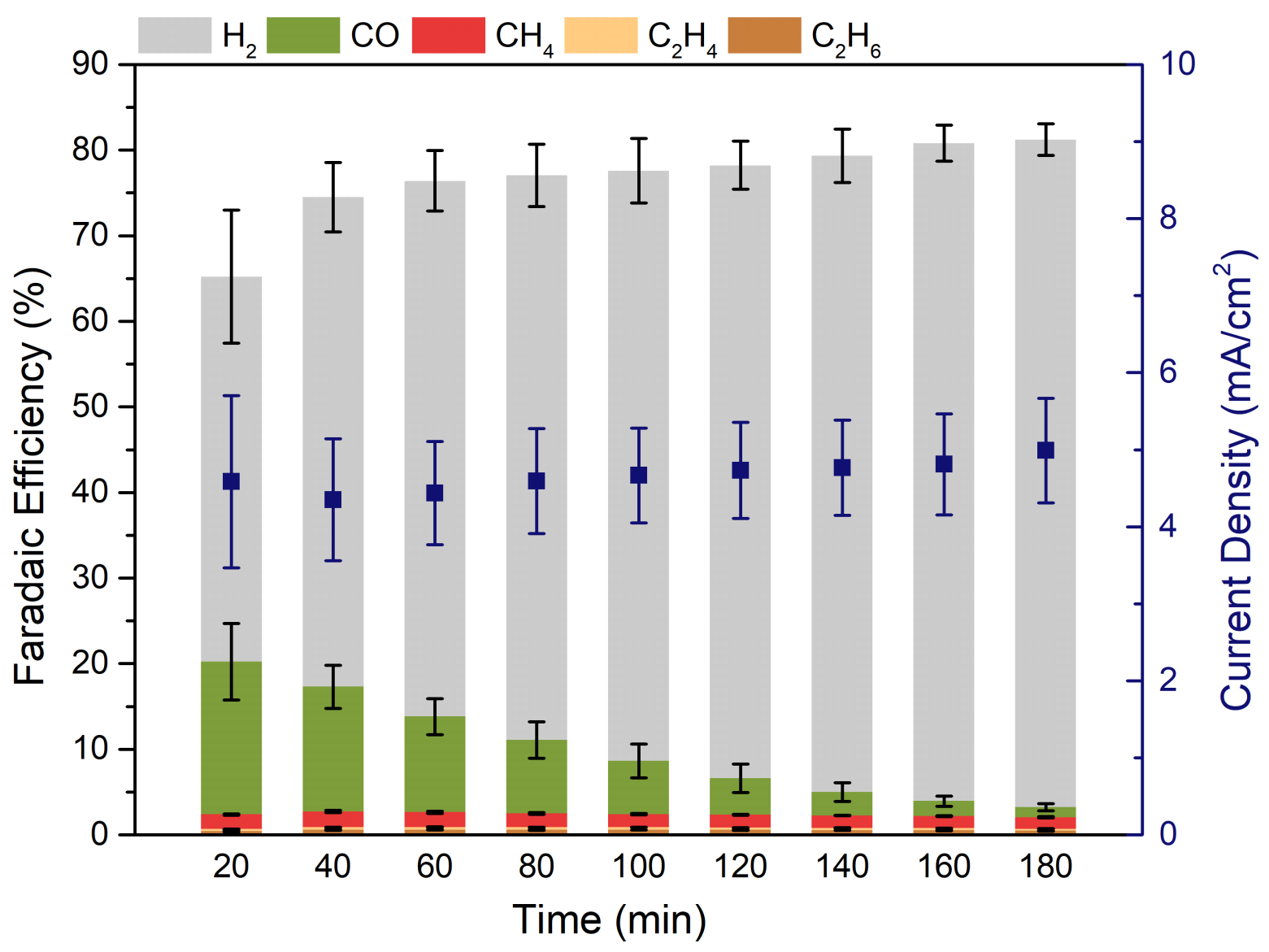

Figure S13. Faradaic efficiencies for all gaseous products and current density as a function of electrolysis time at −0.9 V vs. RHE using 0.5 mM Fe-*p*-TMA in 0.5 M $KHCO_3$ electrolyte under 5 sccm CO_2 flow. Data collected in 20 min intervals over 3 h. The initial low faradaic efficiency at 20 min arises from the GC inlet gas not yet reaching equilibrium with the product gas. Error bars represent the standard deviation of three independent experiments.

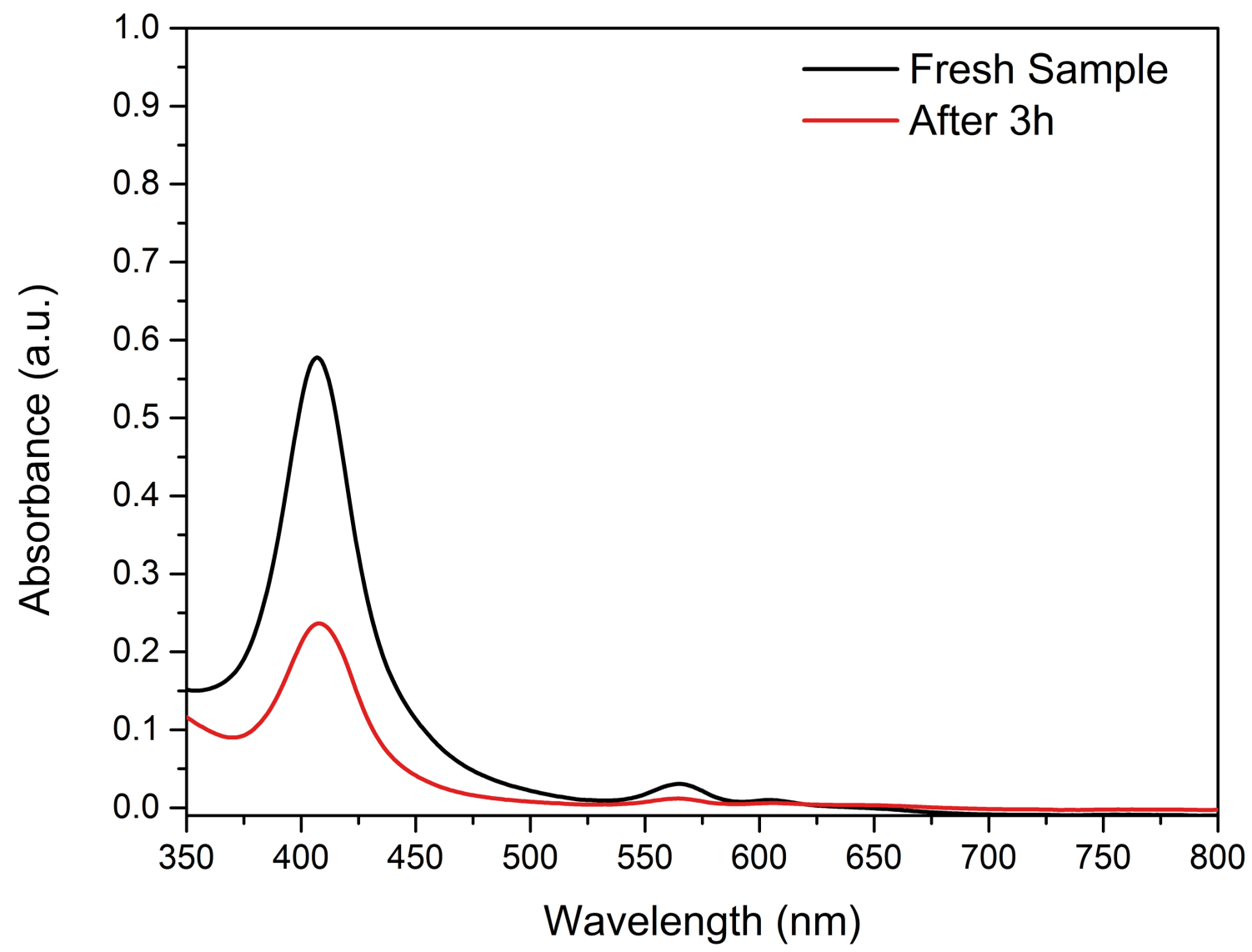

Figure S14. UV–vis spectra of the diluted catholyte before and after 3 h electrolysis at −0.9 V vs. RHE using 0.5 mM Fe-*p*-TMA in 0.5 M $KHCO_3$ electrolyte under 5 sccm CO_2 flow. Samples were diluted to 20 µM Fe-*p*-TMA concentration with 0.5 M $KHCO_3$ prior to measurement.

6. Electrode Characterization before and after Electrolysis

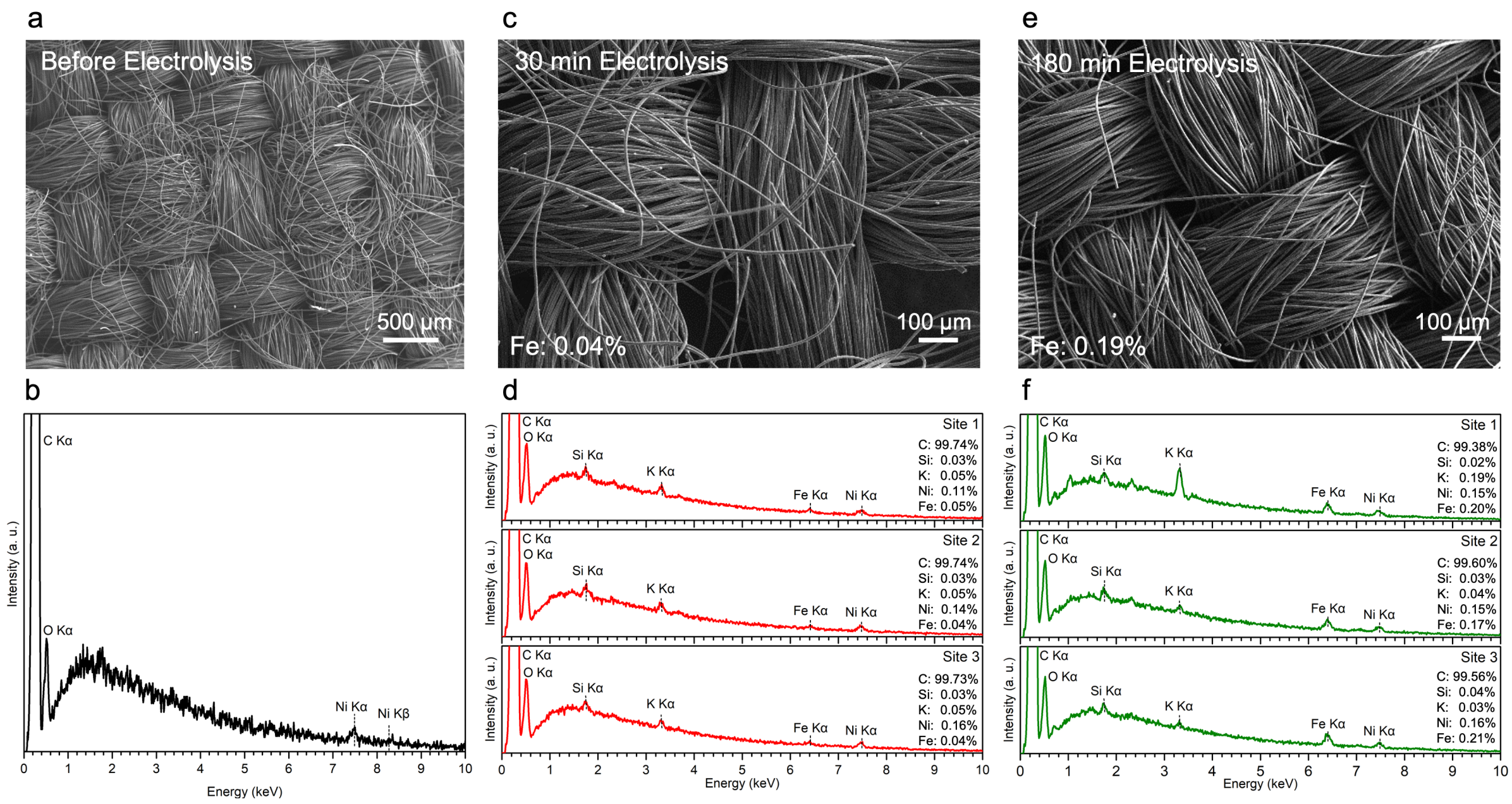

Figure S15. SEM images and corresponding EDS elemental analyses of the carbon cloth electrode (a,b) before electrolysis, (c,d) after 30 min of electrolysis, and (e,f) after 180 min of electrolysis at −0.9 V vs. RHE using 0.5 mM Fe-*p*-TMA in 0.5 M $KHCO_3$ (aq) under 5 sccm CO_2 flow. Three different sites were measured and averaged for post-electrolysis electrode samples.

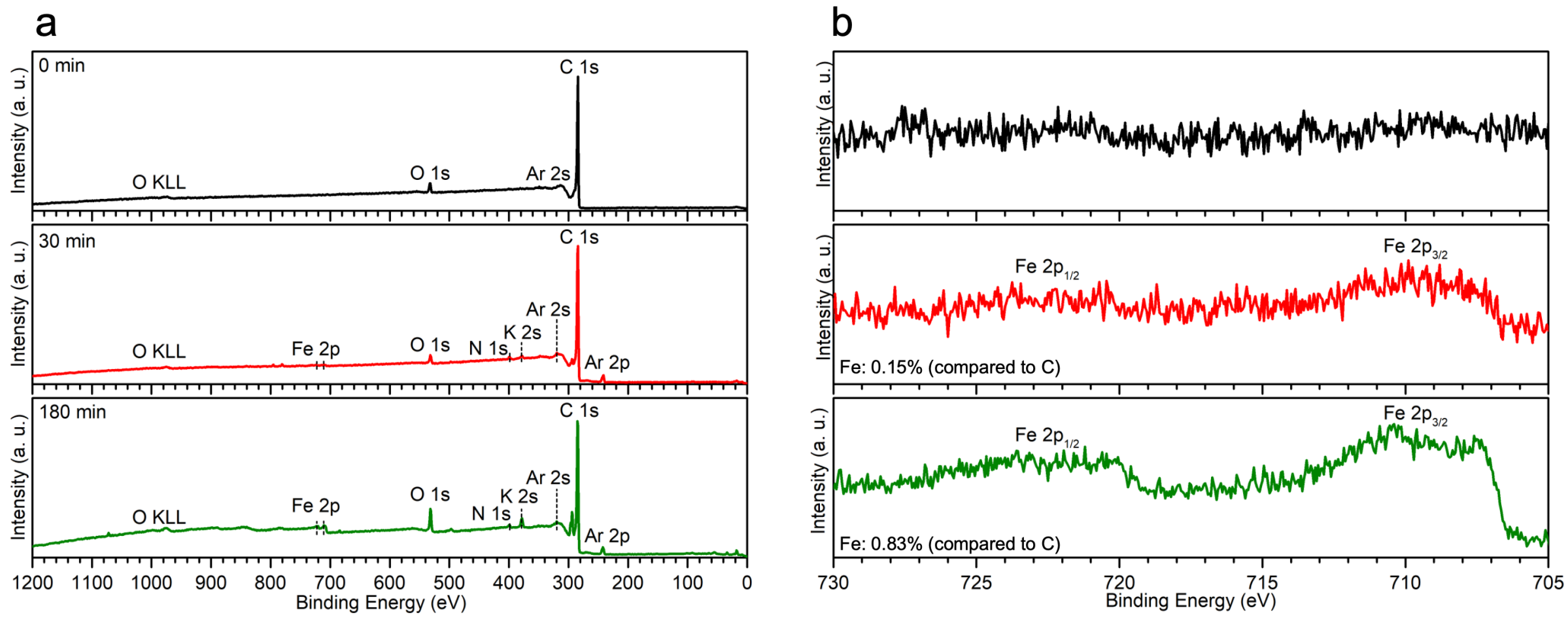

Figure S16. XPS analysis of the carbon cloth electrode before electrolysis and after 30 and 180 min of electrolysis at −0.9 V vs. RHE using 0.5 mM Fe-*p*-TMA in 0.5 M $KHCO_3$ (aq) under 5 sccm CO_2 flow: (a) survey spectra and (b) high-resolution spectra of the Fe 2p region.

7. Estimation of Diffusion Layer Thickness

Reduction of protons on Pt electrode is a fast electrochemical process that gives a plateau current in the mass-transport-limited region, at potentials positive of water reduction. From this plateau current, the diffusion layer thickness (δ) can be estimated as below:

$$i_{pl} = \frac{FD_{H^+}[H^+]_{bulk}}{\delta}$$

$$i_{pl,0rpm} = 0.25\ mA/cm^2$$

$$i_{pl,1000rpm} = 0.86\ mA/cm^2$$

Taking $D_H = 9.3 \times 10^{-5}$ cm^2/s,[3] F = 96485 C/mol, $[H^+]_{bulk} = 10^{-3.05} = 8.91 \times 10^{-4}$ M, the estimated diffusion layer thicknesses were δ_{0rpm} = 320 µm, $\delta_{1000rpm}$ = 93 µm.

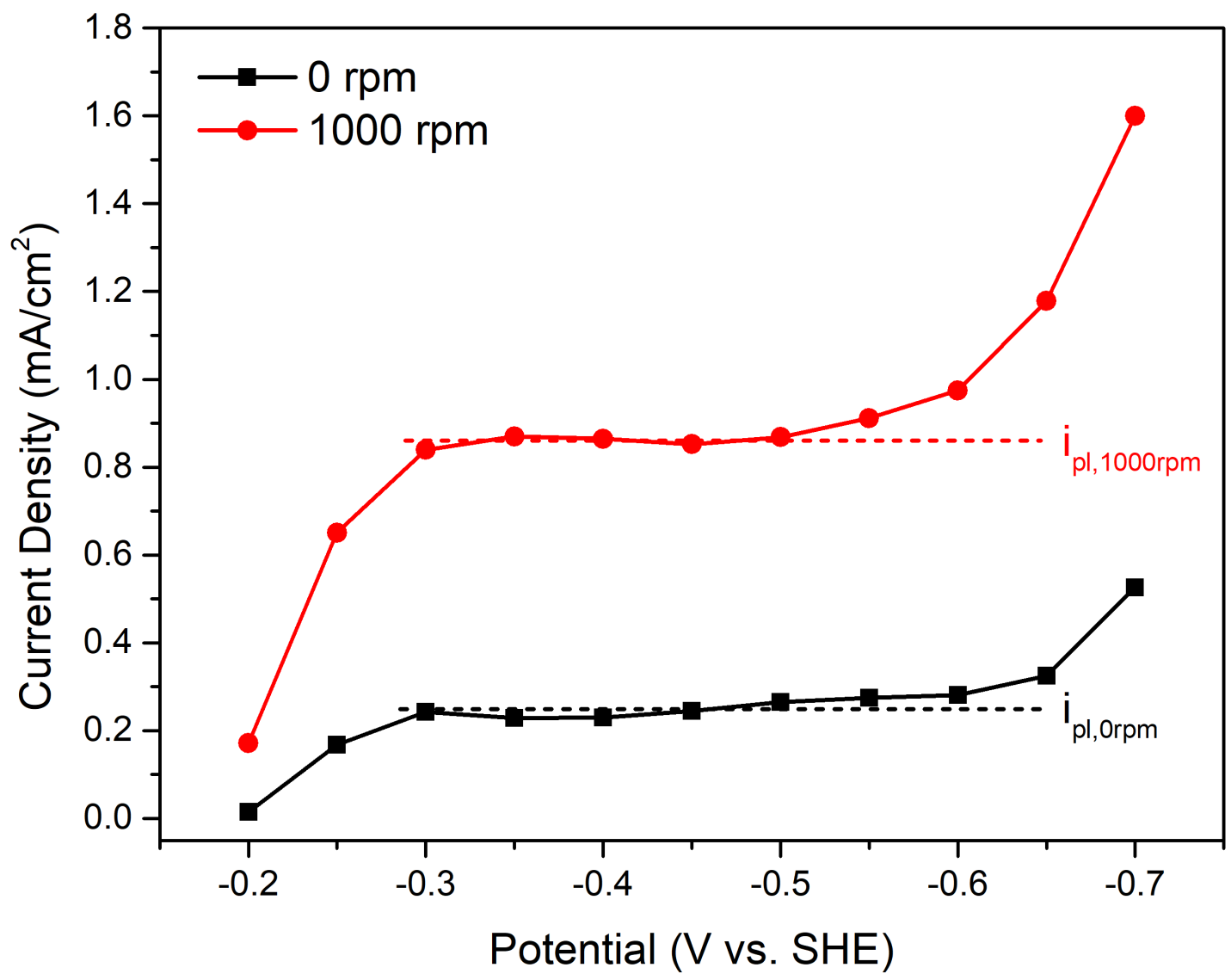

Figure S17. Proton reduction on a flat Pt electrode (exposed area 1 cm^2) in 1 mM HCl with 0.5 M KCl as supporting electrolyte (pH = 3.05), measured under the same hydrodynamic conditions as the controlled potential electrolysis experiments in the gas flow condition: same cell, 5 sccm Ar flow, with and without a stir bar rotated at 1000 rpm. 5 sccm Ar was used instead of CO_2 so that the plateau current reflects hydrogen evolution alone.

8. Spectroelectrochemistry and Cyclic Voltammetry under Ar and CO

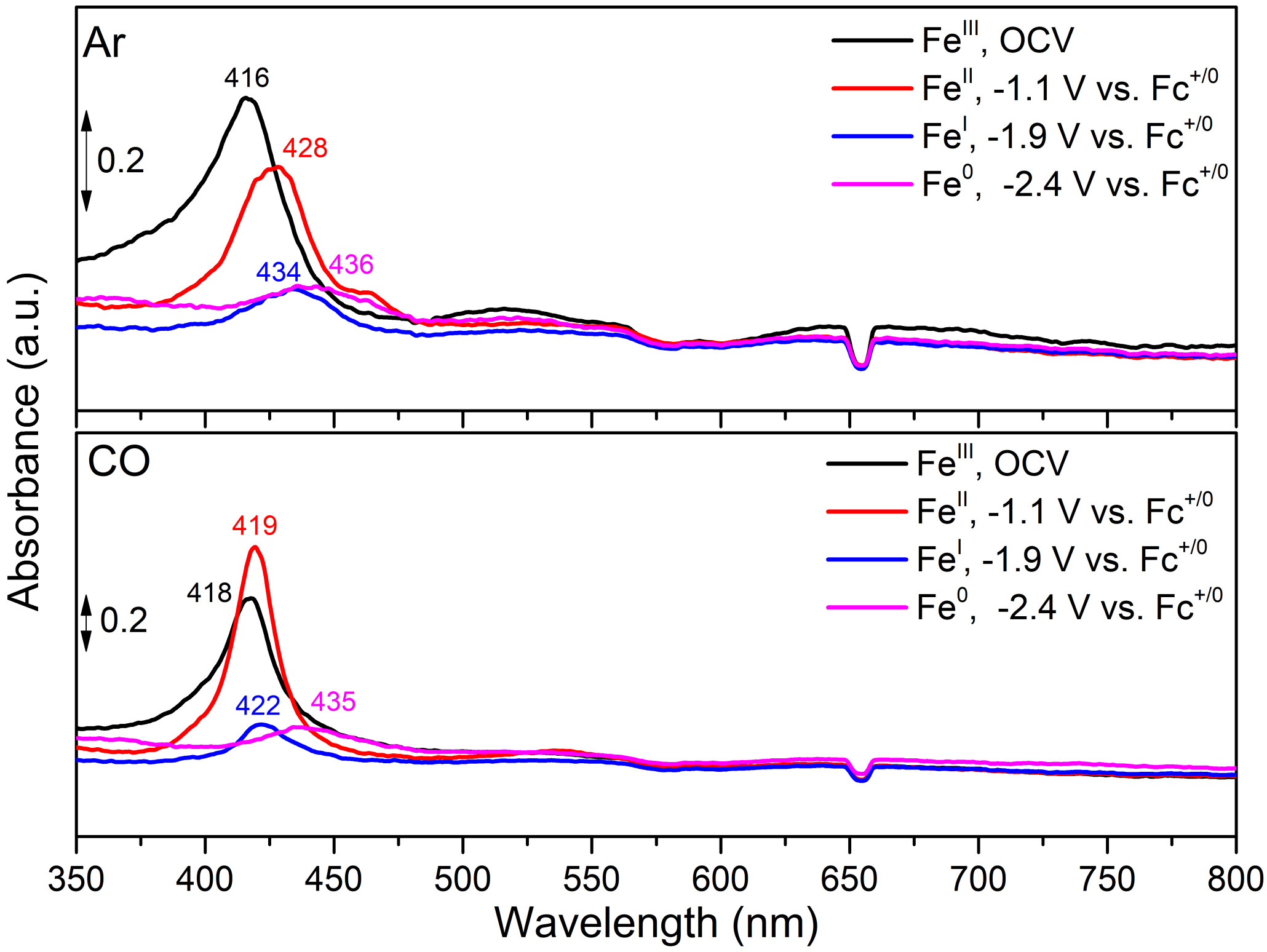

Figure S18. UV–vis spectroelectrochemistry of 0.1 mM Fe-*p*-TMA in 0.1 M $TBAPF_6$/DMF under Ar (top) and CO (bottom) at the indicated potentials vs. $Fc^{+/0}$.

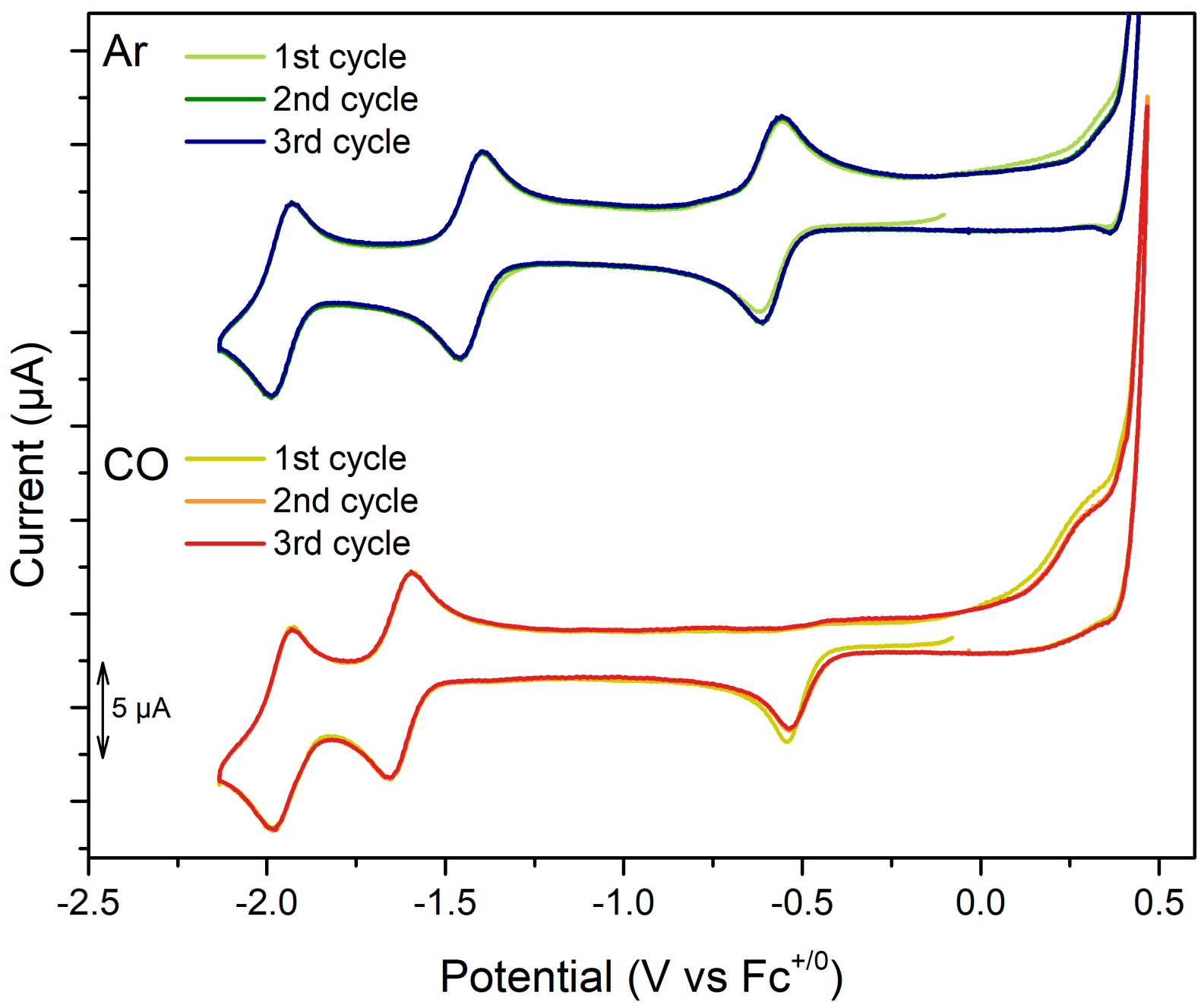

Figure S19. Cyclic voltammograms of 0.5 mM Fe-*p*-TMA in 0.1 M $TBAPF_6$/DMF under Ar (top) and CO (bottom) atmospheres at a scan rate of 100 mV/s.

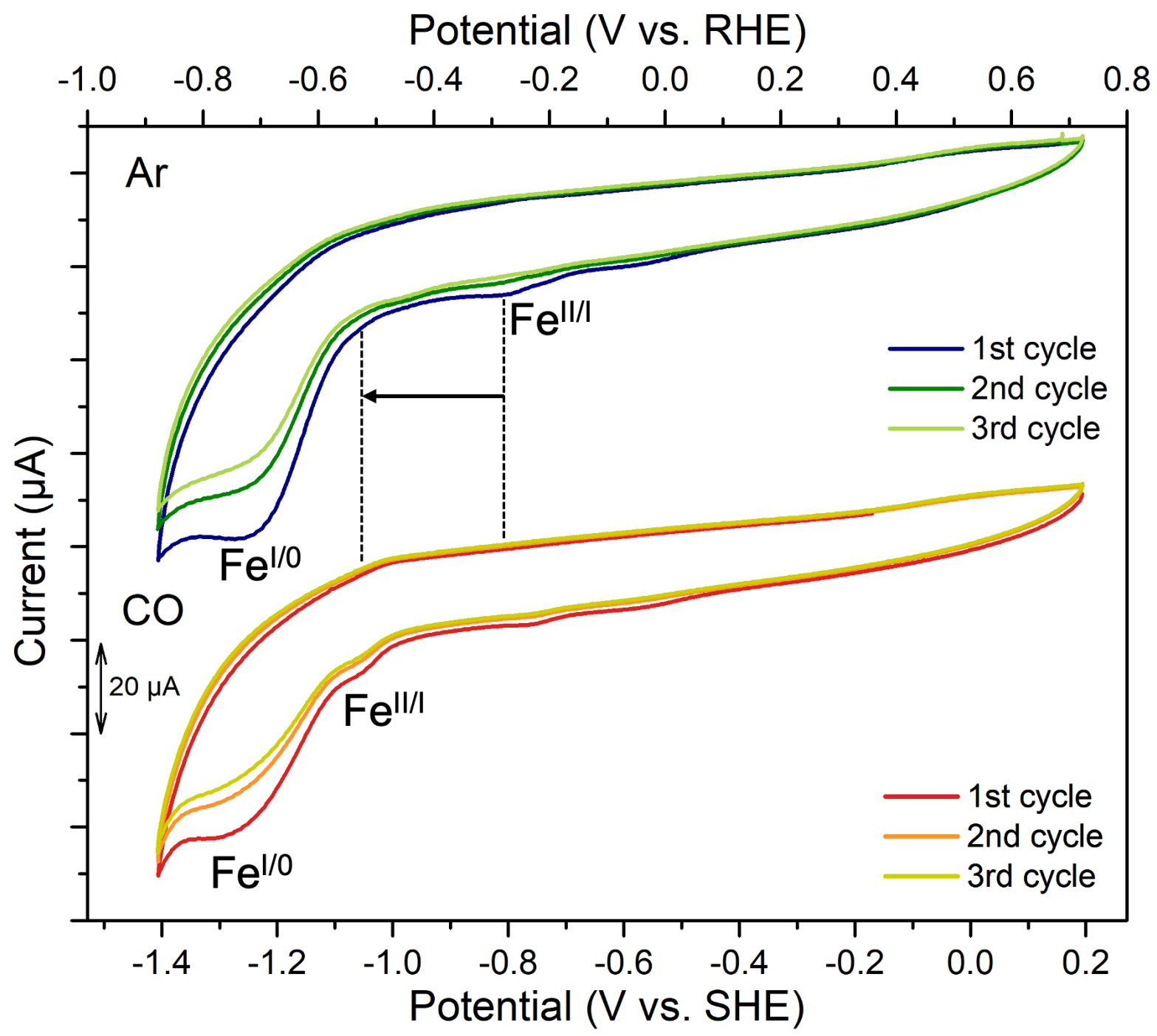

Figure S20. Cyclic voltammograms of 0.5 mM Fe-*p*-TMA in 0.5 M $KHCO_3$ (aq) under Ar (top) and CO (bottom) atmospheres at a scan rate of 100 mV/s. Arrow indicates the negative shift of the $Fe^{II/I}$ couple under CO. Both reference scales are shown: potential versus RHE on the upper axis and potential versus SHE on the lower axis.

9. Stability of Fe^{II}–CO in Organic Solvent

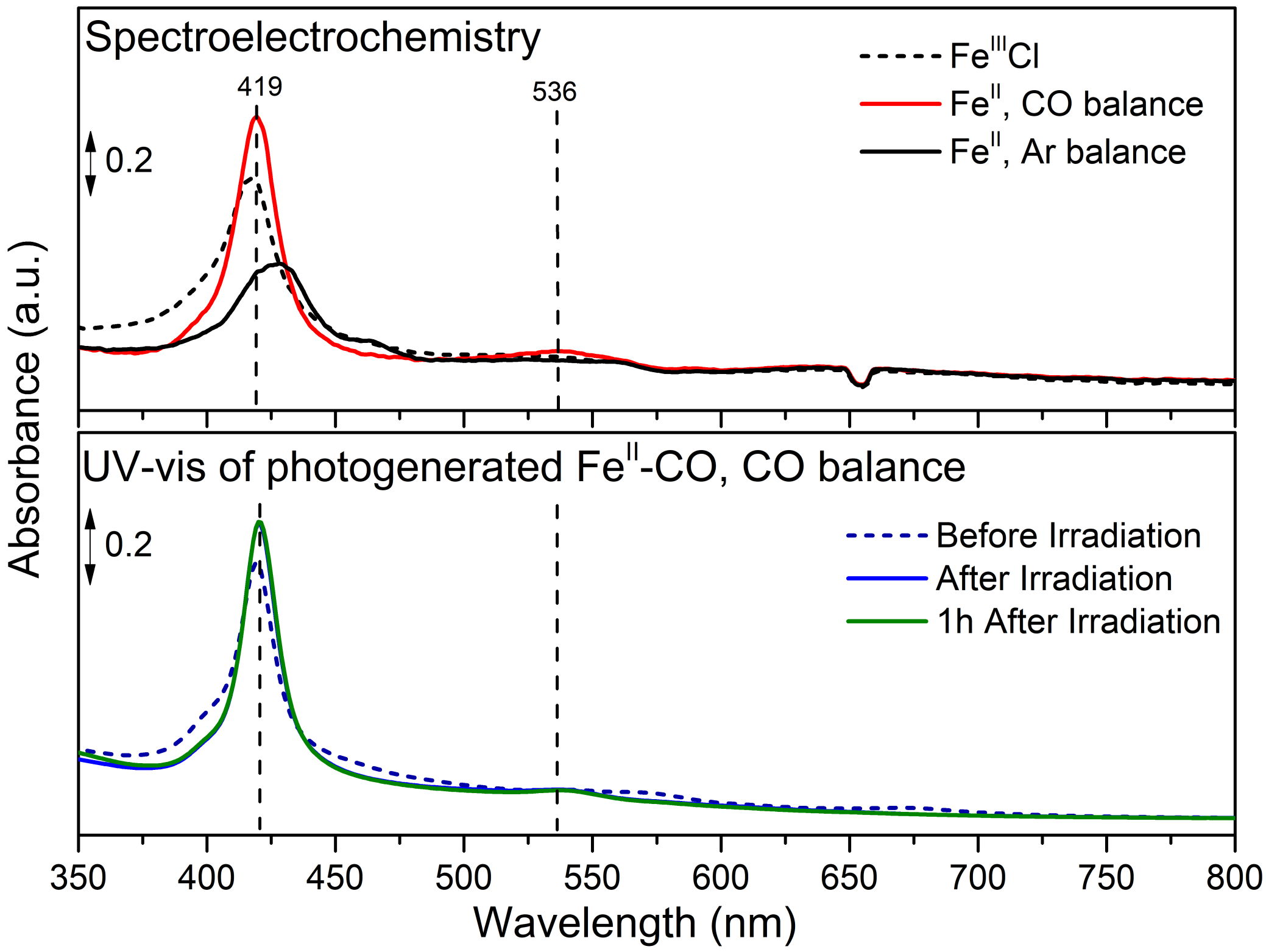

Figure S21. (Top) UV–vis spectroelectrochemistry of 0.1 mM Fe-*p*-TMA in 0.1 M $TBAPF_6$/DMF at open-circuit potential (dashed), and at −1.1 V vs. $Fc^{+/0}$ under Ar (black) and CO (red). (Bottom) UV–vis of the 20 µM Fe-*p*-TMA in 0.1 M $TBAPF_6$/DMF before (dashed) and after 390 nm irradiation (blue), and 1 h post-irradiation (green). The blue and green traces overlap completely, confirming persistence of the photogenerated Fe^{II}–CO species.

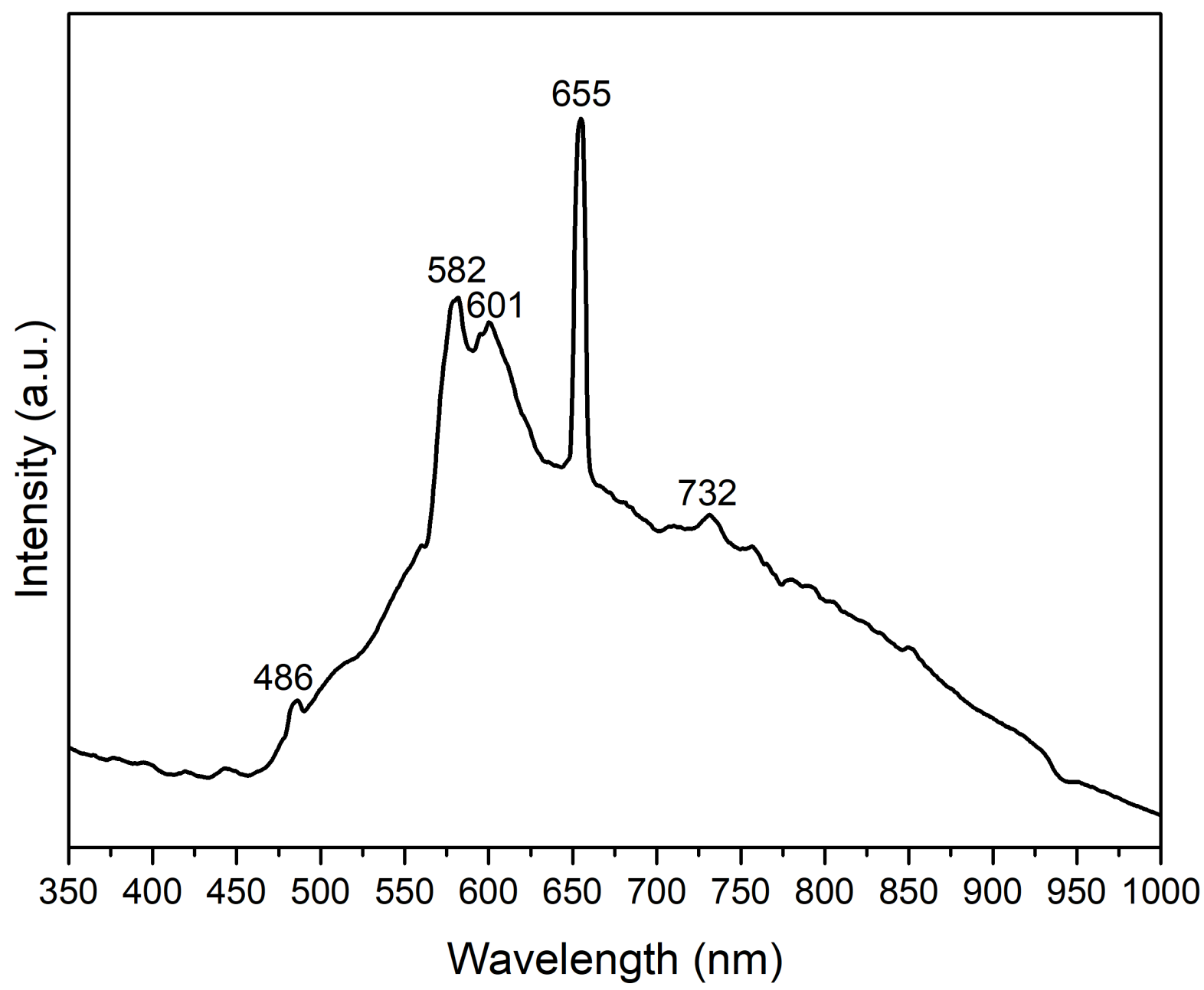

Figure S22. Emission spectrum of the combined tungsten–halogen and deuterium light source used for the UV–vis spectroelectrochemistry setup, recorded with blank electrolyte. Labeled features correspond to the deuterium Balmer lines at 486 and 655 nm.

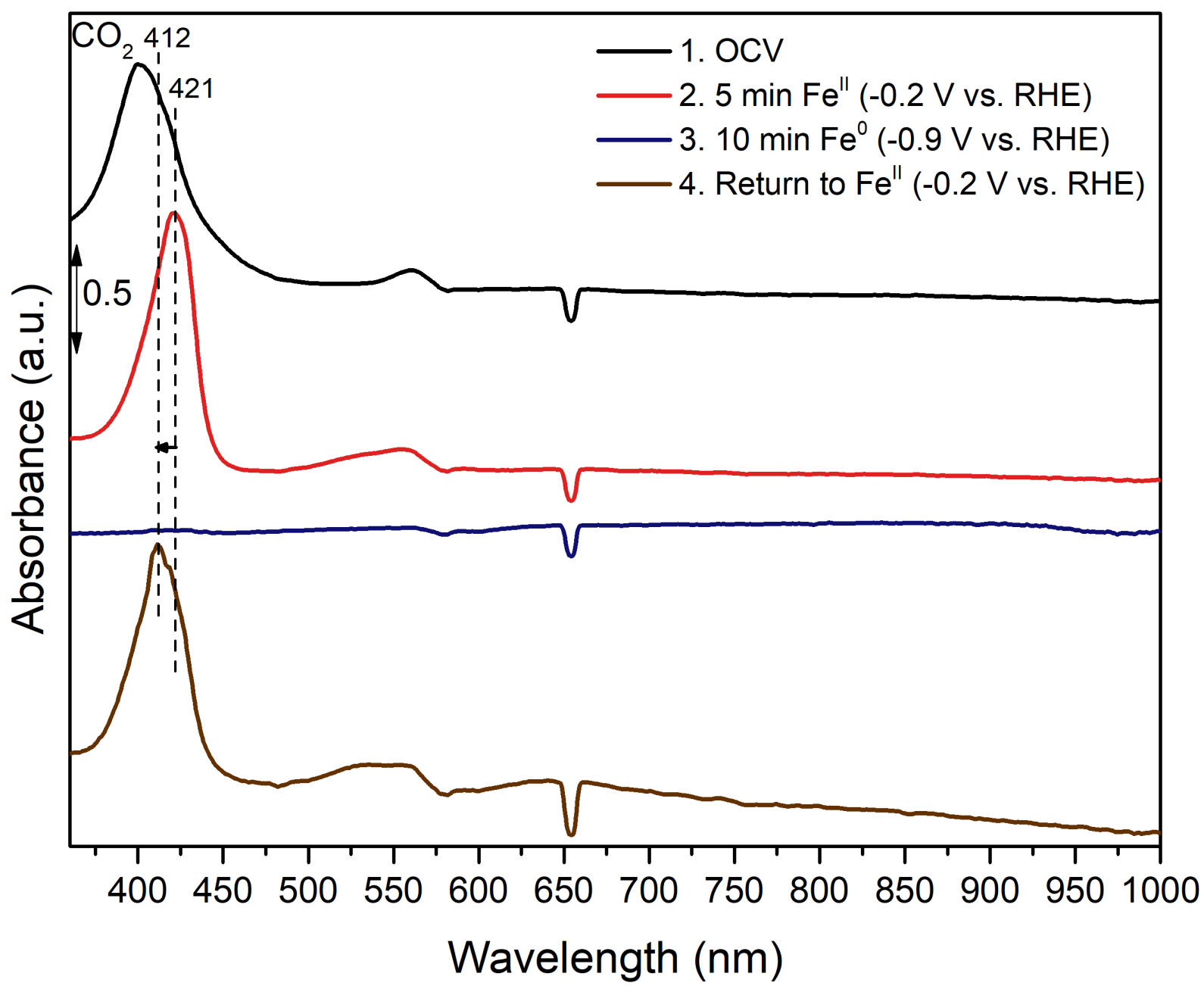

Figure S23. UV–vis spectroelectrochemistry of 0.5 mM Fe-*p*-TMA in CO_2-saturated 0.5 M $KHCO_3$ (aq) at open-circuit potential (black), after 5 min at −0.2 V vs. RHE (red), after 10 min at −0.9 V vs. RHE (blue), and upon returning to −0.2 V vs. RHE (brown). The data are the same as in **Figure 9a**, plotted over the full spectral range.

10. Literature Summary of Electrochemical Fischer–Tropsch on Carbon and Iron Electrodes

Table S3. Summary of selected literature on electrochemical Fischer–Tropsch chemistry in aqueous electrolytes using carbon and iron electrodes. All values are faradaic efficiencies in % and are as reported.

Electrolysis System	Pressure /Voltage (vs. Ag/AgCl)	Electrolyte	CH_4	C_2H_4	C_2H_6	C_3H_6	C_3H_8	C_4H_8	C_4H_{10}	CO	H_2	HCOOH	HC total
0.5 mM Fe-*p*-TMA + carbon cloth [Our system]	1 atm CO_2/ −1.58 V (-1.0 V vs. RHE)	0.5 M $KHCO_3$	1.40	0.22	0.44	0.30	0.37	0.26	0.20	30.4	49.0	trace	3.19
Glassy carbon electrode[4]	30 atm CO_2/ −1.41 V	0.1 M $KHCO_3$	1.20	0.22	0.55	0.19	0.41	n.r.	0.15	5.8	52.5	17.1	2.72
	30 atm CO_2/ −1.52 V	0.1 M $KHCO_3$	1.35	0.16	0.38	0.17	0.28	n.r.	0.11	29.0	43.5	12.6	2.45
Fe plate[5]	1 atm CO_2 (49 atm Ar)/ −1.8 V	0.1 M $KHCO_3$	0	0	0	0	0	0	0	0	n. r.	0	0
Fe sheet[6]	1 atm CO_2/ −2.0 V	0.1 M $KHCO_3$	0.008	0.001 (C_2 combined)		0.000 (C_3–C_5 combined)				0.002	52.51	n.r.	0.01
	1 atm CO/ −2.0 V	0.1 M $KHCO_3$	0.012	0.004 (C_2 combined)		0.005 (C_3–C_5 combined)				-	62.14	n.r.	0.02
Fe wire[7]	30 atm/ −1.37 V	0.1 M $KHCO_3$	1.89	0.21	0.46	0.27	0.39	n.r.	0.22	Trace	62.6	15.7	3.44
	30 atm/ −1.53 V	0.1 M $KHCO_3$	1.24	0.22	0.43	0.25	0.40	n.r.	0.17	4.5	23.1	59.5	2.71

HC = hydrocarbon, n.r. = not reported.

11. CO-Induced Suppression of HER on Glassy Carbon Electrode

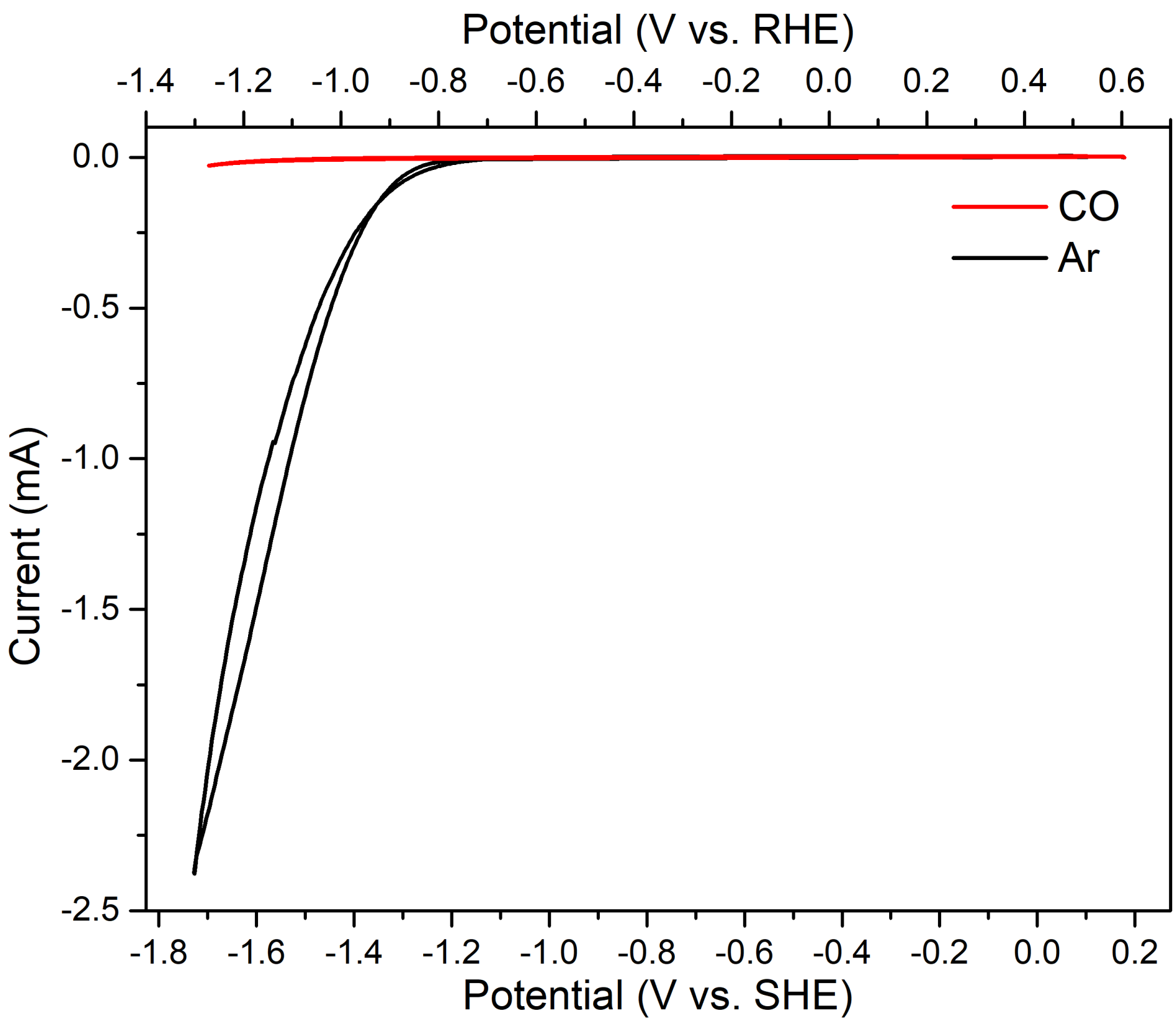

Figure S24. Cyclic voltammograms of catalyst-free glassy carbon electrode in 0.5 M $KHCO_3$ under CO (red) and Ar (black) atmospheres at a scan rate of 100 mV s^{-1}. The suppressed cathodic current under CO relative to Ar is consistent with CO adsorption or site blocking at HER-active electrode-associated sites. Both reference scales are shown: potential versus RHE on the upper axis and potential versus SHE on the lower axis.

12. DOSY Measurements

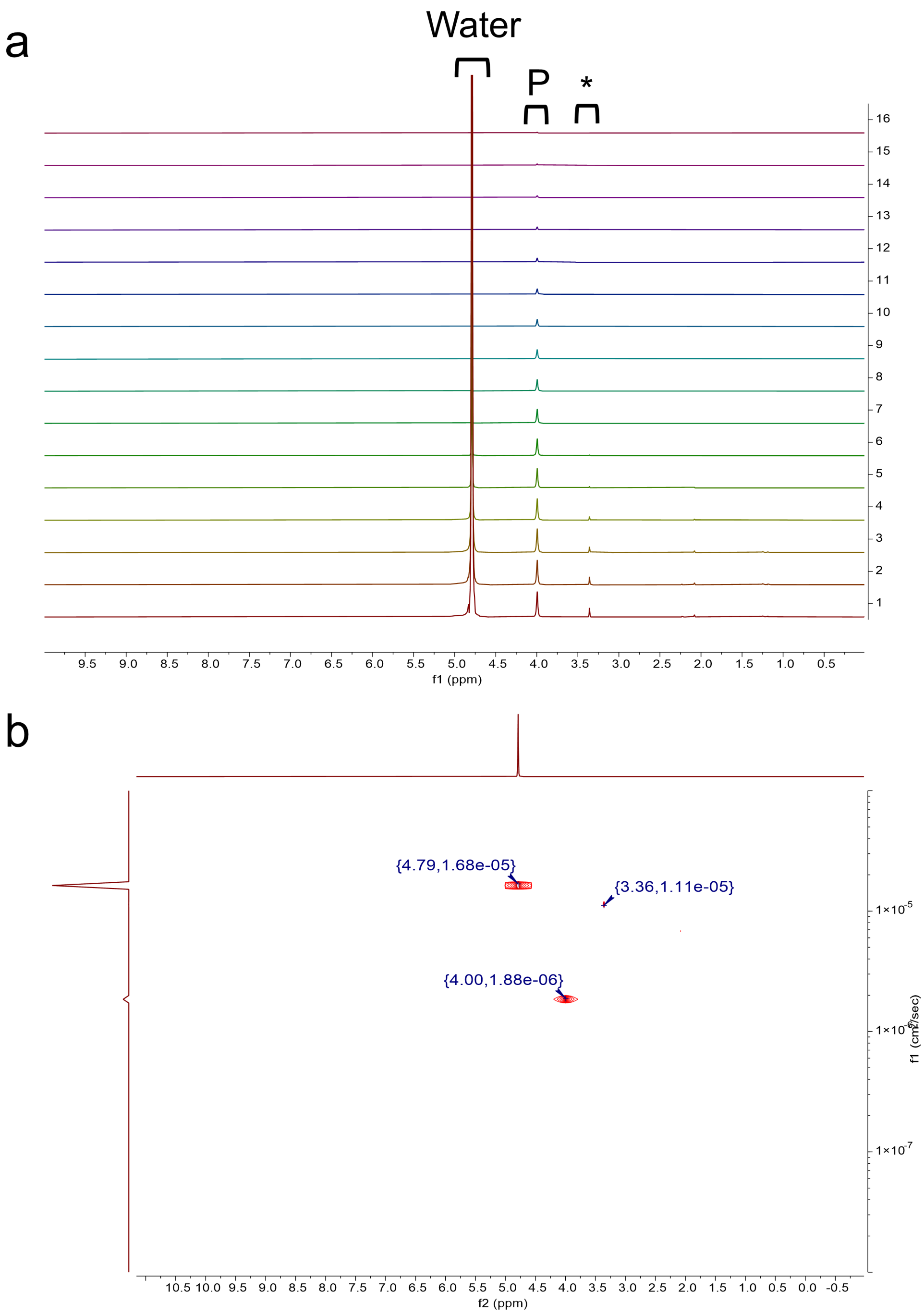

Figure S25. (a) Stacked ^{1}H NMR spectra of 0.5 mM Fe-*p*-TMA in D_2O acquired at varying gradient strengths for DOSY analysis. (b) ^{1}H DOSY spectrum showing diffusion coefficients ($cm^2\ s^{-1}$) as a function of chemical shift. The porphyrin-related resonances (P) at 4.00 ppm correspond to a diffusion coefficient of $1.88 \times 10^{-6}\ cm^2\ s^{-1}$. Trace methanol (*) originates from the catalyst recrystallization procedure. The diffusion coefficient reported in the main text represents the average of three independent measurements.

13. Scan-Rate-Dependent Cyclic Voltammetry

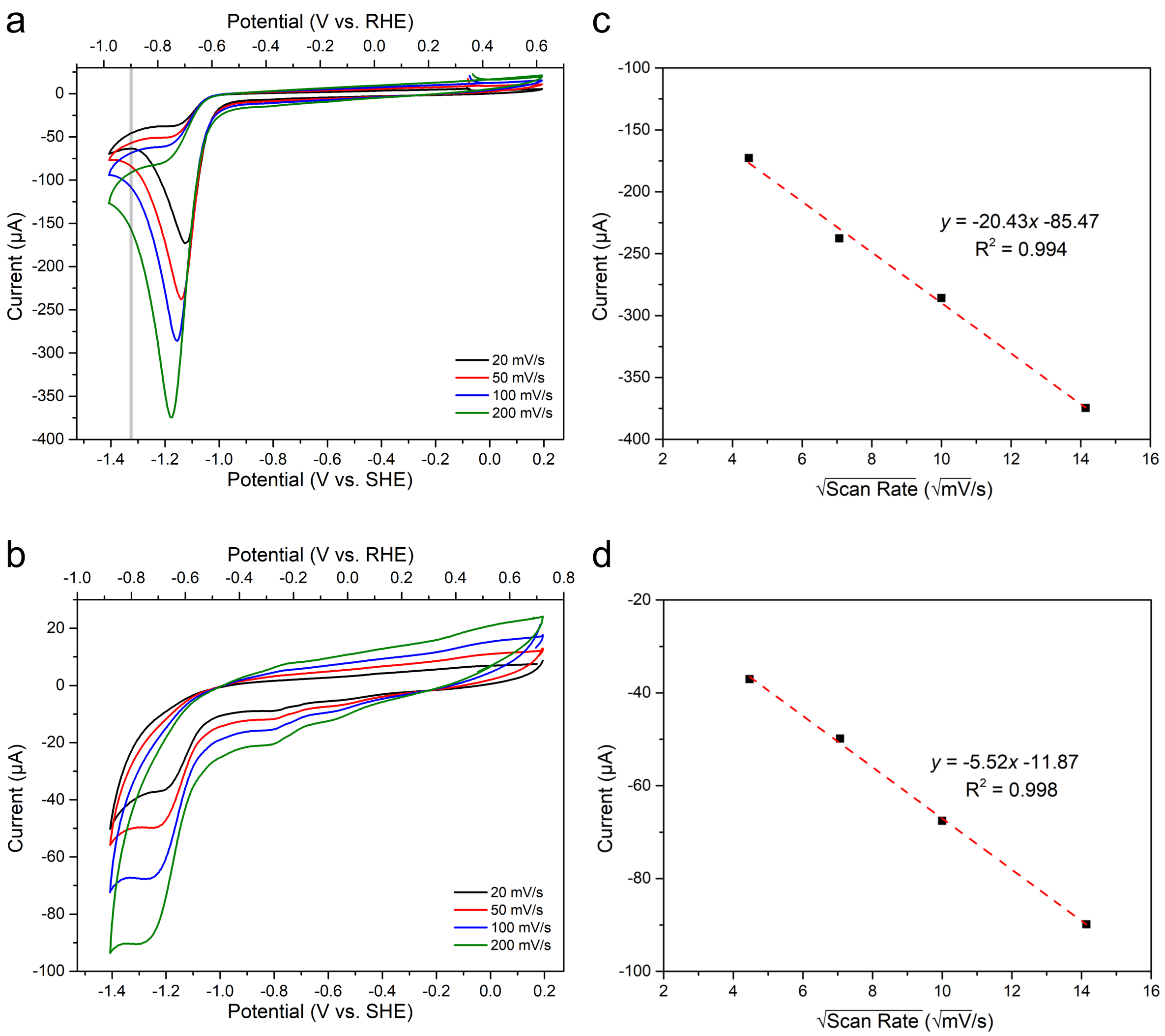

Figure S26. Scan-rate-dependent cyclic voltammetry of 0.5 mM Fe-*p*-TMA in 0.5 M $KHCO_3$ under (a) CO_2 and (b) Ar. Peak current as a function of $\sqrt{\textbf{scan rate}}$ under (c) CO_2 and (d) Ar. Both reference scales are shown: potential versus RHE on the upper axis and potential versus SHE on the lower axis. The RHE scale differs between the CO_2- and Ar-saturated electrolytes because of their 1.73 pH difference (pH 7.23 vs 8.96, measured with AB150 pH meter, Fisher Scientific). The gray vertical line in (a) indicates the electrolysis potential of −0.9 V vs. RHE.

14. Estimation of Reaction-Diffusion Layer Thickness and the Local CO Concentration at the Electrode

If the catalysis were limited by diffusion of the catalyst across the diffusion layer, the CO partial current density would be,

$$j_{DK} = \frac{FD_p C_{cat}^0}{\delta}$$

In the fast catalysis regime, the reaction is confined to a reaction-diffusion layer of thickness μ:

$$j_{FK} = \frac{FD_p C_{cat}^0}{\mu}, \mu = \frac{j_{DK}}{j_{FK}}\delta$$

Taking $D_p = 1.88 \times 10^{-6}$ cm^2/s (**Figure S25**), F = 96485 C/mol, δ = 93 μm (**Figure S17**), the current density in the simple diffusion case was calculated in **Table S4.** One electron per electrode encounter was assumed, following the literature procedure[8] and as the resting state is reported to be Fe^{I}.[9] Assuming two electrons per encounter would increase μ by a factor of two.

The CO partial current density is related to the CO concentration at the electrode surface by

$$j_{CO} = \frac{2FD_{CO}[CO]_{electrode}}{\delta}$$

where the factor of two accounts for the two electrons transferred per CO formed.

For the calculation of the surface CO concentration, the reaction-diffusion layer was assumed to be negligibly thin, so that CO formation occurs at the electrode surface. This would give an upper bound on the surface CO concentration. Planar linear diffusion was assumed. The perturbation of the diffusion layer by H_2 bubble evolution was not accounted for; such convection would thin the diffusion layer and further lower the surface CO concentration. Taking $D_{CO} = 2.03 \times 10^{-5}$ cm^2/s (in pure water),[10] F = 96485 C/mol, δ = 93 μm, the local CO concentration at the electrode was estimated as listed in **Table S4**. No correction was applied for the solubility limit of CO (~1 mM), as the estimate is intended to provide an upper bound on the surface CO concentration.

Table S4. Partial current of CO on a glassy carbon electrode as a function of Fe-*p*-TMA concentration (0–0.5 mM) in 0.5 M $KHCO_3$ (aq) under 5 sccm CO_2 flow at −0.9 V vs. RHE with a stirring rate of 1000 rpm. Values are averages of three independent experiments; full faradaic efficiencies and standard deviations are in **Figure 6**. Estimated $[CO]_{electrode}$ values represent upper bounds obtained under the assumptions described in the text.

Fe-*p*-TMA Concentration	Current Density (mA/cm^2)	FE_{CO} (%)	j_{co} (mA/cm^2)	j_{DK} (μA/cm^2)	μ (μm)	$[CO]_{electrode}$ (mM)
0 mM	5.21	0.07	0.004	-	-	0.01
0.05 mM	4.75	2.29	0.11	0.98	0.82	0.26
0.1 mM	3.21	11.93	0.38	1.95	0.48	0.91
0.25 mM	2.00	57.06	1.14	4.88	0.40	2.71
0.5 mM	2.99	88.16	2.64	9.75	0.34	6.26

References

S1. Costentin, C.; Robert, M.; Saveant, J.-M.; Tatin, A.; Azcarate, I. Selective Porphyrin-Catalyzed Electrochemical Reduction of CO_2 into CO in Water. 20180023204, January 25, 2018.

S2. Lobaccaro, P.; Singh, M. R.; Clark, E. L.; Kwon, Y.; Bell, A. T.; Ager, J. W. Effects of Temperature and Gas–Liquid Mass Transfer on the Operation of Small Electrochemical Cells for the Quantitative Evaluation of CO_2 Reduction Electrocatalysts. *Phys. Chem. Chem. Phys.* **2016**, *18* (38), 26777–26785.

S3. Zhang, B. A.; Ozel, T.; Elias, J. S.; Costentin, C.; Nocera, D. G. Interplay of Homogeneous Reactions, Mass Transport, and Kinetics in Determining Selectivity of the Reduction of CO_2 on Gold Electrodes. *ACS Cent. Sci.* **2019**, *5* (6), 1097–1105.

S4. Hara, K.; Kudo, A.; Sakata, T. Electrochemical CO_2 Reduction on a Glassy Carbon Electrode under High Pressure. *J. Electroanal. Chem.* **1997**, 421 (1), 1–4.

S5. Nakagawa, S.; Kudo, A.; Azuma, M.; Sakata, T. Effect of Pressure on the Electrochemical Reduction of CO2 on Group VIII Metal Electrodes. *J. Electroanal. Chem.* **1991**, *308* (1), 339–343.

S6. Hwang, S. Y.; Maeng, J. Y.; Yoon, I.; Myung, C. W.; Rhee, C. K.; Sohn, Y. Electrochemical Fischer-Tropsch Chemistry across Transition Metals: A Paradigm Shift in Sustainable Liquid Fuel Production. *Nano Energy* **2024**, 128, 109881.

S7. Hara, K.; Kudo, A.; Sakata, T. Electrochemical Reduction of High Pressure Carbon Dioxide on Fe Electrodes at Large Current Density. *J. Electroanal. Chem.* **1995**, 386 (1), 257–260.

S8. Deeba, R.; Collard, A.; Rollin, C.; Molton, F.; Chardon-Noblat, S.; Costentin, C. Controlled Potential Electrolysis: Transition from Fast to Slow Regimes in Homogeneous Molecular Catalysis. Application to the Electroreduction of CO2 Catalyzed by Iron Porphyrin. *ChemElectroChem* **2023**, *10* (20), e202300350.

S9. Salamé, A.; Cheah, M. H.; Bonin, J.; Robert, M.; Anxolabéhère-Mallart, E. Operando Spectroelectrochemistry Unravels the Mechanism of CO_2 Electrocatalytic Reduction by an Fe Porphyrin. *Angew. Chem. Int. Ed.* **2024**, 63 (51), e202412417.

S10. Wise, D. L.; Houghton, G. Diffusion Coefficients of Neon, Krypton, Xenon, Carbon Monoxide and Nitric Oxide in Water at 10–60°C. *Chem. Eng. Sci.* **1968**, *23* (10), 1211–1216.
